# Nuclear ADP-ribosylation drives IFNγ-dependent STAT1α enhancer formation in macrophages

**DOI:** 10.1038/s41467-021-24225-2

**Published:** 2021-06-24

**Authors:** Rebecca Gupte, Tulip Nandu, W. Lee Kraus

**Affiliations:** 1 Laboratory of Signaling and Gene Regulation, Cecil H. and Ida Green Center for Reproductive Biology Sciences, University of Texas Southwestern Medical Center, Dallas, TX USA; 2 Division of Basic Research, Department of Obstetrics and Gynecology, University of Texas Southwestern Medical Center, Dallas, TX USA

**Keywords:** Chemical modification, Genomic analysis, Monocytes and macrophages, Transcriptional regulatory elements

## Abstract

STAT1α is a key transcription factor driving pro-inflammatory responses in macrophages. We found that the interferon gamma (IFNγ)-regulated transcriptional program in macrophages is controlled by ADP-ribosylation (ADPRylation) of STAT1α, a post-translational modification resulting in the site-specific covalent attachment of ADP-ribose moieties. PARP-1, the major nuclear poly(ADP-ribose) polymerase (PARP), supports IFNγ-stimulated enhancer formation by regulating the genome-wide binding and IFNγ-dependent transcriptional activation of STAT1α. It does so by ADPRylating STAT1α on specific residues in its DNA-binding domain (DBD) and transcription activation (TA) domain. ADPRylation of the DBD controls STAT1α binding to its cognate DNA elements, whereas ADPRylation of the TA domain regulates enhancer activation by modulating STAT1α phosphorylation and p300 acetyltransferase activity. Loss of ADPRylation at either site leads to diminished IFNγ-dependent transcription and downstream pro-inflammatory responses. We conclude that PARP-1-mediated ADPRylation of STAT1α drives distinct enhancer activation mechanisms and is a critical regulator of inflammatory responses in macrophages.

## Abstract (web summary)

STAT1a is required for pro-inflammatory responses in macrophages. Here the authors reveal that post-translational modification of STAT1a, ADPribosylation, plays a critical role in enhancer formation and activation, thus modulating IFNγ-stimulated inflammatory responses in macrophages.

## Introduction

Cells of the innate immune system, including macrophages, monocytes, and dendritic cells, are typically the first responders to microbial infection and are responsible for pathogen clearance^1^. Macrophages respond to stimuli, such as chemokines, cytokines, and pathogen-associated molecules, by triggering downstream intracellular signaling events that lead to the expression of genes encoding inflammatory mediators^2^. Post-translational modifications (PTMs) that modulate protein functions are key regulators of the components of immune signal transduction pathways in macrophages^3^. While the role of phosphorylation has been well-documented in regulating immune responses, emerging evidence shows that other PTMs, such as acetylation, methylation, citrullination, and nitrosylation, are also major contributors to inflammatory signaling^3^. Recent studies have also linked ADP-ribosylation (ADPRylation)—a PTM derived from nicotinamide adenine dinucleotide (NAD^+^)—to immune responses in macrophages^4^.

ADPRylation is mediated by members of the poly(ADP-ribose) polymerase (PARP) family of enzymes, which catalyze the transfer of ADP-ribose from NAD^+^ to target proteins to alter their functions^5,6^. Historically, the focus in the field has been on PARP-1, the most abundant and ubiquitous member of the PAR family, and its role in DNA damage detection and repair through poly(ADP-ribosyl)ation (PARylation)^7,8^. Recent studies, however, have revealed the importance of PARP-1 in transcriptional regulation in a variety of biological systems^9^. The mechanisms of PARP-1-mediated gene regulation include the modulation of histone modifications and chromatin structure and serving as a transcriptional coregulator^10,11^. Alternatively, PARP-1 can interact with, ADP-ribosylate, and modulate the activity of multiple transcription factors, including B-MYB, AP-2, HIF-1α, and C/EBPβ^12–15^. The actions of PARP-1 in gene regulation are ultimately reflected in modified cellular signaling pathways and alteration in physiological outcomes, such as stress and immune responses, circadian rhythms, and metabolism^16^.

Recently, there has been increasing interest in the potential use of PARP inhibitors as therapeutics for autoimmune and inflammatory diseases^17^. Early studies showed that *Parp1* null mice are resistant to septic shock due to decreased serum levels of pro-inflammatory cytokines^18^. PARP-1 also been shown to potentiate inflammation and innate immune responses by modulating NF-κB activity^19–23^. However, the role of PARP-1 in regulating the activity of specific targets in different immune cell types, such as macrophages, and the implications for disease physiology remains to be explored.

One of the major cytokines that activates macrophages is interferon gamma (IFNγ)^24^ and the modulation of gene expression by IFNγ occurs primarily through the Signal Transducers and Activators of Transcription (STAT) family member, STAT1. Indeed, the loss of functional STAT1 in patients has been linked to increased susceptibility to mycobacteria^25,26^ and viral infections^27^. The binding of extracellular IFNγ to its cognate receptor triggers the JAK-STAT signaling cascade and leads to phosphorylation of STAT1 at Tyrosine 701^28^. Tyrosine phosphorylated STAT1 can homodimerize and translocate to the nucleus, where it can bind gamma-activated site (GAS) DNA motifs^29^. Most cells express two different STAT1 isoforms, STAT1α and STAT1β, the latter being a C-terminally truncated form^30^. IFNγ-stimulated nuclear STAT1α, once bound to genomic DNA, is phosphorylated at a second site, Serine 727^31^. S727 phosphorylation promotes the recruitment of coregulators, such as CBP/p300, to DNA-bound STAT1α, leading to enhancer formation, which is marked by histone H3 lysine K27 acetylation (H3K27ac)^32,33^. Phosphorylation of IFNγ-activated STAT1α on both Y701 and S727 is critical for optimal gene activation^31^. STAT1α-bound enhancers are critical for maintaining both acute and prolonged inflammatory responses^34^. The STAT1α-regulated transcriptome includes genes encoding antiviral proteins, microbicidal molecules, phagocytic receptors, chemokines, cytokines, and antigen-presenting molecules, which are prototypical of macrophages polarized towards the pro-inflammatory phenotype^29^.

Here we identified PARP-1 as a key regulator of IFNγ-dependent signaling in macrophages by posttranslationally modifying STAT1α through ADPRylation. Furthermore, we show that ADPRylation of STAT1α has profound effects on inflammatory phenotypes in macrophages by regulating STAT1α enhancer formation and transcriptional activation.

## Results

### PARP-1 catalytic activity mediates the IFNγ-dependent transcriptional program in macrophages

**Fig. 1:**
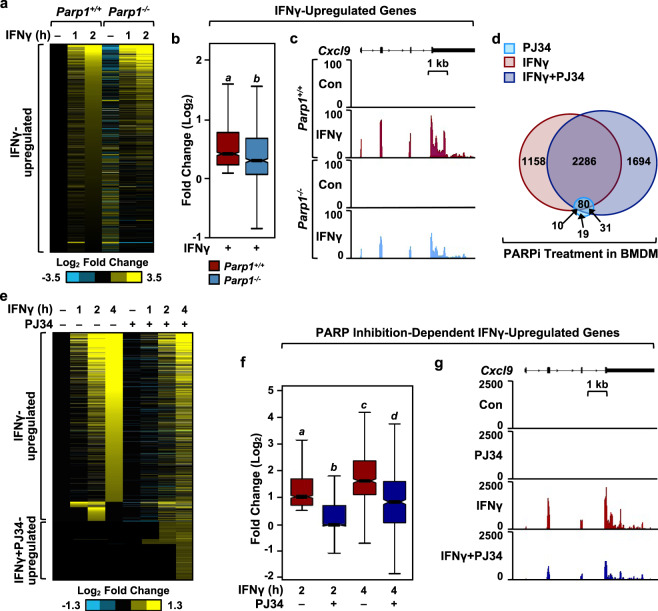
PARP-1 regulates IFNγ-dependent gene expression in bone marrow-derived macrophages (BMDMs). **a** Heatmap of RNA-seq data representing changes in the expression of IFNγ-regulated genes from mRNA-seq in BMDMs from wild-type (*Parp1*^*+/+*^) or *Parp1* knockout (*Parp1*^*-/-*^) mice. The cells were treated with IFNγ for the indicated times. **b**, **c** Box plots (**b**) and browser tracks (**c**) illustrating IFNγ-stimulated gene expression in *Parp1*^*+/+*^ or *Parp1*^*-/-*^ mice. BMDM cells were treated with IFNγ for 2 h and steady-state mRNA levels from RNA-seq were expressed as fold change relative to the untreated control. Boxes represent 25^th^–75^th^ percentile (line at median) with whiskers at 1.5*IQR. Boxes marked with different letters are significantly different from each other (Wilcoxon Signed-Rank test; *p* < 2.2 × 10^−16^). Box plots represent 960 genes. **d** Venn diagrams showing differentially regulated genes from RNA-seq in BMDMs upon treatment with PJ34 (light blue), IFNγ (red), or IFNγ + PJ34 (blue). Numbers indicate the number of differentially regulated genes compared to the untreated control. **e** Heatmap of RNA-seq data representing the changes in gene expression of IFNγ- regulated genes upon co-treatment with PJ34. **f**, **g** PARP-1 catalytic activity is required for IFNγ-dependent gene expression in BMDMs. Box plots (**f**) and browser tracks (**g**) representing changes in gene expression from RNA-seq upon IFNγ treatment ± PJ34 (*n* = 1053 genes; Wilcoxon Signed-Rank test; *p* < 2.2 × 10^−16^). Boxes represent 25^th^–75^th^ percentile (line at median) with whiskers at 1.5*IQR.

PARP-1 has been implicated in the regulation of gene expression in different cell types through either catalytically-dependent or catalytically-independent mechanisms^9^. To determine the role of PARP-1 in regulating IFNγ-stimulated transcription in macrophages, we performed RNA-sequencing (RNA-seq) in primary bone marrow-derived macrophages (BMDMs) isolated from wild-type (*Parp1*^*+/+*^) or *Parp1* null (*Parp1*^*-/-*^) mice. We observed significant alterations in the IFNγ-stimulated transcriptome over a time course of treatment (0, 1, 2 h) in BMDMs upon loss of PARP-1 (Fig. 1a). Moreover, loss of PARP-1 resulted in attenuated expression of IFNγ-upregulated genes (Fig. 1b, c). To determine whether the catalytic activity of PARP-1 is required for the regulation of IFNγ signaling in macrophages, we treated BMDMs with the PARP inhibitor PJ34 prior to a time course of IFNγ stimulation. PJ34 treatment resulted in a dramatic modulation in the expression of IFNγ-regulated genes, with little effect on basal gene expression (Fig. 1d, e). Consistent with the loss of PARP-1 protein through genetic ablation, inhibition of PARP-1 catalytic activity resulted in attenuated expression of the IFNγ- stimulated transcriptome (Fig. 1f, g). Surprisingly, we noticed that a subset of genes were upregulated only in the presence of both IFNγ and PJ34 (Fig. 1e; Supplementary Fig. 1a, b). This suggests a mode of regulation that could be distinct from the IFNγ-upregulated genes. We observed the same requirement for PARP-1 catalytic activity in regulating IFNγ-stimulated gene expression in immortalized BMDMs (iBMDMs) as well (Supplementary Fig. 1c, d). Additionally, we observed that treatment of iBMDMs with a different PARP inhibitor, veliparib, also attenuated the expression of IFNγ-induced chemokines, *Ccl12* and *Ccl7* (Supplementary Fig. 1e). Taken together, these data reveal a critical role for PARP-1 in regulating IFNγ-mediated gene expression in macrophages.

### PARP-1-dependent ADPRylation controls the IFNγ-induced STAT1α cistrome

**Fig. 2:**
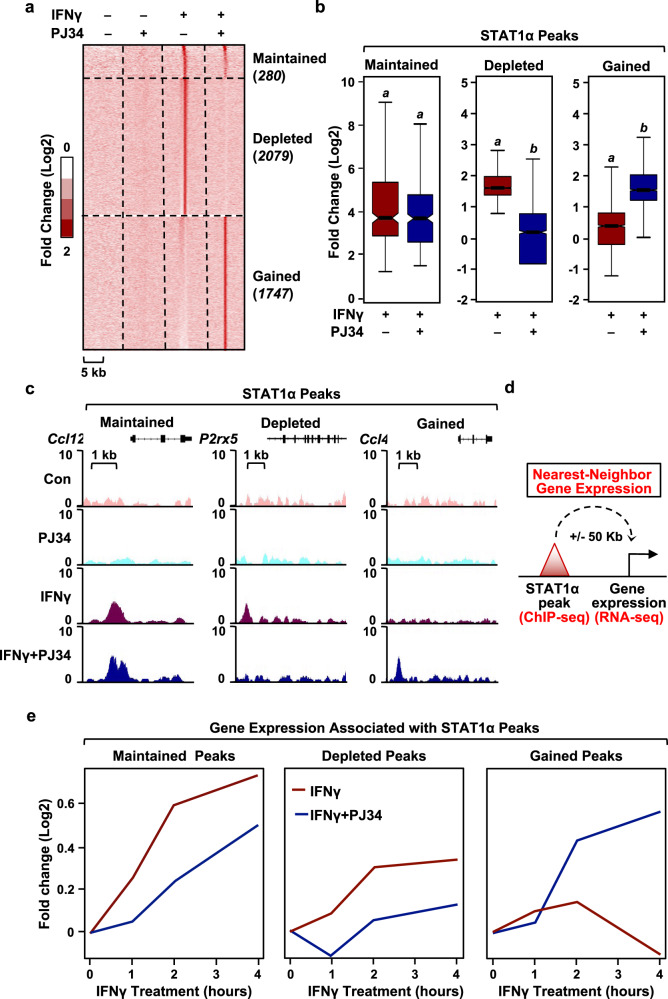
Inhibition of PARP-1 catalytic activity results in genome-wide redistribution of STAT1α. **a** Heat map of ChIP-seq data representing STAT1α binding in BMDMs treated with IFNγ ± PJ34. BMDMs were treated with IFNγ for 1 h and ChIP-seq was performed using STAT1 antibody. Enrichment of peaks is shown relative to the untreated control. **b**, **c** Box plots (**b**) and browser tracks (**c**) representing ‘maintained,’ ‘depleted,’ and ‘gained’ STAT1α peaks from ChIP-seq data. A cutoff of 1x MAD (median absolute deviation) was used to define ‘gained’ and ‘depleted’ peaks. ‘Maintained’ peaks were defined with a cutoff of 0.5x MAD (Wilcoxon Signed-Rank test; *p* < 2.2 × 10^-16^). Number of peaks for box plots was indicated in (**a**). Boxes represent 25^th^–75^th^ percentile (line at median) with whiskers at 1.5*IQR. **d** A schematic diagram showing the integration of ChIP-seq data with RNA-seq to correlate STAT1α binding with changes in gene expression in BMDMs. **e** PARP-1-dependent changes in STAT1α binding correlate with altered transcriptional outcomes. The nearest neighbor gene expression for each category of STAT1α peaks was calculated as shown in (**d**). The line plots represent the fold change in gene expression upon IFNγ treatment ± PJ34 from the RNA-seq assays shown in Fig. 1. The mRNA levels are expressed as fold change over the untreated control.

Activation of gene expression by IFNγ occurs primarily by promoting the binding of dimerized STAT1α to its response elements^28^. This led us to hypothesize that PARP-1 might regulate the expression of IFNγ-stimulated genes by modulating STAT1α binding to the genome. We used chromatin immunoprecipitation sequencing (ChIP-seq) to determine the effects of inhibiting PARP-1 catalytic activity on IFNγ-dependent STAT1α binding genome-wide in BMDMs (Fig. 2a). Surprisingly, short term PARP inhibition with PJ34 significantly altered IFNγ-dependent STAT1α genomic localization compared to IFNγ treatment alone (Fig. 2a). Based on the effects observed with PJ34 treatment, we categorized the STAT1α binding sites into three distinct classes; having ‘maintained’, ‘depleted’, ‘gained’ peaks (Fig. 2b, c; Supplementary Fig. 2). This analysis shows that inhibition of PARP catalytic activity alters the IFNγ-dependent STAT1α cistrome illustrated by the striking loss and gain of binding sites. To determine the effects of PJ34-induced changes in STAT1α binding on downstream gene expression, we assessed the expression changes in the genes nearest to the STAT1α binding sites upon IFNγ ± PJ34 treatment (Fig. 2d). As expected, the genes nearest to ‘depleted’ STAT1α binding sites showed attenuated expression in the presence of PJ34, while the genes nearest to ‘gained’ STAT1α binding sites showed enhanced expression in the presence of PJ34 (Fig. 2e). Unexpectedly, the genes nearest to the ‘maintained’ STAT1α binding sites showed decreased IFNγ-stimulated expression upon PARP inhibition (Fig. 2e), suggesting an additional mode of regulation of STAT1α activity by PARP-1.

### ADPRylation promotes transcriptional activation of STAT1α by modulating its phosphorylation

**Fig. 3:**
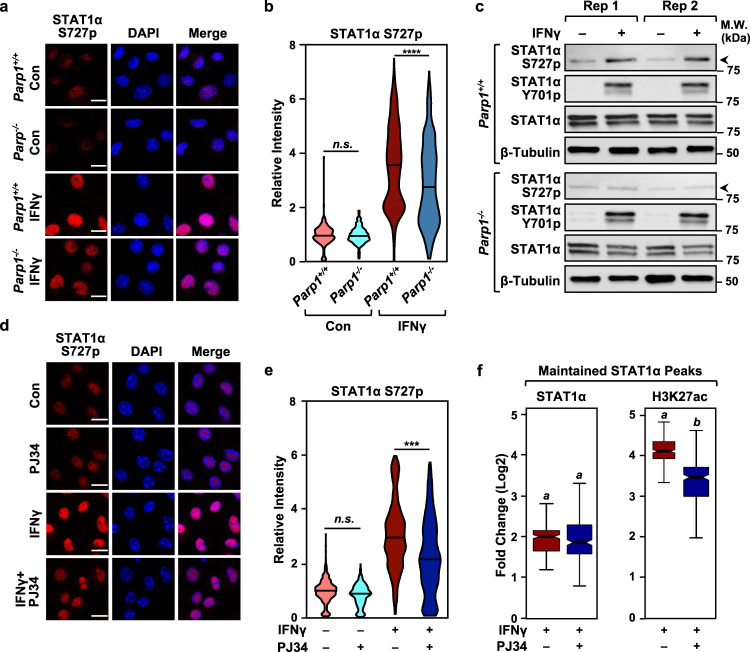
PARP-1 catalytic activity promotes the phosphorylation of STAT1α on Serine 727. **a** PARP-1 deletion attenuates STAT1α phosphorylation at S727. Immunofluorescent staining for phospho-STAT1α (S727p) was performed in BMDMs collected from wild-type (*Parp1*^*+/+*^) or *Parp1* knockout (*Parp1*^*-/-*^) mice treated with IFNγ (1 h). Nuclei were visualized with DAPI staining. Scale bar: 10 μm. **b** Violin plots showing quantification of the immunofluorescence data from (**a**) in BMDMs from *Parp1*^*+/+*^ (*n* = 3) and *Parp1*^*-/-*^ (*n* = 3) mice (one-way ANOVA followed by Tukey’s multiple comparison tests; **** < 0.0001; n.s., not significant at 0.05). **c** Immunoblots showing the relative levels of STAT1α S727p, STAT1α Y701p, total STAT1α and Tubulin in BMDMs from *Parp1*^*+/+*^ and *Parp1*^*-/-*^ mice. Rep1 and Rep2 represent two independent biological replicates. Uncropped immunoblots are provided as a Source Data file. **d** Inhibition of PARP-1 catalytic activity by PJ34 blocks STAT1α S727 phosphorylation. Immunofluorescent staining was performed as in (**a**). BMDMs were treated with IFNγ (1 h) ± PJ34. Nuclei were visualized with DAPI staining. Scale bar: 10 μm. **e** Violin plots showing quantification of the immunofluorescence data from (**d**) for BMDMs from 3 mice for each treatment (one-way ANOVA followed by Tukey’s multiple comparison test; *** < 0.0001). **f** Inhibition of PARP-1 catalytic activity by PJ34 results in reduced enrichment of H3K27ac *(right)* levels at maintained STAT1α binding sites *(left)*. ChIP-seq for STAT1α and H3K27ac was carried out in BMDMs treated with IFNγ (1 h) ± PJ34 (*n* = 252 peaks; Wilcoxon Signed-Rank test; *p* < 2.2 × 10^-16^). Boxes represent 25^th^–75^th^ percentile (line at median) with whiskers at 1.5*IQR.

IFNγ stimulation induces the phosphorylation of STAT1α at two distinct sites; first at Y701 to promote nuclear localization, then at S727 to promote transcriptional activation^35^. Phosphorylation of chromatin-bound STAT1α at S727 promotes p300/CBP recruitment, histone acetylation (e.g., H3K27ac), and target gene activation^31,33^. Indeed, mice expressing a S727 phosphorylation-defective mutant of STAT1α show reduced responsiveness to IFNγ^31^. We found that BMDMs isolated from *Parp1*^*-/-*^ mice exhibited reduced IFNγ-induced phosphorylation of S727 on STAT1α compared to BMDMs isolated from wild-type mice (Fig. 3a–c; Supplementary Fig. 3a). Additionally, we observed no significant changes in Y701 phosphorylation in response to loss of PARP-1, as opposed to a reduction in S727 phosphorylation observed in the same lysates (Fig. 3c; Supplementary Fig. 3b). In agreement with the changes in gene expression observed in Fig. 1, inhibiting PARP-1 activity with PJ34 treatment similarly attenuated STAT1α S727 phosphorylation in BMDMs (Fig. 3d, e). Cotreatment of IFNγ treated-BMDMs with PJ34, however, produced no differences in the level of nuclear STAT1α (Supplementary Fig. 4a, b), thus indicating that the reduction in S727 phosphorylation is not due to impaired translocation of STAT1α to the nucleus. We confirmed this cross-talk between STAT1α ADPRylation and phosphorylation in other systems, including iBMDMs and human THP-1 macrophage-like cells (Supplementary Fig. 4c, d). Similarly, we observed that veliparib also inhibited the IFNγ-stimulated phosphorylation of STAT1α on S727 (Supplementary Fig. 4e). These results, taken together with a similar observation made in PJ34-treated cells, further support our claim that PARP-1 catalytic activity is critical for the regulation of IFNγ signaling through STAT1α. Furthermore, we saw no induction of STAT1α S727p upon bacterial lipopolysaccharide (LPS) treatment, thus confirming the specificity of our observations with regards to IFNγ signaling (Supplementary Fig. 4f).

The phosphorylation of STAT1α at S727 is required for recruitment of coregulators, such as p300 and CBP, which acetylate histones at enhancers (esp. H3K27) to effectively promote enhancer activity and target gene transcription. Using ChIP-seq in IFNγ-treated BMDMs, we observed reduced levels of H3K27ac at a set of ‘maintained’ STAT1α binding sites (i.e., enhancers) in the presence of PJ34 (Fig. 3f). These data show that ADPRylation of STAT1α is critical for its activation and subsequent coregulator recruitment. Taken together, the data showing PARP-1-dependent changes in STAT1α phosphorylation and genomic localiztion indicate that ADPRylation by PARP-1 can affect IFNγ-dependent gene expression by modulating distinct aspects of STAT1α activation.

### PARP-1 ADP-ribosylates STAT1α on distinct amino acid residues

**Fig. 4:**
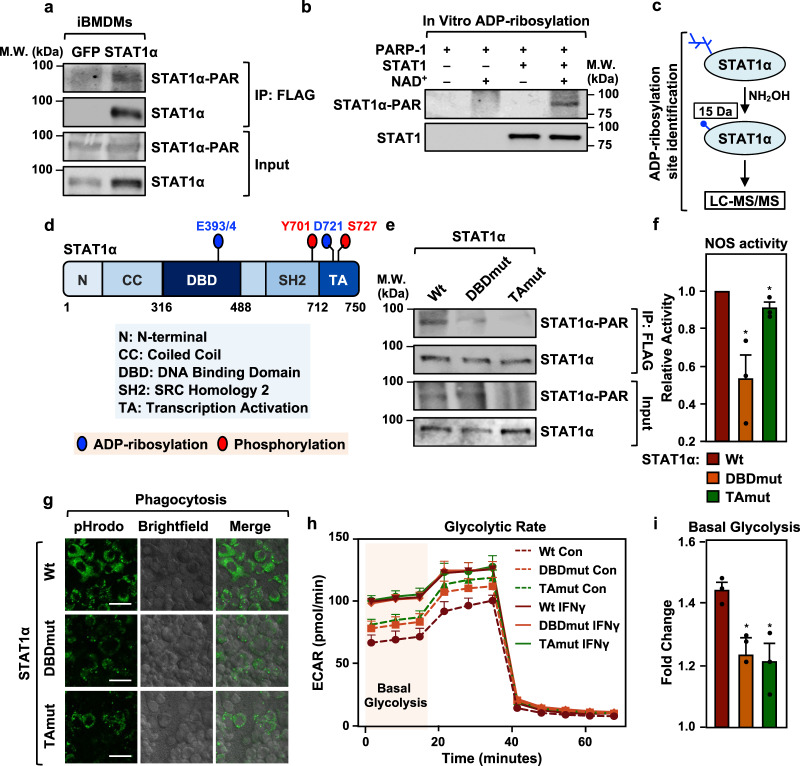
ADPRylation of STAT1α by PARP-1 at specific sites on the DNA-binding and transactivation domains is required for facilitating pro-inflammatory responses in macrophages. **a** STAT1α is ADPRylated in cells. Immunoblots showing ADPRylation of STAT1α in immortalized BMDMs (iBMDMs). Flag-tagged STAT1α was ectopically expressed in iBMDMs and immunoprecipitated using a Flag antibody. Flag-tagged GFP was used as a vector control. PAR levels were detected using an ADP-ribose detection reagent (WWE-Fc reagent). The immunoblots are representative of 3 independent experiments. Uncropped immunoblots are provided as a Source Data file. **b** Immunoblots showing ADPRylation of STAT1α by PARP-1 in vitro. In vitro ADPRylation reactions were setup as indicated. Recombinant PARP-1 and STAT1α expressed and purified from Sf9 insect cells were incubated with 100 μM NAD^+^. The immunoblots are representative of 3 independent experiments. Uncropped immunoblots are provided as a Source Data file. **c** Schematic representation of the protocol used for determining the sites of ADPRylation on STAT1α using mass spectrometry. **d** Schematic representation showing the sites of ADPRylation on STAT1α determined by mass spectrometry. ADPRylated glutamate and aspartate residues on STAT1α are indicated by blue circles and sites of phosphorylation are indicated by red circles. **e** Mutation of mass spectrometry-identified ADPRylation sites inhibits ADPRylation on STAT1α in IFNγ-treated iBMDMs. Mutations of the amino acids shown in (**d**) were engineered into full-length STAT1α. E393/4Q and D721N are indicated as DBDmut and TAmut, respectively. iBMDM cells were incubated with 250 μM NAD^+^ for the ADPRylation reactions in nuclei. Immunoblotting was performed as in (**a**). Uncropped immunoblots are provided as a Source Data file. **f** Nitric oxide synthase (NOS) activity assay measuring relative NOS levels in iBMDMs expressing Wt vs. ADPRylation-deficient STAT1α mutants. iBMDM cells were treated with IFNγ for 24 h (*n* = 3; Student’s two-tailed, unpaired t-test * = 0.0208 for Wt vs. DBDmut; * = 0.0410 for Wt vs. TAmut). Error bars represent mean ± SEM. **g** Loss of site-specific ADPRylation on the STAT1α DBD or TA domain results in reduced phagocytotic capacity in macrophages. Phagocytosis in iBMDMs was assayed using *S. aureus* bioparticles conjugated to pHrodo-green. The images are representative of 3 independent experiments. Scale bar: 44 μm. **h**, **i** Site-specific ADPRylation of STAT1α on its DBD or TA domain is required for IFNγ-stimulated increases in cellular glycolysis. **h** Glycolytic rate profile of iBMDMs expressing Wt or ADPRylation-deficient STAT1α mutants using Seahorse assays (*n* = 3). Error bars represent mean ± SEM. **i** Fold change in the amount of basal glycolysis observed upon IFNγ treatment (*n* = 3; two-tailed, unpaired t-test * = 0.0268 for Wtvs. DBDmut; * = 0.0207 for Wt vs. TAmut). Error bars represent mean ± SEM.

**Fig. 5:**
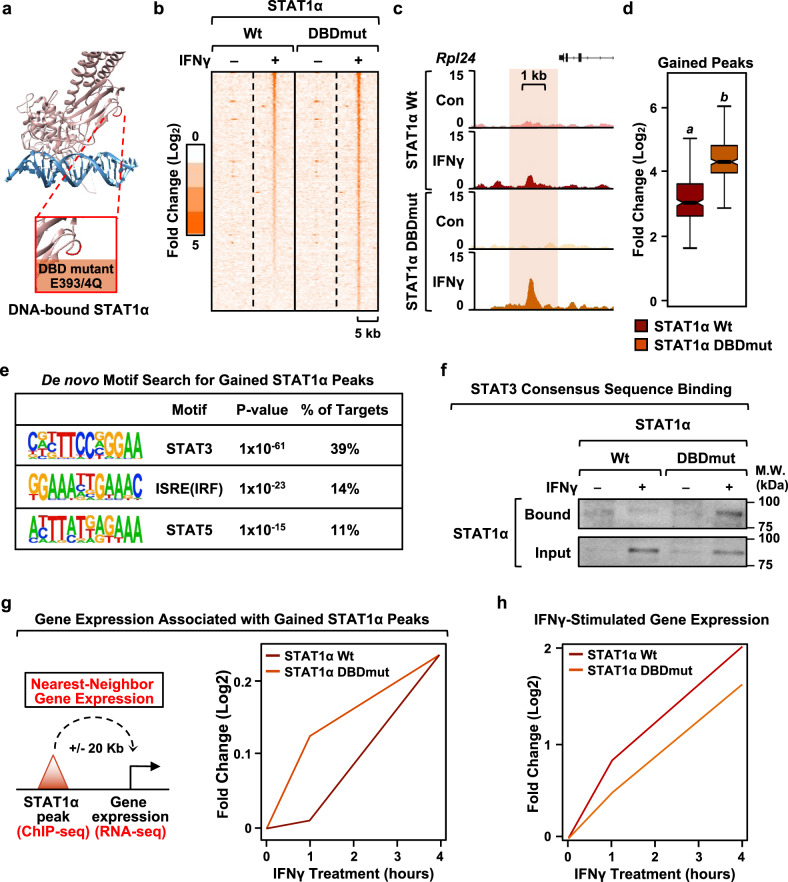
ADPRylation on its DBD restricts STAT1α binding to consensus motifs. **a** Structure of DNA-bound STAT1α showing sites of ADPRylation on the DBD. STAT1α is shown in pink and DNA is shown in blue. ADPRylated residues (E393 and E394) are highlighted in red in the expanded view. The structure is from Protein Data Bank (PDB) 1BF5. **b** Heatmap of ChIP-seq data showing STAT1α enrichment in the top 50% of ‘gained’ STAT1α peaks in iBMDMs expressing wild-type (Wt) or DBD mutant (DBDmut) STAT1α with concurrent shRNA-mediated knockdown of endogenous STAT1α. The cells were treated ± IFNγ for 1 h. ‘Gained’ peaks were defined using a 4x MAD cutoff. **c**, **d** Browser tracks (**c**) and box plots (**d**) of ChIP-seq data representing ‘gained’ STAT1α peaks in iBMDMs expressing DBDmut compared to iBMDMs expressing Wt STAT1α (*n* = 227 peaks; Wilcoxon Signed-Rank test; *p* < 2.2 × 10^-16^). Boxes represent 25^th^–75^th^ percentile (line at median) with whiskers at 1.5*IQR. **e** Motifs enriched at gained STAT1α binding sites (DBDmut relative to Wt STAT1α). De novo motif analysis was performed using MEME. The predicted motifs were matched to known motifs using TOMTOM. P-values were generated using default parameters in TOMTOM (see Methods). **f** Binding of STAT1α DBDmut to non-consensus motifs. STAT1α from iBMDMs expressing Wt or DBDmut was incubated with double-stranded DNA oligonucleotides containing a consensus STAT3 binding sequence. Bound material and input were analyzed by immunoblotting for STAT1α. Uncropped immunoblots are provided as a Source Data file. **g** Gene expression associated with gained STAT1α peaks. Line plots representing fold change in nearest neighbor gene expression upon IFNγ treatment from RNA-seq in iBMDMs expressing Wt or DBDmut STAT1α with concurrent shRNA-mediated knockdown of endogenous STAT1α. **h** Line plots representing fold change in IFNγ-stimulated gene expression in iBMDMs expressing Wt or DBDmut STAT1α relative to an untreated control.

To determine the mechanisms by which PARP-1 modulates STAT1α transcriptional activity, we first sought to determine if PARP-1 can ADPRylate STAT1α. Using immunoprecipitation coupled with immunoblotting in iBMDMs, we observed ADPRylation of STAT1α (Fig. 4a). In iBMDM cells ectopically expressing Flag-tagged STAT1α, we also observed STAT1α ADPRylation that was inhibited in the presence of PJ34 and correlated with the amount of nuclear STAT1α (Extended Data Fig. 5a). PARP-1 also specifically ADPRylated STAT1α in an in vitro assay with purified PARP-1 and STAT1α (Fig. 4b; Supplementary Fig. 5b). To gain better insights into the function of STAT1α ADPRylation by PARP-1, we determined the specific sites of modification on STAT1α. For this we immunoprecipitated Flag-tagged STAT1α ectopically expressed in IFNγ-treated HEK293T cells, as well as endogenous STAT1α from IFNγ-treated iBMDMs. In both experiments, the immunoprecipitated STAT1α was subjected to hydroxylamine treatment, which cleaves the ADP-ribose moiety from ADPRylated aspartate (Asp, D) and glutamate (Glu, E) residues, leaving a hydroxamic acid derivative attached to the amino acid side chain^9,36^. The resulting mass shift of 15.0109 Da can be identified by mass spectrometry (Fig. 4c). From this mass spectrometric analysis of STAT1α (both endogenous and ectopically expressed), we identified a number ADPRylated Asp and Glu residues on STAT1α including D721 in the transactivation (TA) domain and E393/4 in the DNA-binding domain (DBD) (ADPRylated E393 and E394 could not be distinguished due to ambiguity in the mass spectrometry assignments) (Fig. 5d; Supplementary Fig. 5c, d; Supplementary Data 1).

To explore the role of ADPRylation in regulating STAT1α function, we generated site-specific mutants where the Asp and Glu residues of interest were substituted with asparagine (Asn, N) and glutamine (Gln, Q), respectively. These substitutions prevent ADPRylation and mimic the unmodified forms of amino acids. Accordingly, we generated cell lines that express ADPRylation site mutants targeting the TA domain (D721N) and DBD (E393/4Q). To avoid any unintended side-effects due to long-term expression of the mutant STAT1α proteins, we used a doxycycline (Dox)-inducible expression system to generate iBMDM cell-lines expressing the different ADPRylation mutants. As expected, the mutant STAT1α proteins exhibited reduced levels of ADPRylation when expressed in cells compared to the wild-type (Wt) STAT1α protein (Fig. 4e; Supplementary Fig. 5e), thus confirming our mass-spectrometry results. Furthermore, we did not observe a consistent difference in the total ADPRylation levels in cells expressing either mutant, with or without IFNγ stimulation (Supplementary Fig. 5f).

### ADPRylation of STAT1α is required for inflammatory responses in macrophages

To explore the impact of loss of site-specific ADPRylation of STAT1α on inflammatory responses, we engineered iBMDMs to express Dox-inducible Wt, DBD mutant (DBDmut) and TA mutant (TAmut) STAT1α, as described above. This was done with coexpression of an shRNA targeting the 3′-UTR of the *Stat1* mRNA (shStat1) to knockdown endogenous STAT1α in order to prevent effects from the endogenous protein. In control cells, we expressed GFP together with a non-specific shRNA (shCon) (Supplementary Fig. 6). In these and subsequent experiments, we used these cell lines for all our molecular and cellular assays. Abrogation of site-specific ADPRylation on both the DBD and TA domain of STAT1α resulted in impaired induction of IFNγ-regulated genes in iBMDMs (Supplementary Fig. 7a). An examination of the ontologies of the genes affected revealed that they are involved in modulating innate immune and inflammatory responses (Supplementary Fig. 7b). These findings indicate a role for site-specific ADPRylation of STAT1α in regulating physiological pro-inflammatory responses in macrophages. To investigate this in more detail, we tested a variety of known macrophage inflammatory responses in iBMDMs ectopically expressing either Wt STAT1α or the site-specific ADPRylation defective mutants (DBDmut and TAmut) (Supplementary Fig. 8a).

Induction of nitric oxide synthase (NOS) is a well-known marker for macrophage activation towards a pro-inflammatory phenotype^37^. Importantly, iBMDMs expressing the STAT1α mutants had attenuated IFNγ-stimulated NOS activity in iBMDMs compared to Wt STAT1α (Fig. 4f). Phagocytosis of invading pathogens by macrophages is one of the first lines of defense mounted by the host and is a critical component of innate immune responses^38^. We tested the competence of innate responses in the presence of the ADPRylation-defective STAT1α mutants by evaluating their effects on phagocytosis. For this purpose, we added *S. aureus* bioparticles conjugated to a pH-dependent fluorescent tag (pHrodo) to Wt, DBDmut, or TAmut STAT1α-expressing iBMDMs. This system allowed us to quantify the intracellular fluorescence as a measure of the amount of phagocytosis. iBMDMs expressing either of the ADPRylation-defective STAT1α mutants showed a dramatic reduction in overall phagocytosis as compared to iBMDMs expressing Wt STAT1α (Fig. 4g). Quantification of the fraction of cells having phagocytosed *S. aureus* particles showed a significant decrease in iBMDMs expressing the STAT1α mutants compared to iBMDMs expressing Wt STAT1α (Supplementary Fig. 8b). We also performed the cell-based assays for which the outcomes were impacted by of loss of site specific ADPRylation of STAT1α (Fig. 4h, g) in the presence of PARP inhibitors. Both the amount of phagocytosis and NOS activity were significantly attenuated in the presence of PARP inhibitors (Supplementary Fig. 8c, d and f). These results show that PARP-1 catalytic inhibition phenocopies the effects of the site-specific mutants. Moreover, we did not observe PARP inhibitors further exacerbate the defects in phagocytosis seen in the loss of ADPRylation mutants (Supplementary Fig. 8e)

Finally, macrophage activation by pro-inflammatory mediators, such as LPS and IFNγ, has been shown to increase glycolysis as a way to ramp up cellular energy production to meet the demands of the inflammatory response^39^. Recent work has shown that cellular NAD^+^ levels are an important determinant of the glycolytic capacity of pro-inflammatory macrophages^40,41^. Conversely, mitochondrial respiration in IFNγ-stimulated macrophages is independent of the amount of NAD^+^^40^. Since PARP-1 is one of the primary consumers of NAD^+^ in the cell, we hypothesized that the NAD^+^-dependent regulation of glycolysis in activated macrophages could be occurring through PARP-1 catalyzed ADPRylation of substrates such as STAT1α. In accordance with this hypothesis, Seahorse analysis of glycolytic rates in IFNγ-stimulated iBMDMs showed that blocking site-specific ADPRylation of STAT1α resulted in reduced induction of glycolysis (Fig. 4h, i). Consistent with the depletion of cellular NAD^+^, site-specific ADPRylation of STAT1 was not required for IFNγ-stimulated mitochondrial respiration (Supplementary Fig. 8g, h).

Overall, we have shown that ADPRylation of STAT1α on the DBD and TA domain is integral for IFNγ-signaling and inflammatory responses in macrophages. However, the observation that STAT1α is ADPRylated on functionally distinct domains suggested to us the possibility that although these ADPRylation events regulate common phenotypic outcomes, they might be acting through different mechanisms. We investigated this possibility in detail by assaying the functional role of each ADPRylation site separately in the ensuing analyses.

### Site-specific ADPRylation of STAT1α on its DNA binding domain is required for binding to its cognate DNA elements

We mapped the DBD ADPRylation sites (E393/4) on the structure of DNA-bound STAT1α to help us deduce the potential effects of this modification on STAT1α function (Fig. 5a). These sites are located in a loop region that is not at the STAT1α:DNA interface. Thus, ADPRylation at these sites, which would link a large, negatively charged moiety to STAT1α via PARylation, would likely not inhibit STAT1α DNA binding, but could affect how or where it binds. To explore these possibilities, we mutated E393/4 to Gln so that we could assay the effects on DNA-binding. We observed a high correlation between STAT1α localization by ChIP-seq in the control (shCon/GFP; endogenous STAT1α) and wild-type STAT1αexpressing cells (shStat1/Wt STAT1α; ectopically expressed STAT1α) (Supplementary Fig. 9a), indicating that our ectopic expression system is reflective of the endogenous genomic events.

To investigate the effects of site-specific ADPRylation of the STAT1α DBD, we compared the genomic localization of STAT1α Wt and DBDmut in response to treatment with IFNγ. While we observed all expected outcomes (i.e., gained, maintained, lost STAT1α peaks), we were surprised by the large cohort of sites exhibiting a dramatic increase in occupancy with the STAT1α DBDmut compared to Wt (Fig. 5b–d). When we examined these sites in greater detail, we observed an enrichment of motifs for STAT3, IRF, and STAT5, but not STAT1α (Fig. 5e), suggesting that STAT1α DBDmut is redirected to DNA sequences that are distinct from its typical consensus sites. In agreement with this observation, STAT1α DBDmut was able to bind a double-stranded DNA oligonucleotide containing a STAT3 consensus motif more strongly than STAT1α Wt (Fig. 5f; Supplementary Fig. 9b). This implicates site-specific ADPRylation of the STAT1α DBD as a requirement for proper response element recognition by preventing non-specific binding of STAT1α to other motifs. To further support the role of PARP-1 in mediating this effect, we performed the STAT3 oligo-binding assay in iBMDMs ectopically expressing Wt STAT1α in the presence or absence of PJ34 treatment. We observed a robust increase in STAT1α binding to the consensus STAT3 sites upon PJ34 treatment (Supplementary Fig. 9c). Based on these data, we conclude that the loss of STAT1α ADPRylation, specifically on its DBD, enhances the ability of STAT1α to bind STAT3 consensus sequences. Given the role of STAT3 in eliciting anti-inflammatory responses in macrophages^42^, some of the observed effects on inflammatory macrophage phenotypes (Fig. 4f–i) and gene expression (Supplementary Fig. 7b) could be due to the aberrant activation of STAT3 in the DBDmut expressing cells instead of STAT1α-IFNγ signaling.

To test the functional consequences of this ADPRylation event, we examined changes in gene expression induced by the STAT1α DBDmut. We determined the expression of genes nearest to the ‘gained’ binding sites (DBDmut versus Wt) (Fig. 5g). These genes showed enhanced expression in the STAT1α DBDmut-expressing cells compared to the STAT1α Wt-expressing cells. In contrast, genes stimulated by IFNγ in STAT1α Wt-expressing cells exhibited markedly attenuated expression in the STAT1α DBDmut-expressing cells (Fig. 5h). Taken together, these data suggest that loss of site-specific ADPRylation in the DBD redistributes STAT1α across the genome, leading to the acquisition of new target genes and increased aberrant gene expression, while at the same time diminishing the expression of IFNγ-induced genes. Overall, our results make a compelling argument for site-specific ADPRylation of the STAT1α DBD as a requirement for proper formation of IFNγ-responsive enhancers and, consequently, as a driver of the IFNγ-dependent transcriptional program in macrophages.

### ADPRylation of STAT1α at D721 in its TA domain modulates transcriptional activation by regulating S727 phosphorylation and p300 activity

**Fig. 6:**
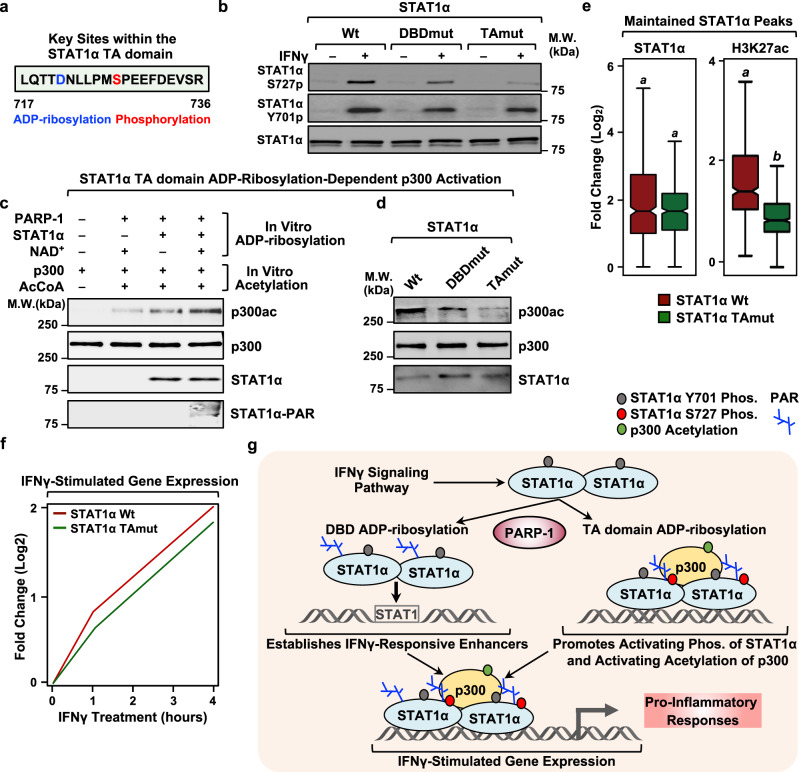
ADPRylation on its TA domain is required for IFNγ-dependent phosphorylation of STAT1α and activation of p300. **a** Amino acid sequence showing the ADPRylation (blue) and phosphorylation (red) sites in the TA domain of STAT1α. **b** ADPRylation at D721 in the TA domain of STAT1α is required for IFNγ-dependent phosphorylation. Immunoblots showing total STAT1α, STAT1α S727p and STAT1α Y701p from iBMDMs expressing Wt, DBDmut, or TAmut STAT1α. Uncropped immunoblots are provided as a Source Data file. **c** ADPRylation of STAT1α stimulates p300 autoacetylation. Immunoblots showing the acetylation of p300 from in vitro reactions performed in the presence of ADPRylated STAT1α under the conditions indicated. The immunoblots are representative of 3 independent experiments. Uncropped immunoblots are provided as a Source Data file. **d** ADPRylation of STAT1α on its TA domain is required for p300 autoacetylation. Immunoblots show autoacetylation of p300 in the presence of STAT1α Wt, DBDmut or TAmut from ADPRylation reactions with PARP-1 as indicated. The immunoblots are representative of 3 independent experiments. Uncropped immunoblots are provided as a Source Data file. **e** Loss of ADPRylation on the STAT1α TA domain results in reduced H3K27ac levels at maintained STAT1α binding sites. Box plots of ChIP-seq data showing STAT1α and H3K27ac enrichment in Wt- or TAmut-expressing iBMDMs (*n* = 492 peaks; Wilcoxon Signed-Rank test; *p* < 2.2 × 10^-16^). Boxes represent 25^th^–75^th^ percentile (line at median) with whiskers at 1.5*IQR. The cells were treated with IFNγ for 1 h. **f** Line plots representing fold change in IFNγ-stimulated gene expression in iBMDMs expressing Wt or TAmut STAT1α relative to an untreated control. The iBMDMs ectopically expressing Wt or TAmut STAT1α had concurrent shRNA-mediated knockdown of endogenous STAT1α. **g** Model showing the regulation of pro-inflammatory responses in macrophages by PARP-1-mediated site-specific ADPRylation of STAT1α. See the text for details.

Since the activating phosphorylation of STAT1α at S727 is dependent on ADPRylation driven by PARP-1 (Fig. 3a, d), we hypothesized that this phosphorylation could be dependent on site-specific ADPRylation of STAT1α. Our previous work has identified a proteome-wide link between site-specific Asp and Glu ADPRylation and nearby phosphorylation events^43^. Interestingly, one of the ADPRylated residues that we identified in STAT1α, D721, is in close proximity to the S727 phosphorylation site (Fig. 6a), suggesting a potential for cross-talk between the modifications at these two sites. To explore this possibility, we used the iBMDMs expressing Wt and TAmut STAT1α described above (Supplementary Fig. 6) to determine the effects of loss of D721 ADPRylation on S727 phosphorylation and STAT1α activity. While loss of STAT1α DBD ADPRylation (i.e., with DBDmut) did not have a significant effect on STAT1α S727 phosphorylation, mutation of the TA domain ADPRylation site (D721N; TAmut) caused a striking loss of phosphorylation (Fig. 6b; Supplementary Fig. 10a, b). Importantly, we did not observe changes in the levels of Y701p with either the DBDmut or the TAmut, compared to Wt (Fig. 6b), thus underscoring the specificity of the cross-talk between the D721 ADPRylation and S727 phosphorylation.

Phosphorylation of STAT1α at S727 has previously been implicated in the recruitment of p300 to STAT1α-bound enhancers^31,33^. A recent study showed that the interaction of p300 with STAT1α potentiated the autoacetylation and activation of p300 in a manner that was dependent on the STAT1α TA domain^44^. Based on these observations, we determined whether ADPRylation of STAT1α at D721 is critical for p300 activation. Purified STAT1α that was ADPRylated in vitro in the presence of NAD^+^ and PARP-1, induced the autoacetylation of purified recombinant p300 (Supplementary Fig. 10c) in the presence of acetyl-coA (Fig. 6c). Importantly, non-ADPRylated STAT1α or PARP-1 alone was insufficient to promote p300 autoacetylation (Fig. 6c). Intriguingly, mutation of the TA domain ADPRylation site (D721N) inhibited the p300 autoacetylation (Fig. 6d). Interestingly, we found that while p300 does acetylate PARP-1, ADPRylation of PARP-1 inhibits its acetylation (Supplementary Fig. 10d). This is an interesting result that provides insight into the dynamics of post-translational modifications on PARP-1. To separately assess the requirement of STAT1α S727p for p300 activation, we generated an S727A mutant, which is defective in phosphorylation of the TA domain. As with the previous p300 activity assays, we purified this mutant from mammalian cells and subjected it to in vitro ADPRylated, followed by in vitro acetylation in the presence of p300. We observed that the S727A mutant on its own can inhibit p300 auto-activation as well (Supplementary Fig. 10e). From this, we conclude that the optimal activation of p300 by STAT1α likely requires both ADPRylation and phosphorylation on the TA domain. These results highlight an exciting connection between site-specific ADPRylation of STAT1α and the acetyltransferase activity of p300.

Phosphorylation of STAT1α at S727 promotes the recruitment and activation of p300, leading to increased levels of H3K27ac at IFNγ-regulated enhancers^31–33,44^. As noted above, inhibition of ADPRylation in macrophages reduced the levels of H3K27ac at STAT1α-bound enhancers (Fig. 3f). To determine if this is mediated by the D721-ADPR → S727p → p300 activation pathway, we assessed the impact of mutation of the TA domain ADPRylation site (TAmut) on STAT1α binding and H3K27ac levels genome-wide by ChIP-seq. While mutating the D721 residue had no discernable impact on STAT1α recruitment to the enhancers (Fig. 6e; Supplementary Fig. 10f, g), it was associated with diminished levels of H3K27ac at the enhancers (Fig. 6e; Supplementary Fig. 10f). This attenuation of enhancer activation, as assessed by the levels of H3K27ac, was further reflected in the reduced expression of IFNγ-stimulated genes in the presence of the TAmut compared to Wt STAT1α (Fig. 6f). Collectively, these data highlight the importance of cross-talk between site-specific ADPRylation at D721 and phosphorylation at S727 in the TA domain on the p300-dependent activation of STAT1α enhancers and downstream pro-inflammatory gene expression.

In sum, we evaluated different aspects of macrophage-driven pro-inflammatory responses and determined that site-specific ADPRylation of STAT1α in the DBD and TA domain is critical for mediating all of these responses (Fig. 6g). These results support a role for PARP-1 as an integral part of the cellular signaling pathways involved in innate immunity.

## Discussion

Activation of STAT1α by IFNγ leads to the induction of a transcriptional program that is coordinated, in a large part, by post-translational modifications of STAT1α. Herein, we identified PARP-1-mediated ADPRylation of STAT1α as a regulator of the IFNγ-regulated transcriptional program in macrophages. Depletion of PARP-1 protein or inhibition of its catalytic activity impairs IFNγ-stimulated gene expression in macrophages by altering the genomic binding and phosphorylation of STAT1α. PARP-1 mediates these effects by ADPRylating STAT1α on specific sites in its DBD and TA domain, with distinct functional consequences. The former controls STAT1α binding to DNA, while the latter regulates STAT1α transcriptional activity, enhancer formation, and p300 acetyltransferase activity. Both ADPRylation events are required for STAT1α-mediated pro-inflammatory biological responses in macrophages, such as increases in phagocytosis, NOS production, and glycolysis (Fig. 6g). Collectively, our results demonstrate that PARP-1-mediated ADPRylation of STAT1α is a critical regulator of inflammatory responses in macrophages and may suggest a role for PARP inhibitors as a therapeutic tool to control ‘cytokine storms.’

### Roles for PARPs and ADPRylation in inflammation and macrophage biology

A growing list of studies have identified roles for PARPs and ADPRylation in regulating pro-inflammatory responses in macrophages, including NF-κB- and STAT1α-dependent gene expression^4,9^. Using PARP1-deficient mice, Oliver et al. provided the first evidence that PARP-1 promotes NF-κB activation in macrophages and is required for inflammatory responses in vivo^18^. Other studies have focused on the role of PARP-1 in modulating NF-κB-dependent gene regulation^19,45–47^, which may occur independent of PARP-1 catalytic activity^21,45,47^. Recent studies have shown that two cytosolic PARP family members, PARP-9 and PARP-14, have opposing roles in macrophage activation^48^. PARP14-mediated monoADPRylation of cytosolic STAT1α inhibits pro-inflammatory gene expression and STAT1α phosphorylation, whereas PARP-9, which is thought to be a catalytically inactive PARP family member^49^, counteracts these effects^48^.

Our observations with PARP-1, which are in the context of nuclear STAT1α, provide a direct understanding of how its transcriptional functions are regulated by ADPRylation. Importantly, the sites of polyADPRylation that we have identified do not overlap with sites of monoADPRylation catalyzed by PARP-14^48^. Moreover, PARP-14-dependent ADPRylation of STAT1α leads to a decrease in Y701 phosphorylation^48^, which is in direct contrast to our observations made in the context of S727, as well as Y701, phosphorylation events. These data suggest distinct regulatory pathways and mechanisms are involved in PARP-1-mediated regulation of STAT1α activity through site-specific ADPRylation. Collectively, our results, taken together with the results from the previous studies, demonstrate the importance of ADPRylation mediated by various PARP family members in regulating STAT1α activity in different cellular compartments, while highlighting the distinct roles of these PARPs in modulating the activity of a single target.

Our results on the biology of PARP-1 and ADPRylation in macrophages fit well with the growing recognition of specific functional relationships between PARP-1 and the nuclear NAD^+^ synthesis pathway^50,51^, and the importance of NAD^+^ as a critical regulator of pro-inflammatory responses in macrophages^40,41^. Importantly, reductions in cellular NAD^+^ levels are known to impair the induction of glycolysis in pro-inflammatory macrophages^40^. Our work provides a mechanistic perspective for how this metabolite, when used as a substrate for PARP-1-mediated ADPRylation, can regulate the activity of a modulator of immune responses, such as STAT1α. Indeed, a reduction in STAT1α ADPRylation exhibited similar outcomes as a reduction in NAD^+^, the substrate for ADPRylation (Fig. 4h, i). Thus, we conclude that the NAD^+^-dependent macrophage responses, such as induction of glycolysis, are mediated in part by site-specific ADPRylation of STAT1α. These observations provide insights into the mechanisms of metabolic regulation of immune responses in macrophages.

### Multiple site-specific ADPRylation events on STAT1α control distinct molecular and cellular outcomes

Although a number of recent studies have identified a diverse array of proteins as substrates for ADPRylation^15,43^, most of these studies have focused on the biochemical effects of ADPRylation and have not typically explored the physiological impact of site-specific ADPRylation. In addition, while many studies explore the overall impact of ADPRylation on protein function, few identify specific sites of ADPRylation and assess their function in detail through mutagenesis. The latter is essential for understanding the specific effects of ADPRylation and the mechanisms through which it acts. In our work, we have found that ADPRylation can have a significant impact on IFNγ-stimulated macrophage activity. More importantly, we have shown that site-specific ADPRylation of STAT1α is critical for mediating these IFNγ-dependent pro-inflammatory responses. Moreover, we present the first observations showing that ADPRylation at different sites on the same protein can have dramatically different mechanistic consequences.

Although ADPRylation, especially PARylation, is frequently portrayed as a non-specific PTM that can modify protein activity simply by introducing a highly negatively charged moiety, our results indicate that it can have more specific and precise effects. Depending on the site that is modified, ADPRylation, and hence PARP-1, can differentially regulate distinct functions of the same transcription factor (e.g., DNA binding, transcriptional activation). Whether ADPRylation as these distinct sites occurs simultaneously or sequentially remains to be determined. An intriguing possibility is that the presence of one site-specific ADPRylation event is necessary to drive a second one. Future studies focusing on the interplay between the distinct ADPRylation sites will elucidate how one enzyme, PARP-1, can selectively modulate diverse target activities.

### Functional interplay between site-specific ADPRylation, phosphorylation, and p300 activation in the regulation of STAT1α enhancers

Sites of ADPRylation are enriched near sites of phosphorylation across the human proteome^43,52^. While a previous study has shown that ADPRylation and phosphorylation of the same Ser residue in a core histone (i.e., H3-Ser10) are incompatible^53^, our results demonstrate that ADPRylation of a protein at one amino acid can be required for its phosphorylation at another amino acid. Mechanistically, how the presence of ADPRylation impacts phosphorylation on STAT1α is not clear, but may involve creation of a recognition site for the kinase or activate the catalytic activity of the kinase. STAT1α phosphorylation at S727 is thought to be mediated by MAPK and CDK8^54,55^. ADPRylation at D721 of STAT1α may directly facilitate their recruitment or activation, a possibility that will be explored in future studies. A number of proteins have specific ADPRylation reader domains that mediate their interactions with other proteins^56^. This may also represent a potential mechanism for the interplay between ADPRylation and phosphorylation on STAT1α.

An interesting facet of our work was the observation that ADPRylation of STAT1α is required to activate p300. The STAT1α TA domain has been shown to be required for the recruitment of p300 to STAT1α enhancers and the stimulation of its catalytic activity^33,44^. Intriguingly, we observed that p300 activation and subsequent histone acetylation at STAT1α enhancers is attenuated in the absence of TA domain ADPRylation, perhaps through the inhibition of Ser727 phosphorylation. These results reveal how ADPRylation can impact transcription by influencing other PTMs that activate transcription factors and their associated coregulators. Furthermore, understanding the relationship between two distinct ADPRylation-dependent events at STAT1α enhancers (i.e., phosphorylation and p300 activation) will provide insight into the regulation of enhancer activation by PARP-1.

## Methods

### Antibodies

The following antibodies were used for immunoblotting and immunofluorescent staining: STAT1 rabbit polyclonal antibody (Cell Signaling, 9172 L); Phospho-STAT1α (Ser727) rabbit monoclonal antibody (Cell Signaling, 8826 S); Phospho-STAT1α (Ser727) rabbit polyclonal antibody (Cell Signaling Technologies, 9177); Phospho-STAT1α (Tyr701) rabbit polyclonal antibody (Cell Signaling Technologies, 9167); Flag mouse monoclonal antibody (Sigma-Aldrich, F3165); β-tubulin rabbit polyclonal antibody (Abcam, ab6046); p300 mouse monoclonal antibody (Active motif, 61401); Acetyl-CBP (Lys1535)/p300 (Lys1499) rabbit polyclonal antibody (Cell Signaling, 4771); rabbit IgG (ThermoFisher Scientific, 10500 C); goat anti-rabbit HRP-conjugated IgG (Pierce, 31460); and goat anti-mouse HRP-conjugated IgG (Pierce, 31430). The custom rabbit polyclonal antiserum against PARP-1 used for immunoblotting was generated by using an antigen comprising the amino-terminal half of PARP-1^57^ (now available from Active Motif; cat. no. 39559). The custom recombinant antibody-like anti-ADP-ribose binding reagent were generated and purified in-house^58^ (now available from EMD Millipore; cat. no. MABE1031, MABE1016). STAT1 (Santa Cruz Biotech, sc-592) and Histone H3 (acetyl K27) (Abcam, ab4729) rabbit polyclonal antibodies were used for chromatin immunoprecipitation assays. STAT1 rabbit polyclonal antibody (Cell Signaling, 9172 L), Phospho-STAT1α (Ser727) rabbit polyclonal antibody (Cell Signaling Technologies, 9177), Alexa Fluor 594 donkey anti-rabbit IgG (ThermoFisher, A-21207) were used for immunofluorescence.

### Cell Culture

Mouse immortalized bone marrow-derived macrophages (iBMDMs) were a gift from Dr. Inez Rogatsky (Hospital for Special Surgery, New York). L-929, 293 T, MCF-7 and THP-1 cells were purchased from the American Type Culture Collection (ATCC). iBMDMs were cultured in low glucose DMEM (Sigma-Aldrich, D6046) supplemented with 10% fetal bovine serum and 1% penicillin/streptomycin. 293 T cells were cultured in high glucose DMEM (Sigma-Aldrich, D5796) supplemented with 10% fetal bovine serum and 1% penicillin/streptomycin. THP-1 cells were cultured in RPMI (Sigma-Aldrich, R8758) supplemented with 10% fetal bovine serum and 1% penicillin/streptomycin. 2-mercaptoethanol (Sigma-Aldrich, 63689) was added to the RPMI at a final concentration of 0.34% v/v. THP-1 cells were differentiated using 25 ng/mL of PMA (Sigma-Aldrich, P1585) for 72 h. MCF-7 cells were maintained in Minimum Essential Medium (MEM) Eagle supplemented with 5% calf serum. Sf9 insect cells were cultured in SF-II 900 medium (Invitrogen, 10902096). Fresh cell stocks of all cell lines were replenished after 5 passages. All cell lines were tested and verified as mycoplasma-free every 6 months.

### Generation of bone marrow-derived macrophages (BMDMs)

We collected and cultured primary BMDMs as described below for use in a variety of experiments.

#### Mice used for generating primary BMDMs

All animal experiments were performed according to procedures approved by the UTSW Institutional Animal Care and Use Committee and complied with the ethical regulations for animal testing and research. Mice were maintained on a standard rodent chow diet with 12-hour light/12-hour dark cycles in a temperature-controlled environment (room temperature, 22 °C; thermoneutrality, 30 °C). C57BL/6 mice were obtained from the Mouse Breeding Core at UT Southwestern. *Parp1* null (*Parp1*^*-/-*^) mice on a C57BL/6 background were described previously^59^.

#### Production of L-929 cell conditioned medium (LCCM)

L-929 fibroblast cells were grown to confluence in T150 flasks in low glucose DMEM (Sigma-Aldrich, D6046) supplemented with 10% fetal bovine serum. Once confluent, 30 mL of fresh medium was added to the cells and they were cultured for 10 additional days. On day 10, the cell medium was collected, filtered, and stored at 4 °C to be used as 10x LCCM.

#### Culture of primary mouse BMDMs

BMDMs were harvested from age- and sex- matched eight- to twelve-week-old C57BL/6 mice as previously described^60^. For the bone marrow extraction, femurs and tibias from the hind legs of the mice were used. Prior to bone marrow collection, the mice were euthanized using CO_2_ per IACUC standards. The bones were removed, cleaned to remove skin, muscle and cartilage, and flushed with low glucose DMEM supplemented with 20% fetal bovine serum and 10% (LCCM) under sterile conditions to collect the bone marrow. The bone marrow-derived cells were suspended in 50 mL of the same medium and seeded in non-treated 100 mm diameter plates (Corning, 08-757-100D). The cells were incubated for 5 days to allow the differentiation for the precursors into macrophages. On day 6, the macrophages were scraped and seeded as needed for the experiments. The cells were treated the following day.

### Cell treatments

Cells were treated with 100 ng/mL of murine IFNγ (Peprotech, 315-05) for 1 h for molecular assays or longer for Seahorse and NOS activity assays (as described below). For PJ34 treatment, the cells were pre-treated with 20 µM PJ34 (Abcam, ab120981) for 1 h (BMDM, THP1) or 2 h (iBMDM) prior to stimulation. For veliparib treatment, iBMDMs were treated with 10 µM veliparib (MedChemExpress, HY-10129) for 2 h prior to stimulation. For doxycycline (Dox) induction, cells were treated with 1 μg/mL of Dox (Sigma-Aldrich, D9891) for 24 h prior to additional treatments.

### Molecular cloning to generate knockdown and expression vectors

We used standard molecular cloning techniques to generate the following vectors for expressing or depleting proteins of interest.

#### STAT1α expression constructs

cDNA pools were prepared by extraction of total RNA from MCF-7 cells (human) or iBMDM cells (mouse) using the RNeasy Plus Kit (Qiagen, 74134), followed by reverse transcription using superscript III Reverse Transcriptase (Invitrogen, 18080093) with random hexamer primers (Roche, 11034731001) according to the manufacturer’s instructions. The cDNA pools were used to amplify STAT1α cDNAs for subsequent cloning. cDNAs encoding N-terminally Flag epitope-tagged human and mouse wild-type (Wt) STAT1α were cloned into *BamHI-* and *NotI*-digested pcDNA3 using the primers listed below. ADPRylation site point mutants for STAT1α were generated by site-directed mutagenesis in the pCDNA3-Flag-STAT1α vectors using Pfu Turbo DNA polymerase (Agilent, 600250) with primers listed below. The STAT1α DBD mutant was generated by mutating the glutamates at positions 393/394 to glutamines and the TA mutant was generated by changing the aspartate residue at position 721 to asparagine. Flag epitope-tagged human STAT1α S727A was cloned into pCDNA3 from the eGFP STAT1 S727A plasmid. The eGFP STAT1 S727A was kindly provided by Alan Perantoni (Addgene plasmid #12304; http://n2t.net/addgene:12304; RRID:Addgene_12304)^61^.

#### Inducible expression constructs

To generate Dox-inducible lentiviral vectors for expression of mouse wild-type or mutant STAT1α and eGFP, the respective Flag epitope-tagged cDNAs were amplified from the pcDNA3 expression vectors (described above). The cDNAs were cloned into *NheI-* and *XhoI*- digested pINDUCER20 (Addgene, plasmid no. 44012) using a Gibson Assembly kit (NEB, E2621).

#### Insect cell expression vectors

The human STAT1α cDNA was amplified by PCR from pcDNA3-Flag-STAT1α (described above) and then cloned into *NotI*- and *BamHI-*digested pFastBac. The pFastBac-Flag-PARP-1 was generated by Gibson et al.^43^. Recombinant bacmids were generated by transforming the pFastBac-Flag-STAT1α and pFastBac-Flag-PARP-1 vectors into DH10BAC *E. coli* with subsequent blue/white colony screening using the Bac-to-Bac system (Invitrogen) according to the manufacturer’s instructions.

#### shRNAs targeting the Stat1 and Parp1 mRNAs

shRNA constructs targeting the 3′ UTR of mouse *Stat1* mRNA (TRCN0000235837) and control shRNA (SHC002) were purchased from Sigma. The shRNA construct targeting mouse *Parp1* mRNA expressed from the pLKO.1 vector (SHC001), which confers puromycin resistance, has been previously described^50^.

### Oligonucleotide primers used for molecular cloning

Oligonucleotide primers used for molecular cloning are listed in Supplementary Table 1.

### Generation of cell lines with stable knockdown or ectopic expression

iBMDM cells were transduced with lentiviruses for stable knockdown or ectopic expression. We generated lentiviruses by transfection of the pLKO.1 and pINDUCER20 constructs described above, together with: (i) an expression vector for the VSV-G envelope protein (pCMV-VSV-G, Addgene plasmid no. 8454); (ii) an expression vector for GAG-Pol-Rev (psPAX2, Addgene plasmid no. 12260); and (iii) a vector to aid with translation initiation (pAdVAntage, Promega) into 293 T cells using Lipofectamine 3000 Reagent (Invitrogen, L3000015) according to the manufacturer’s protocol. The resulting viruses were collected in the culture medium, concentrated by using a Lenti-X concentrator (Clontech, 631231), and used to infect iBMDM cells seeded at a density of 1 × 10^6^. Stably transduced cells were selected with puromycin (Sigma, P9620; 2.5 μg/mL) or G418 sulfate (Sigma, A1720; 1 mg/mL) in cell culture medium.

### Expression and purification of recombinant proteins

We used the following protocols to express and purify STAT1α, PARP-1, and p300 for use in biochemical assays.

### Purification of STAT1α expressed in mammalian cells

293 T cells were seeded at ~2 × 10^6^ cells per 15 cm diameter dish and transfected at ~60% confluence with pcDNA3 containing a cDNA encoding Flag-tagged wild-type or mutant (DBD or TA) human STAT1α (described above) using Lipofectamine 3000 Reagent (Invitrogen, L3000015) for 48 h according to the manufacturer’s protocol. The cells were collected in ice cold PBS and collected by centrifugation in a microfuge at 1,000 RCF for 5 min at 4 °C. The cells were then resuspended in ice cold Lysis Buffer (50 mM Tris-HCl pH 7.4, 150 mM NaCl, 1 mM EDTA, and 0.5% NP-40) supplemented with phosphatase inhibitors (1 mM sodium fluoride and 1 mM sodium orthovanadate), a PARG inhibitor to prevent PAR chain cleavage during extraction (250 nM ADP-HPD; Sigma-Aldrich, A0627;) and 1x complete protease inhibitor cocktail (Roche, 11697498001), and incubated for 30 min at 4 °C to produce a whole cell lysate.

For purifying recombinant proteins from nuclear extracts, the cell pellets were resuspended in Isotonic Buffer (10 mM Tris-HCl pH 7.5, 2 mM MgCl_2_, 3 mM CaCl_2_, 0.3 M sucrose) supplemented with phosphatase inhibitors (1 mM sodium fluoride and 1 mM sodium orthovanadate), 1x phosphatase inhibitor cocktail (Sigma-Aldrich, P0044, P5726), and 1x complete protease inhibitor cocktail (Roche, 11697498001), incubated on ice for 15 min, and lysed by the addition of 0.6% NP-40 detergent with gentle vortexing. The nuclei from the lysed cells were collected by centrifugation in a microfuge at 11,000 RCF for 30 s at 4 °C. The pelleted nuclei were resuspended in Nuclear Extraction Buffer (50 mM Tris-HCl pH 7.5, 150 mM NaCl, 1 mM EDTA, 1% NP-40) supplemented with the phosphatase and protease inhibitors described above and extracted on ice for 30 min to produce the nuclear extract.

The resulting whole cell and nuclear extracts were clarified by two rounds of centrifugation at 21,000 RCF in a microfuge for 10 min at 4 °C and then incubated with pre-equilibrated anti-Flag M2 beads (Sigma-Aldrich, A2220) for 4 h at 4 °C with gentle mixing. The resin was washed five times with gentle mixing for 10 min at 4 °C with Immunoaffinity Purification Wash Buffer (25 mM Tris-HCl pH 7.5, 450 mM NaCl, 1% NP-40, 1 mM EDTA) containing 1 mM sodium fluoride, 1 mM sodium orthovanadate, and 1x complete protease inhibitor cocktail. The STAT1α proteins were eluted from the agarose resin by the addition of Immunoaffinity Purification Elution Buffer (25 mM Tris-HCl pH 7.5, 200 mM NaCl, 0.25% NP-40, 10% glycerol, 1 mM EDTA) containing 1 mM sodium fluoride, 1 mM sodium orthovanadate, 1x complete protease inhibitor cocktail, 250 nM APD-HPD, and 0.2 mg/mL of 3x Flag peptide (Sigma-Aldrich, F4799). Purified STAT1α was aliquoted, flash frozen in liquid N_2_, and stored at -80 °C until further use. The concentration of the eluted proteins was determined by comparing to BSA standards using SDS-PAGE with subsequent silver staining using a Pierce silver staining kit (ThermoFisher, 24600) following the manufacturer’s protocol.

#### Purification of STAT1α and PARP-1 expressed in Sf9 insect cells

Sf9 insect cells, cultured in SF-II 900 medium (Invitrogen, 10902096), were transfected with 1 μg of bacmid driving expression of Flag-tagged STAT1α or Flag-tagged PARP-1 using Cellfectin transfection reagent (Invitrogen, 10362100) according to the manufacturer’s protocol. After five hours, the medium was supplemented with 10% FBS, penicillin, and streptomycin, and the cells were incubated for three days. The culture medium was collected as a baculovirus stock after 72 h. After three rounds of amplification of the stock, the resulting high titer baculovirus was used to infect fresh Sf9 cells to induce protein expression. After 48 h of infection, the cells were collected by centrifugation. The cells were resuspended in Flag Lysis Buffer (20 mM HEPES pH 7.9, 0.5 M NaCl, 4 mM MgCl_2_, 0.4 mM EDTA, 20% glycerol, 250 mM nicotinamide, 2 mM β-mercaptoethanol) containing 2 mM sodium fluoride, 2 mM sodium orthovanadate, and 2x protease inhibitor cocktail and then lysed by Dounce homogenization and sonication. The lysate was clarified by centrifugation at 26,800 RCF for 30 min at 4 °C in a Sorvall centrifuge, transferred to a fresh tube, and mixed with an equal volume of Flag Dilution Buffer (20 mM HEPES pH 7.9, 10% glycerol, 0.02% NP-40). The diluted lysate was mixed with anti-Flag M2 agarose resin and incubated for 3 h at 4 °C with gentle mixing.

After incubation, the resin was washed as follows: (1) twice with Flag Wash Buffer #1 (20 mM HEPES pH 7.9, 150 mM NaCl, 2 mM MgCl_2_, 0.2 mM EDTA, 15% glycerol, 0.01% NP-40, 0.2 mM β-mercaptoethanol) containing with 100 mM nicotinamide, 1 mM PMSF, 1 μM aprotinin, 100 μM leupeptin, 1 mM sodium fluoride, and 1 mM sodium orthovanadate, (2) twice with Flag Wash Buffer #2 [20 mM HEPES pH 7.9, 1 M NaCl (for PARP-1) or 0.5 M NaCl (for STAT1α), 2 mM MgCl_2_, 0.2 mM EDTA, 15% glycerol, 0.01% NP-40, 0.2 mM β-mercaptoethanol] containing100 mM nicotinamide, 1 mM PMSF, 1 μM aprotinin, 100 μM leupeptin, 1 mM sodium fluoride, 1 mM sodium orthovanadate, and (3) twice with Flag Wash Buffer #3 (20 mM HEPES pH 7.9, 200 mM NaCl, 2 mM MgCl_2_, 0.2 mM EDTA, 15% glycerol, 0.01% NP-40, 0.2 mM β-mercaptoethanol, 1 mM PMSF). The Flag-tagged PARP-1and STAT1α proteins were eluted from the anti-Flag M2 agarose resin with Flag Wash Buffer #3 containing 0.2 mg/mL 3x Flag peptide, flash frozen in liquid N_2_, and stored at −80 °C.

#### Purification of p300 expressed in Sf9 insect cells

Sf9 cells were infected with baculovirus driving the expression of Flag-tagged p300 for 48 h at the Protein and Monoclonal Antibody Production Shared Resource at Baylor College of Medicine. The Sf9 cells were treated for 3 h prior to harvesting with the following p300 inhibitors: 10 μM SGC-CBP30 (Sigma-Aldrich, SML1133), 25 μM C646 (Sigma-Aldrich, SML0002), and 10 μM of A-485 (Tocris, 6387). After 48 h of incubation, the cells were collected by centrifugation. The cells were resuspended in Flag-p300 Lysis Buffer (20 mM Tris-HCl pH 7.9, 0.5 M NaCl, 4 mM MgCl_2_, 5 μM ZnCl_2_, 20% glycerol, 2 mM β-mercaptoethanol, 2x protease inhibitor cocktail) and lysed by Dounce homogenization and sonication. The lysate was clarified by centrifugation in a Sorvall centrifuge 26,800 RCF for 30 min at 4 °C and mixed with an equal volume of Flag-p300 Dilution Buffer (20 mM Tris-HCl pH 7.9, 10% glycerol, 0.02% NP-40, 5 μM ZnCl_2_). The diluted lysate was incubated for 3 h with anti-Flag M2 agarose resin and washed five times with Flag-p300 Wash Buffer (20 mM Tris-HCl pH 7.9, 200 mM NaCl, 2 mM MgCl_2_, 5 μM ZnCl_2_, 15% glycerol, 0.1% NP-40, 0.2 mM β-mercaptoethanol, 1 mM PMSF, 1 μM aprotinin, 100 μM leupeptin). The Flag-tagged p300 protein was eluted from the anti-Flag M2 agarose resin with Flag-p300 elution buffer (20 mM Tris-HCl pH 7.9, 100 mM NaCl, 2 mM MgCl_2_, 5 μM ZnCl_2_, 15% glycerol, 0.01% NP-40, 0.2 mM β-mercaptoethanol, 1 mM PMSF) containing 0.2 mg/mL 3x Flag peptide, flash frozen in liquid N_2_, and stored at −80 °C.

### Preparation of Cell Lysates and Immunoblotting

#### Culturing cells for lysate preparation

293 T cells were seeded at ~2 × 0^6^ cells per 15 cm diameter plate and transfected at ~60% confluence with pcDNA3 containing a cDNA encoding Flag-tagged wild-type or mutant (DBD or TA) human STAT1α as described above using Lipofectamine 3000 Reagent (Invitrogen, L3000015) for 48 h according to the manufacturer’s protocol. iBMDM cells ectopically expressing Flag-tagged wild-type or mutant (DBD or TA) mouse STAT1α were seeded in 10 cm diameter plates at a density of ~5 × 10^6^ and the protein expression was induced by treating with Dox for 24 h as described above. BMDM were seeded in 10 cm diameter plates at a density of ~1 × 10^6^. THP1 cells were seeded at a density of 1 × 10^6^ cells in 10 cm diameter plates. All the cells were cultured and treated as described above. The cells were then washed, collected with ice cold PBS, and pelleted by centrifuging at 1000 RCF.

#### Preparation of whole cell lysates

The cell pellets were lysed with Cell Lysis Buffer (20 mM Tris-HCl pH 7.5, 150 mM NaCl, 1 mM EDTA, 1 mM EGTA, 1% NP-40, 1% sodium deoxycholate, 0.1% SDS) containing: 1 mM sodium fluoride and 1 mM sodium orthovanadate (phosphatase inhibitors), 250 nM ADP-HPD (Sigma, A0627; a PARG inhibitor to prevent PAR chain cleavage during extraction), 20 μM PJ34 (a PARP inhibitor to prevent PAR synthesis during extraction), 1x phosphatase inhibitor cocktail (Sigma-Aldrich, P0044, P5726) and 1x complete protease inhibitor cocktail (Roche, 11697498001. The lysates were incubated on ice for 30 min with gentle mixing and clarified by centrifugation at 21,000 RCF in a microfuge for 15 min at 4 °C.

#### Preparation of nuclear and cytosolic extracts

The cell pellets were resuspended in Isotonic Buffer (10 mM Tris-HCl pH 7.5, 2 mM MgCl2, 3 mM CaCl2, 0.3 M sucrose, 1 mM sodium fluoride, 1 mM sodium orthovanadate, 1x phosphatase inhibitor cocktail, and 1x complete protease inhibitor cocktail), incubated on ice for 15 min, and lysed by the addition of 0.6% NP-40 detergent with gentle vortexing. The nuclei from the lysed cells were collected by centrifugation in a microfuge at 11,000 RCF for 30 s and the supernatant was collected as the cytoplasmic fraction. The pelleted nuclei were resuspended in Nuclear Extraction Buffer (50 mM Tris-HCl pH 7.5, 150 mM NaCl, 1 mM EDTA, 1% NP-40, 1 mM sodium fluoride, 1 mM sodium orthovanadate, 1x phosphatase inhibitor cocktail, and 1x complete protease inhibitor cocktail) to produce the nuclear lysate. The lysates were incubated on ice for 30 min for extraction and then centrifuged twice at 21,000 RCF in a microfuge for 15 min per run at 4 °C.

#### Determination of protein concentrations and immunoblotting

Protein concentrations in the lysates were determined using Bradford reagent (Bio-Rad, 50000006). The lysates were run on a 7% polyacrylamide-SDS gel (for ADPRylation analyses) or an 8% polyacrylamide-SDS gel (for PARP-1, STAT1α,and β-tubulin), and transferred to a nitrocellulose membrane. The membranes were blocked with 5% nonfat milk in TBST and incubated with the primary antibodies described above in 1% nonfat milk made in TBST or 5% BSA (for phosphorylation blots), followed by anti-rabbit HRP-conjugated IgG (1:5000) or anti-mouse HRP-conjugated IgG (1:5000). Western blot signals were detected using an ECL detection reagent (ThermoFisher, 34077, 34095).

### Immunoprecipitation of nuclear proteins

293 T cells were seeded at ~2 × 10^6^ cells per 15 cm diameter plate and transfected at ~60% confluence with pcDNA3 containing a cDNA encoding Flag-tagged wild-type or mutant (DBD or TA) human STAT1α as described above using Lipofectamine 3000 Reagent (Invitrogen, L3000015) for 48 h according to the manufacturer’s protocol. iBMDM cells ectopically expressing Flag-tagged wild-type or mutant (DBD or TA) mouse STAT1α were seeded in 15 cm diameter plates at a density of ~10 × 10^6^ and the protein expression was induced by treating with Dox for 24 h as described above. The cells were collected and nuclear extract were prepared as described above. The resulting extracts were incubated with equilibrated anti-M2-Flag beads (Sigma-Aldrich, A2220) for 16 h at 4 °C with gentle mixing. The beads were washed five times with gentle mixing for 10 min at 4 °C with Immunoaffinity Purification Wash Buffer (25 mM Tris-HCl pH 7.5, 450 mM NaCl, 1% NP-40, 1 mM EDTA, 1 mM sodium fluoride, 1 mM sodium orthovanadate, and 1x complete protease inhibitor cocktail). The beads were then heated to 100 °C for 5 min in 2x SDS-PAGE loading buffer to release the bound proteins. The immunoprecipitated material was subjected to immunoblotting as described above. All immunoblots for STAT1α were probed between the regions of 75 and 100 kDa.

### *In nuclei* ADPRylation assays

*In nuclei* ADPRylation assays were carried out as described previously^43^. iBMDM cells ectopically expressing Flag-tagged wild-type or mutant (DBD or TA) mouse STAT1α were cultured and treated with PDD00017273 PARG inhibitor (Fisher, 590521-0) harvested in ice cold PBS and collected by centrifugation. The cells pellets were resuspended in Isotonic Buffer (10 mM Tris-HCl pH 7.5, 2 mM MgCl2, 3 mM CaCl2, 0.3 M sucrose, 1 mM sodium fluoride, 1 mM sodium orthovanadate, 1x phosphatase inhibitor cocktail, and 1x complete protease inhibitor cocktail), incubated on ice for 15 min, and lysed by the addition of 0.6% NP-40 detergent with gentle vortexing. The nuclei were collected by centrifugation in a microfuge at 11,000 RCF for 30 sec at 4 °C and then resuspended in ADPRylation Reaction Buffer (30 mM Tris-HCl, pH 7.5, 10 mM KCl, 5 mM MgCl_2_, 5 mM CaCl_2_, 0.01% NP-40, 0.05 mM EDTA, 20% glycerol, with freshly added 1 mM DTT, 1 mM sodium fluoride, 1 mM sodium orthovanadate, and 1x complete protease inhibitor cocktail) containing 250 μM NAD^+^ for 30 min at room temperature with occasional gentle mixing to allow ADPRylation to occur. The nuclei were then centrifuged for 1 min at 2,000 RCF and resuspended in the Nuclear Extraction Buffer (described above). Subsequent Flag-epitope-based immunoprecipitation was carried out from the extracts as described above. All immunoblots for STAT1α ADPRylation were probed between the regions of 75 and 100 kDa.

### In vitro ADPRylation assays

In vitro ADPRylation assays were performed essentially as described previously^51,62^. To monitor PARP-1-dependent STAT1α ADPRylation, 200 ng of purified recombinant PARP-1 protein was incubated with 2 µg of purified recombinant wild-type STAT1α in ADPRylation Buffer (50 mM Tris-HCl pH 7.5, 12.5 mM MgCl_2_, 125 mM NaCl). The reaction was initiated by the addition of 30 ng/μL sonicated salmon sperm DNA (ThermoFisher, AM9680) and 100 µM NAD^+^ at room temperature for 20 min. The ADPRylation reactions were stopped by the addition of 4x SDS-PAGE loading buffer with subsequent heating at 100 °C for 10 min. To detect ADPRylation, the reaction mixes were resolved on an 8% PAGE-SDS gel, transferred to a nitrocellulose membrane, and subjected to immunoblotting with an ADP-ribose detection reagent (MABE1016, EMD Millipore) as described above.

### In vitro acetylation assays

In vitro acetylation assays were performed as described previously^63^. Wild-type or mutant STAT1α proteins were in vitro ADPRylated as described above. The ADPRylation reaction was stopped by adding 20 µM PJ34. To monitor the effect of ADPRylated STAT1α on p300 auto-acetylation, 150 ng of purified recombinant p300 protein was incubated with 30 ng of ADPRylated wild-type or mutant STAT1α in Acetylation Buffer (25 mM Tris-HCl pH 7.5, 100 mM NaCl, 10% glycerol) supplemented with 5 µM Acetyl Co-A, for 30 min at 30 °C. The acetylation reactions were stopped by the addition of 4x SDS-PAGE loading buffer with subsequent to heating at 65 °C for 10 min. To detect the p300 auto-acetylation, the reactions mixes were resolved on 7% PAGE-SDS gel, transferred to a nitrocellulose membrane, and subjected to immunoblotting with an antibody that detects acetylated p300.

### Identification of the sites of ADPRylation on STAT1α

We used the following protocols to determine the sites of ADPRylation on STAT1α by mass spectrometry.

#### Immunoprecipitation of Flag-epitope tagged STAT1α

293 T cells were seeded in five 15 cm diameter plates at ~2 × 10^6^ cells per plate and transfected at ~60% confluence with pcDNA3 containing a cDNA encoding Flag-tagged wild-type human STAT1α, as described above, using Lipofectamine 3000 Reagent (Invitrogen, L3000015) for 48 h according to the manufacturer’s specifications. The cells were collected in ice cold PBS and pelleted by centrifugation in a microfuge at 1,000 RCF for 5 min at 4 °C. The cell pellets were resuspended in Isotonic Buffer (10 mM Tris-HCl pH 7.5, 2 mM MgCl_2_, 3 mM CaCl_2_, 0.3 M sucrose, 1 mM sodium fluoride, 1 mM sodium orthovanadate, 1x phosphatase inhibitor cocktail, and 1x complete protease inhibitor cocktail), incubated on ice for 15 min, and lysed by the addition of 0.6% NP-40 detergent with gentle vortexing. The nuclei from the lysed cells were pelleted by centrifugation in a microfuge at 11,000 RCF for 30 s. The pelleted nuclei were resuspended in Nuclear Extraction Buffer (50 mM Tris-HCl pH 7.5, 150 mM NaCl, 1 mM EDTA, 1% NP-40, 1 mM sodium fluoride, 1 mM sodium orthovanadate, 1x phosphatase inhibitor cocktail, and 1x complete protease inhibitor cocktail) to produce the nuclear lysate.

The resulting extracts were clarified by two rounds of centrifugation at full speed in a microfuge for 10 min at 4 °C and then incubated with equilibrated anti-M2-Flag beads (Sigma-Aldrich, A2220) for 4 h at 4 °C with gentle mixing. The beads were washed seven times with Immunoaffinity Purification Wash Buffer (25 mM Tris-HCl pH 7.5, 450 mM NaCl, 1% NP-40, 1 mM EDTA, 1 mM sodium fluoride, 1 mM sodium orthovanadate, and 1x complete protease inhibitor cocktail) with gentle mixing for 10 min at 4 °C. The washed beads were incubated at 4 °C for 12 h in 0.5 M hydroxylamine in 100 mM HEPES (pH 8.5) with gentle mixing and re-washed as before. The beads were then heated to 100 °C for 5 min in 2x SDS-PAGE loading buffer to release the bound STAT1α protein.

#### Immunoprecipitation of endogenous STAT1α

iBMDM cells were seeded in 60 × 15 cm diameter plates at ~10 × 10^6^ cells per plate and treated with IFNγ for 1 h. The cells were collected in ice cold PBS and pelleted by centrifugation in a microfuge at 1,000 RCF for 5 min at 4 °C. The cell pellets were resuspended in Isotonic Buffer (10 mM Tris-HCl pH 7.5, 2 mM MgCl_2_, 3 mM CaCl_2_, 0.3 M sucrose, 1 mM sodium fluoride, 1 mM sodium orthovanadate, 1x phosphatase inhibitor cocktail, and 1x complete protease inhibitor cocktail), incubated on ice for 15 min, and lysed by the addition of 0.6% NP-40 detergent with gentle vortexing. The nuclei from the lysed cells were pelleted by centrifugation in a microfuge at 11,000 RCF for 30 s. The pelleted nuclei were resuspended in Nuclear Extraction Buffer (50 mM Tris-HCl pH 7.5, 150 mM NaCl, 1 mM EDTA, 1% NP-40, 1 mM sodium fluoride, 1 mM sodium orthovanadate, 1x phosphatase inhibitor cocktail, and 1x complete protease inhibitor cocktail) to produce the nuclear lysate.

The resulting extracts were clarified by two rounds of centrifugation at full speed in a microfuge for 10 min at 4 °C, pre-cleared for 1 h with 30 µg rabbit IgG and then incubated with anti-STAT1 antibody for 12 h at 4 °C with gentle mixing. Protein A agarose beads (Thermo Scientific, 20333) were then added to the lysates and gently mixed at 4 °C for 3 h. The beads were washed seven times with Immunoaffinity Purification Wash Buffer (25 mM Tris-HCl pH 7.5, 450 mM NaCl, 1% NP-40, 1 mM EDTA, 1 mM sodium fluoride, 1 mM sodium orthovanadate, and 1x complete protease inhibitor cocktail) with gentle mixing for 10 min at 4 °C. The washed beads were incubated at 4 °C for 12 h in 0.5 M hydroxylamine in 100 mM HEPES (pH 8.5) with gentle mixing and re-washed as before. The beads were then heated to 100 °C for 5 min in 2x SDS-PAGE loading buffer to release the bound STAT1α protein.

#### LC–MS/MS analysis

Eluted STAT1α protein was run on a 4–12% acrylamide-SDS gel (Invitrogen, NW04120BOX) and visualized by Coomassie blue staining. Gels slices containing the STAT1α protein were excised and transferred to a microfuge tube. Following reduction and alkylation with DTT and iodoacetamide (Sigma-Aldrich, A3221), respectively, the STAT1α protein in the Gels slices was digested overnight with trypsin (Promega, V5111). The samples were then subjected to solid-phase extraction cleanup with an Oasis HLB plate (Waters) and the resulting samples were injected onto an Orbitrap Fusion Lumos mass spectrometer (Thermo Electron) coupled to an Ultimate 3000 RSLC-Nano liquid chromatography system (Dionex). The samples were injected onto a 75 μm i.d., 50-cm long EasySpray column (Thermo) and eluted with a gradient from 1 to 28% Buffer B over 60 min. Buffer A contained 2% (v/v) ACN and 0.1% formic acid in water, and Buffer B contained 80% (v/v) ACN, 10% (v/v) trifluoroethanol, and 0.1% formic acid in water. The mass spectrometer operated in positive ion mode with a source voltage of 1.5–2.4 kV and an ion transfer tube temperature of 275 °C. MS scans were acquired at 120,000 resolution in the Orbitrap and up to 10 MS/MS spectra were obtained in the ion trap for each full spectrum acquired using higher-energy collisional dissociation (HCD) for ions with charges 2–7. Dynamic exclusion was set for 25 s after an ion was selected for fragmentation.

Raw MS data files were converted to a peak list format and analyzed using the central proteomics facilities pipeline (CPFP), version 2.0.3^64,65^. Peptide identification was performed using the X!Tandem (2017.02.01)^66^ and open MS search algorithm (OMSSA)^67^ search engines against the human and mouse protein databases from Uniprot, with common contaminants and reversed decoy sequences appended^68^. Fragment and precursor tolerances of 10 ppm and 0.5 Da were specified, and three missed cleavages were allowed. Carbamidomethylation of cysteine was set as a fixed modification, with oxidation of Methionine and hydroxamic acid modification of Aspartate and Glutamate were set as variable modifications.

### Immunofluorescent staining and confocal microscopy

Mouse BMDM cells were seeded on eight-chambered cover slips (Thermo Fisher, 12-565-2) one day prior to treatment. The following day, cells were treated with IFNγ (with or without PJ34 pre-treatment) for 1 h. The treated cells were washed three times with PBS, fixed in 4% paraformaldehyde for 15 min at room temperature, and washed twice with PBS. The cells were incubated for 30 min at room temperature in Blocking Solution (10% fetal bovine serum, 0.1% Triton X-100, 0.05% sodium azide in PBS). The cells were incubated overnight at 4° with a polyclonal antibody against STAT1 (1:400) or Phospho-STAT1 (Ser727) (1:100) diluted in Blocking Solution. The cells were then washed three times with PBS, incubated with Alexa Fluor 594 donkey anti-rabbit IgG (ThermoFisher, A-21207) in Blocking Solution for 30 min at room temperature, and washed three more times with PBS. Finally, the coverslips were treated with VectaShield (Vector Laboratories, H-1000) and images were acquired using an inverted Zeiss LSM 780 confocal microscope with Zeiss Zen Imaging Software (version 3.3). We used Image J software to subtract background, set thresholds, select the regions of interest (ROIs), and quantify fluorescence intensity in the nuclei. Data were quantified for 3 to 5 fields per treatment from BMDM harvested from 3 different mice per experiment.

### Phagocytosis assays

STAT1-knockdown iBMDM cells harboring expression vectors for Dox-inducible wild-type or ADPRylation site mutant STAT1α were treated with Dox for 24 h to induce protein expression. The cells were collected and resuspended in Opti-MEM medium (Life Technologies, 31985-070), and 1 × 10^5^ cells in 100 μL of medium were seeded in 96-well glass bottom plates (Cellvis, P96-1-N) and allowed to adhere for one hour. iBDMDs were treated with PJ34 for 2 h as indicated. One mg/mL suspensions of pHrodo Green *S. aureus* Bioparticles Conjugates (Thermo Fisher, P35367) were added to the wells according to manufacturer’s instructions. The cells were analyzed for phagocytosis after one hour by live cell imaging using the inverted Zeiss LSM 780 confocal microscope with Zeiss Zen Imaging Software (version 3.3). We used the Cell Counter plugin in Image J software to count the number of cells positive for phagocytosed particles. The data were quantified for 3 biological replicates across 3 fields for each replicate and statistically analyzed using Student’s unpaired t-test.

### NOS activity assays

STAT1-knockdown iBMDM cells ectopically expressing STAT1α wild-type or ADPRylation site mutants were treated with Dox for 24 h to induce protein expression, followed by IFNγ treatment for 24 h. iBDMDs expressing endogenous STAT1α were treated with IFNγ for 24 h in the presence or absence of veliparib. The cells were then harvested and the relative NOS activity was measured using the Nitric Oxide Synthase Activity Assay Kit (Abcam, ab211083) per manufacturer’s instructions. The results were quantified over three biological replicates and significant differences between groups were analyzed using Student’s unpaired t-test.

### Oligonucleotide binding assays

STAT1-knockdown iBMDM cells ectopically expressing STAT1α wild-type or DBD mutant were treated with Dox for 24 h to induce protein expression, followed by IFNγ treatment for 1 h. iBDMDs expressing wild-type STAT1α were treated with PJ34 for 2 h prior to stimulation with IFNγ for 1 h. The cells were harvested and nuclear extracts were prepared as described above. One hundred and twenty five μL of nuclear extract was mixed with 375 μL of Binding Buffer (10 mM Tris-HCl pH 7.5, 50 mM NaCl, 1 mM EDTA, 1 mM DTT, 5% glycerol, 1 μg/mL poly dI-dC, 1 mM sodium fluoride, 1 mM sodium orthovanadate, and 1x phosphatase inhibitor cocktail). One hundred μL of a slurry of STAT3 consensus oligonucleotide agarose conjugates (Santa Cruz Biotech, sc-2571 AC) was added to the extracts and incubated overnight at 4 °C. The beads were then washed three times with Binding Buffer and heated to 100 °C for 5 min in 2x SDS-PAGE loading buffer to release the bound proteins. The immunoprecipitated material was subjected to immunoblotting as described above.

### Seahorse assays

STAT1-knockdown iBMDM cells ectopically expressing STAT1α wild-type or ADPRylation site mutants were treated with Dox for 24 h to induce protein expression and seeded for Seahorse assays in Seahorse XFp cell Culture Miniplates (Agilent Technologies). Once adherent, the cells were treated with IFNγ for 16 h. The cell numbers in each well were quantified using the Celigo Imaging Cytometer-5 channel. Mitochondrial respiration was assessed by measuring the oxygen consumption rate (OCR) of the cells using the Seahorse XFp Cell Mito Stress Test kit (Agilent Technologies, 103010-100). The glycolytic rates of the cells were assessed using the Seahorse XF Glycolytic Rate Assay kit (Agilent Technologies, 103346-100). The Seahorse assays were performed according to manufacturer’s instructions and the measurements were taken using a Seahorse XFp Analyzer and analyzed using the Wave Desktop Software (Version 2.6).

### Reverse transcription-quantitative PCR (RT-qPCR)

cDNA pools were prepared from iBMDMs treated with IFNγ and veliparib using the RNeasy kit (Qiagen), followed by reverse transcription using MMLV reverse transcriptase (Promega, M150B) with oligo(dT) primers (Sigma-Aldrich). The cDNA was treated with 3 units of RNase H (Ambion) for 30 min at 37 °C and then analyzed by qPCR using the primer sets listed (Supplemental Table 1) and a LightCycler 480 real-time PCR thermocycler (Roche) for 45 cycles.

### RNA sequencing (RNA-seq)

RNA-seq libraries were prepared, sequenced, and analyzed as follows.

#### RNA isolation

Two replicates for the each of the different sets of BMDM or iBMDM cells were seeded at ~7.5 × 10^5^ cells per well in 6-well plates and treated as described above. The cells were collected and total RNA was isolated using the RNeasy kit (Qiagen) according to the manufacturer’s instructions.

#### RNA-seq library preparation

The RNA obtained above was used to generate strand-specific RNA-seq libraries using previously defined protocols^69^. Briefly, the total RNA was enriched for polyA+ RNA using Dynabeads Oligo(dT)25 (Invitrogen, 61002). The polyA+ RNA was then fragmented for 6 min at 94 °C and reverse transcribed using SuperScript III Reverse Transcriptase (Invitrogen, 18080093). Strand-specificity was ensured by using dUTP during the reverse transcription reaction. The cDNA generated was end-repaired and a single “A”-base overhang, was added using the Klenow fragment of E. coli DNA polymerase. The A-modified cDNA was ligated to Illumina sequencing adaptors. The ligated cDNA was size-selected using AMPure XP Beads (Agencourt, A50850). The DNA fragments were then UDG-digested, amplified using Illumina TruSeq P5 and P7 PCR primers and purified using agarose gel electrophoresis followed by gel extraction using the QIAquick Gel Extraction Kit (Qiagen, 28704). The RNA-seq libraries were subjected to QC analyses (i.e., number of PCR cycles required to amplify each library, the final library yield, and the size distribution of the final library DNA fragments) and sequenced using an Illumina HiSeq 2500 and NextSeq 500.

### Analysis of transcriptome data

#### Initial analysis of RNA-seq data

The raw data were subjected to QC analyses using the FastQC tool (Andrews et al., 2015). The reads were then mapped to the mouse genome (mm10) using the spliced reader aligner TopHat version.2.0.13 (Kim et al., 2013). Uniquely mappable reads were converted into bigWig files using BEDTools (version 2.17.0)^70^ for visualization in the Integrative Genomics Viewer (version 2.9.4)^71^. Transcriptome assembly was performed using cufflinks v.2.2.1^72^ with default parameters. The transcripts were merged into distinct, non-overlapping sets using cuffmerge, followed by cuffdiff to call the differentially regulated transcripts^72^. The significantly (q < 0.001) regulated genes were determined by comparing the experimental samples to corresponding untreated control samples to determine the regulated gene sets. The differentially expressed genes identified from the analysis described above were used in a number of subsequent downstream analyses and the data were visualized using a variety of approaches.

#### Data visualization and statistics

Venn diagrams were generated using jvenn^73^ for the differentially expressed genes in the different conditions. Heat maps were generated using Java TreeView^74^ for genes whose expression was significantly altered in at least one experimental condition. Box plot representations were used to quantitatively assess the log_2_ fold changes for genes in the different experimental conditions compared to matched untreated controls. Box plots were generated using custom scripts in R. Wilcoxon rank sum tests were performed to determine the statistical significance of all comparisons. Line plots were generated using custom scripts in R to represent the trend of the log_2_ fold changes of genes in the different experimental conditions compared to matched untreated controls.

#### Gene ontology analysis

Gene ontology (GO) analyses were done using the DAVID (Database for Annotation, Visualization, and Integrated Discovery)6.8 tool^75^. DAVID returns clusters of related ontological terms that are ranked according to an enrichment score.

### Chromatin immunoprecipitation sequencing (ChIP-seq)

ChIP-seq libraries were prepared, sequenced, and analyzed as follows.

#### Growth of cells

iBMDM cells were cultured and treated as described above in 15 cm diameter plates. BMDM cells were seeded at a density of 10 × 10^6^ cells per IP (for STAT1 ChIP) or 7.5 × 10^6^ cells per IP (for H3K27ac ChIP) in 15 cm diameter plates, and were treated with IFNγ with or without PJ34 for 1 h.

#### ChIP for STAT1 and H3K27ac

ChIP was performed as described previously^10,76^ with slight modifications. Briefly, the cells were cross-linked with 1% formaldehyde in PBS for 10 min at 37 °C and quenched in 125 mM glycine in PBS for 5 min at 4 °C. Cross-linked cells were then collected by centrifugation and lysed in Farnham Lysis Buffer (5 mM PIPES pH 8.0, 85 mM KCl, 0.5% NP-40, 1 mM DTT, 1 mM sodium fluoride, 1 mM sodium orthovanadate, 10 mM sodium butyrate, and 1x complete protease inhibitor cocktail). A crude nuclear pellet was collected by centrifugation, resuspended in Sonication Buffer (50 mM Tris-HCl pH 7.9, 1% SDS, 10 mM EDTA, 1 mM DTT, 1 mM sodium fluoride, 1 mM sodium orthovanadate, 10 mM sodium butyrate, and 1x complete protease inhibitor cocktail), and sonicated to generate chromatin fragments of ~300 bp in length. The soluble chromatin was clarified by centrifugation, diluted 1:10 with ChIP Dilution Buffer (20 mM Tris-HCl pH 7.9, 0.5% Triton X-100, 2 mM EDTA, 150 mM NaCl, 1 mM DTT, 1 mM sodium fluoride, 1 mM sodium orthovanadate, 10 mM sodium butyrate, and 1x complete protease inhibitor cocktail) and pre-cleared with protein A Dynabeads (Thermo Fischer, 1002D).

The pre-cleared samples were used in immunoprecipitation reactions with antibodies against STAT1, H3K27ac, or rabbit IgG (as a control) with incubation overnight at 4 °C with gently mixing. The samples were washed with (1) Low Salt Wash Buffer (20 mM Tris-HCl pH 7.9, 2 mM EDTA, 125 mM NaCl, 0.05% SDS, 1% Triton X-100, 1 mM sodium orthovanadate, 10 mM sodium butyrate, and 1x complete protease inhibitor cocktail), (2) High Salt Wash Buffer (20 mM Tris-HCl pH 7.9, 2 mM EDTA, 500 mM NaCl, 0.05% SDS, 1% Triton X-100, 1 mM sodium orthovanadate, 10 mM sodium butyrate, and 1x complete protease inhibitor cocktail), (3) LiCl Wash Buffer (10 mM Tris-HCl pH 7.9, 1 mM EDTA, 250 mM LiCl, 1% NP-40, 1% sodium deoxycholate, 1 mM sodium orthovanadate, 10 mM sodium butyrate, and 1x complete protease inhibitor cocktail), and (4) 1x Tris-EDTA (TE). The immunoprecipitated genomic DNA was eluted in Elution Buffer (100 mM NaHCO_3_, 1% SDS), digested with proteinase K and RNase H to remove protein and RNA, respectively, decrosslinked, extracted with phenol:chloroform:isoamyl alcohol, and precipitated with isopropanol. The precipitated ChIPed DNA was collected by centrifugation, air dried, and dissolved in DEPC- treated, nuclease-free water.

#### Preparation of ChIP-seq libraries

ChIP-seq libraries were generated from two biological replicates for each condition. A total of 5 ng (For STAT1) or 10 ng (For H3K27ac) of ChIPed DNA, or equivalent amounts input DNA, were used to generate libraries for sequencing. ChIP-seq libraries were generated based on previous protocols^77^. Briefly, the DNA was end-repaired and a single “A”-base overhang, was added using the Klenow fragment of E. coli DNA polymerase. The A-modified DNA was ligated to Illumina sequencing adaptors. The ligated DNA fragments were amplified using Illumina TruSeq P5 and P7 PCR primers, size-selected using agarose gel electrophoresis and sequenced using Illumina HiSeq 2500.

### Analysis of ChIP-seq data

#### Initial analysis of ChIP-seq data

The raw reads were aligned to the mouse reference genome (mm10) using default parameters in Bowtie (ver. 1.0.0)^78^. The aligned reads were subsequently filtered for quality and uniquely mappable reads using Samtools (ver. 0.1.19)^79^ and Picard (ver. 1.127; http://broadinstitute.github.io/picard/). Library complexity was measured using BEDTools (version 2.17.0)^70^ and met the minimum ENCODE data quality standards^80^. Relaxed peaks were called using MACS (ver. 2.1.0)^81^ and a default *p*-value = 1 × 10^-2^ for each replicate and input condition as a control. Final peaks for each condition were determined based on called peaks that overlapped in both replicates and were used for subsequent analysis.

#### Peak annotation and clustering

The peaks that were ‘gained’, ‘maintained’, and ‘depleted’ in response to the experimental conditions were identified as described below^82^. The reads under the peaks for each treatment were calculated for the treated sample (T2) and untreated control (T1). Rc was calculated using the following formula**:** Rc = log(T1/T2). Larger Rc values indicate binding enrichment upon co-treatment, while smaller Rc values indicate binding depletion. Median absolute deviation (MAD) was calculated for the Rc values and used as a cutoff to define ‘gained’, ‘depleted,’ and ‘maintained’ peaks.

#### Data visualization and statistics

To express the ChIP-seq peak data as Venn diagrams, we determined the overlap of peaks between conditions using the mergePeaks function in the HOMER software suite (version 4.9)^83^. Venn Diagrams were generated using jvenn^73^ for the overlapping peaks. To express the ChIP-seq peak data as heatmaps, we calculated the read densities 5 kb surrounding (±2.5 kb) the ‘gained’, ‘maintained’, and ‘depleted’ peaks using HOMER software^83^. The data were visualized as heatmaps using Java TreeView^74^. We used metagene representations to illustrate the distribution of reads near the STAT1α binding sites. The metagene analyses was performed using Deeptools 2.0^84^. The plots represent a smoothed average of read density weighted by expression over the set of STAT1α binding sites included in the analysis. Separate metagene representations were generated for the ‘gained’, ‘maintained’, and ‘depleted’ STAT1α binding sites upon IFNγ treatment with or without PJ34. Box plots were generated for quantitatively assessing the read distribution in a fixed window around each binding site under various conditions. The read distribution surrounding the peak center was calculated and plotted using the box plot function in R. The reads were normalized in the similar fashion as they were in the metagene analysis. Wilcoxon rank sum tests were performed to determine the statistical significance of all comparisons. Browser tracks were generated using bigWig files that represented fold change in signal for each condition relative to its input. Browser tracks were visualized using visualization in the Integrative Genomics Viewer^71^.

#### Nearest neighboring gene analyses

The nearest neighbor gene for each identified peak was determined using GREAT (version 3.0.0)^85^ within a specified distance from the peak summit. The expression of these genes was determined using the RPKM values obtained from the RNA-seq, using custom R scripts.

#### Motif analyses

De novo motif analyses were performed on a 200 bp region surrounding the peak summit (±100 bp) using the command-line version of MEME (version 5.3.3). The following parameters were used for motif prediction: (1) zero or one occurrence per sequence (-mod zoops); (2) number of motifs (-nmotifs 12); (3) minimum, maximum width of the motif (-minw 8, -maxw 15); and (4) search for motif in given strand and reverse complement strand (-revcomp). The predicted motifs from MEME were matched to known motifs using TOMTOM (version 5.3.3)^86^.

### Reporting summary

Further information on research design is available in the Nature Research Reporting Summary linked to this article.

## Supplementary information

Supplementary Information

Description of Additional Supplementary Files

Supplementary Data 1

Reporting Summary

## Data Availability

The RNA-seq and ChIP-seq datasets generated for this study can be accessed from the NCBI’s Gene Expression Omnibus (GEO) repository (www.ncbi.nlm.nih.gov/geo/) using accession number GSE147960. The mass spectrometry data sets generated for this study are provided with the manuscript (Supplemental Data 1). The authors declare that all other data supporting the findings of this study are available within the paper. Source data are provided with this paper. All data is available from the authors upon reasonable request. Source data are provided with this paper.

## Source data

Source Data

## Code availability

All custom scripts and codes used for sequencing data analysis will be made available upon request.
