# Supplementary material for: Nuclear ADP-ribosylation drives IFNγ-dependent STAT1α enhancer formation in macrophages
Source: Nat Commun. 2021 Jun 24;12:3931. doi: 10.1038/s41467-021-24225-2 (PMC8225886; doi:10.1038/s41467-021-24225-2)

Supplementary Information

"Nuclear ADP-Ribosylation Drives IFNγ-dependent STAT1α Enhancer Formation In Macrophages"

Gupte *et al.* (2021)

This document contains the following supplementary information:

Page

- **Supplementary Table 1.** List of Primers used 2
- **Supplementary Figure 1.** PARP-1 Regulates IFNγ-driven gene expression in macrophages. 4
- **Supplementary Figure 2.** PARP inhibition alters the IFNγ-dependent STAT1α cistrome 5
- **Supplementary Figure 3.** Loss of PARP-1 leads to attenuation of STAT1α phosphorylation at S727. 6
- **Supplementary Figure 4.** Inhibition of ADP-ribosylation by PJ34 leads to a loss of STAT1α phosphorylation at S727. 8
- **Supplementary Figure 5.** Identification of sites of ADP-ribosylation on STAT1α by using mass spectrometry. 10
- **Supplementary Figure 6.** Ectopic expression of STAT1α Wt and mutants in iBMDMs 11
- **Supplementary Figure 7.** IFNγ-dependent transcription in iBMDMs is regulated by ADP-ribosylation of STAT1α 12
- **Supplementary Figure 8.** Site-specific ADPRylation of STAT1α on its DBD and TA domain us required for pro-inflammatory responses in macrophages 14
- **Supplementary Figure 9.** Loss of site-specific ADP-ribosylation of the STAT1α DBD results in enhanced binding to DNA. 15
- **Supplementary Figure 10.** Loss of site-specific ADP-ribosylation of the STAT1α TA domain attenuates IFNγ-stimulated phosphorylation at S727 17

Supplementary Table 1. List of Primers Used

Primers used for cloning the Flag-tagged STAT1α cDNA	
Human STAT1α Forward	5’-ATGTCTCAGTGGTACGAACTTCAGCAGC-3’
Human STAT1α Reverse	5’- CTATACTGTGTTCATCATACTGTCGAA-3’
Mouse STAT1α Forward	5’ATGTCACAGTGGTTCGAGCTTCA-3’
Mouse STAT1α Reverse	5’-TTATACTGTGCTCATCATACTGTCAAATTCGGG-3’

Primers used for mutagenizing the STAT1α cDNA	
Human STAT1α DBD Mutant (E393/4Q) Forward	5’- GACTGCCATTGGTGGACTGCTGCATGTTCATCACTTTTGT-3’
Human STAT1α DBD Mutant (E393/4Q) Reverse	5’- ACAAAAGTGATGAACATGCAGCAGTCCACCAATGGCAGTC-3’
Human STAT1α TA Mutant (D721N) Forward	5’- GGAGCAGGTTGTTTGTGGTCTGAAGTCTAGAAGGG-3’
Human STAT1α TA Mutant (D721N) Reverse	5’- CCCTTCTAGACTTCAGACCACAAACAACCTGCTCC-3’
Mouse STAT1α DBD Mutant E393/4Q Forward	5’- CTGCCAGACTTCCGTTGGTGGACTGCTGCATGTTCATCACTTTTGTGTGC-3’
Mouse STAT1α DBD Mutant E393/4Q Reverse	5’-GCACACAAAAGTGATGAACATGCAGCAGTCCACCAACGGAAGTCTGGCAG-3’
Mouse STAT1α TA Mutant (D721N) Forward	5’- GAAGCAGGTTGTTTGTGGTCTGAAGTCTAGAAGGG-3’
Mouse STAT1α TA Mutant (D721N) Reverse	5’- CCCTTCTAGACTTCAGACCACAAACAACCTGCTTC-3’

qPCR Primers	
ccl7 Forward	5’- CCCATCAGAAGTGGGTCGAG-3’
ccl7 Reverse	5’- TGCTTCTTGGCTCCTAGGTTG-3’
cc12 Forward	5’- GCTGACCCCAAGGAGAAGTG-3’
ccl12 Reverse	5’- TGGGGAACTTCAGGGGGAAA-3’

[See Supplementary Figure 1 on the next page]

Supplementary Figure 1. PARP-1 Regulates IFNγ-driven gene expression in macrophages.
(a), (b) Expression of genes upregulated upon IFNγ treatment ± PJ34 in BMDMs. (a) Box plots showing the fold change in expression. Boxes represent 25th – 75th percentile (line at median) with whiskers at 1.5*IQR. Boxes marked with different letters are significantly different from each other (Wilcoxon Signed-Rank test; $p < 2.2 \times 10^{-16}$). Box plots represent 845 genes. (b) RNA-seq browser tracks showing the expression of *Crebzf* in the corresponding treatment conditions.
(c), (d) PARP-1 catalytic activity is required for the modulation of IFNγ-dependent gene expression in iBMDMs. Wild-type (Wt) and *Parp1* knockdown (KD) iBMDMs were treated with IFNγ ± PJ34 as indicated. Box plots showing the fold change in expression. Boxes represent 25th – 75th percentile (line at median) with whiskers at 1.5*IQR. Boxes marked with different letters are significantly different from each other (Wilcoxon Signed-Rank test; $p < 2.2 \times 10^{-16}$). Box plots represent 781 genes (c); 936 genes (2 h) and 665 genes (4 h) in (d).
(e) Experiments similar to those shown in (c) and (d). Bar graphs showing the relative mRNA levels upon treatment IFNγ treatment ± Veliparib (Vel.). The p-values were determined using one-way ANOVA followed by Fisher's LSD test (n = 3; * = 0.0138 for *Ccl12*; * = 0.0116 for *Ccl7*).

Supplementary Figure 1

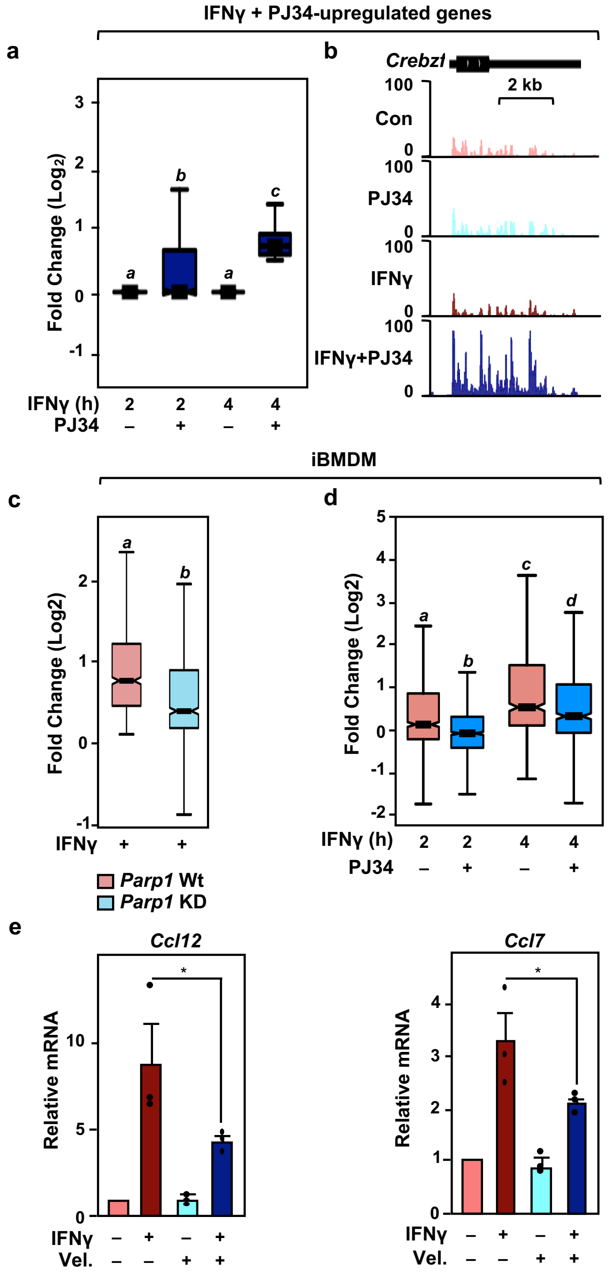

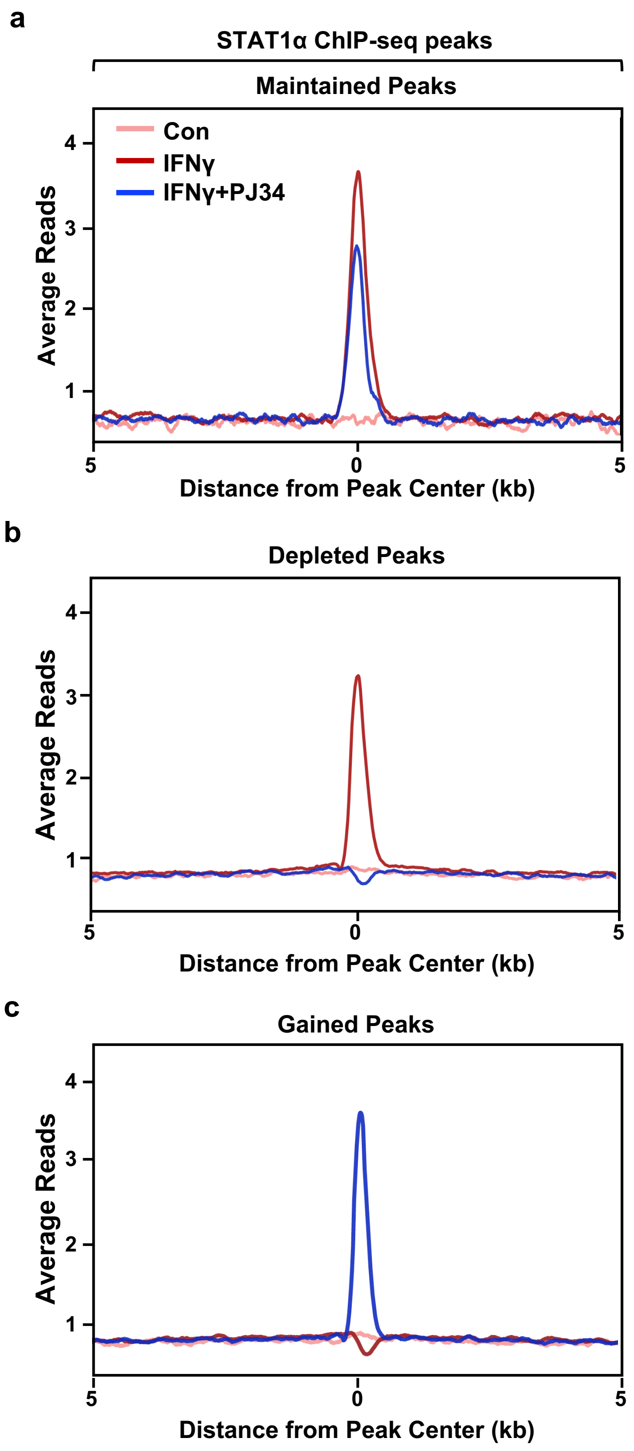

Supplementary Figure 2. PARP inhibition alters the IFNγ-dependent STAT1α cistrome. Metaplots showing the read density within a 10 kb window surrounding the peak center for 'maintained' (a), 'depleted' (b) and 'gained' (c) STAT1α peaks. ChIP-seq was performed on BMDMs as described in Figure 2.

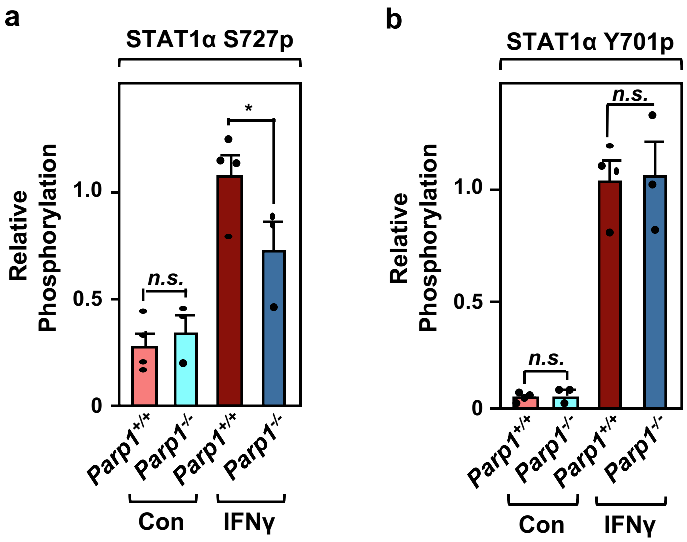

Supplementary Figure 3. Loss of PARP-1 leads to attenuation of STAT1α phosphorylation at S727.
(a), **(b)** Quantification of immunoblots (n = 3 or 4 as indicated) for STAT1α S727p (a) and STAT1α Y701p (b) relative to total STAT1α levels. β-Tubulin loading was used as a normalization control. The p-values were determined using two-way ANOVA followed by Tukey's multiple comparison tests (n.s., not significant at $p < 0.05$; * = 0.0204). Error bars represent mean ± SEM.

[See Supplementary Figure 4 on the next page]

Supplementary Figure 4. Inhibition of ADP-ribosylation by PJ34 leads to a loss of STAT1α phosphorylation at S727.

(a) Inhibition of PARP-1 catalytic activity does not affect IFNγ-induced STAT1α nuclear translocation. Immunofluorescent staining for STAT1α was performed in BMDM treated with IFNγ (1 hour) ± PJ34. The nuclei were visualized with DAPI staining. Scale bar: 10μm.

(b) Violin plots showing the quantification of immunofluorescent staining data from (A). The p-values were determined using one-way ANOVA followed by Tukey's multiple comparison tests (n.s., not significant at $p = 0.9791$).

(c), (d) Immunoblots for STAT1α S727p in iBMDMs (c) and THP1 cells (d). Bottom panels show quantification of the immunoblots for 3 independent experiments. (c) The p-values were determined using paired, two-tailed Student's t-test (* = 0.0403; n.s. > 0.05). (d) The p-values were determined using two-way ANOVA followed by Tukey's multiple comparison tests (* = 0.0130; n.s. > 0.05). Error bars represent mean ± SEM. Uncropped immunoblots are provided as a Source Data file.

(e) Immunoblots for STAT1α S727p in iBMDMs treated with IFNγ ± Veliparib (Vel.) as indicated. Right panel shows quantification of the immunoblots for 3 independent experiments. The p-values were determined using two-way ANOVA followed by Fisher's LSD tests (* = 0.0127; n.s. > 0.05). Error bars represent mean ± SEM. Uncropped immunoblots are provided as a Source Data file.

(f) Immunoblots for STAT1α S727p in iBMDMs treated with IFNγ or LPS as indicated. The immunoblots are representative of 2 independent experiments. Uncropped immunoblots are provided as a Source Data file.

Supplementary Figure 4

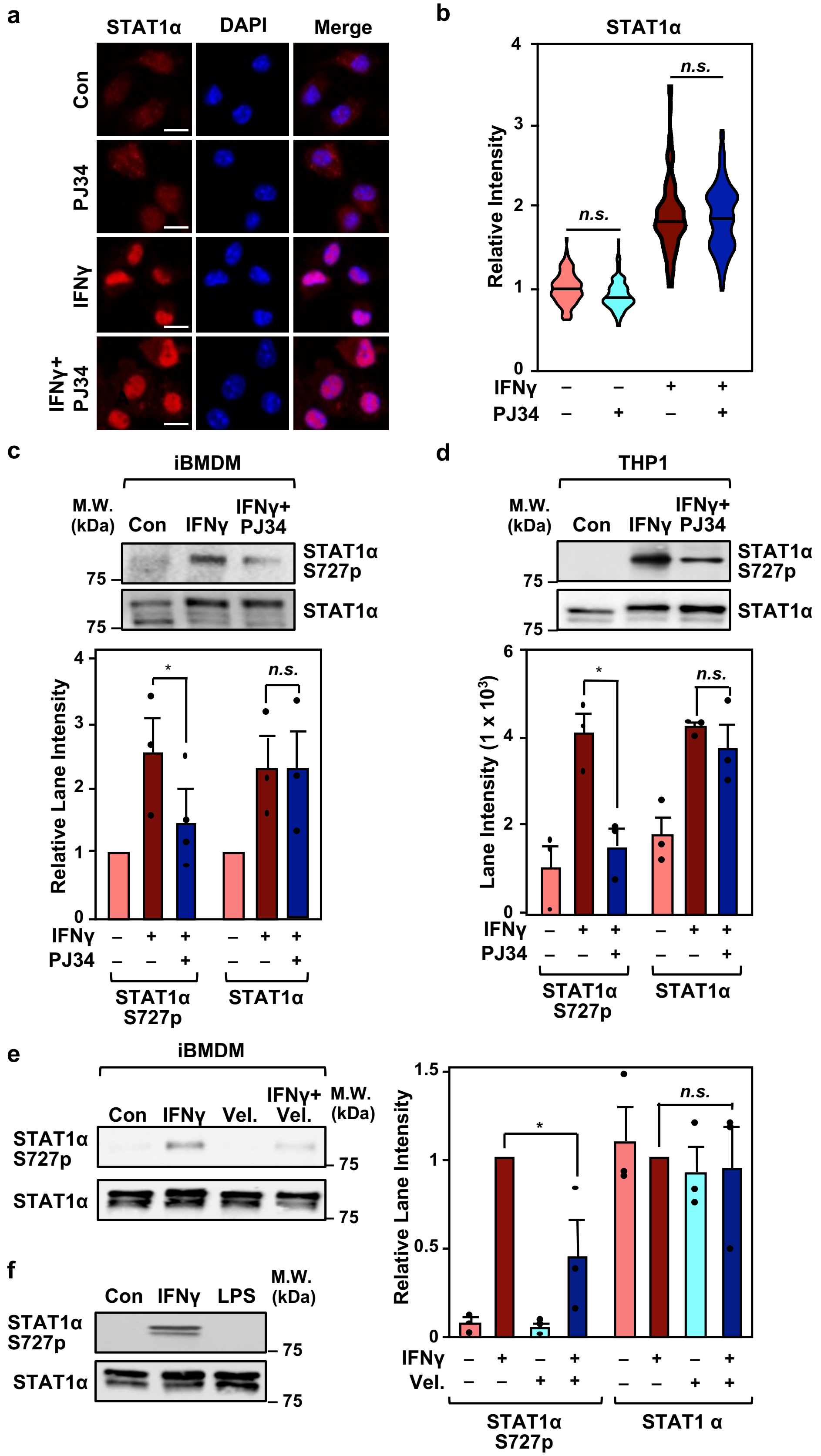

[See Supplementary Figure 5 on the next page]

Supplementary Figure 5. Identification of sites of ADP-ribosylation on STAT1α by using mass spectrometry.

(a) STAT1α is ADPRylated in IFNγ-treated iBMDMs. Immunoblots showing ADPRylation in iBMDMs expressing Flag-tagged STAT1α and stimulated with IFNγ ± PJ34. The ectopically expressed STAT1α was immunoprecipitated from nuclear extracts using a Flag antibody. PAR levels were detected using an ADP-ribose detection reagent (AF1521-Fc reagent). The immunoblots are representative of 3 independent experiments. Uncropped immunoblots are provided as a Source Data file.

(b) Immunoblots showing ADPRylation of STAT1α by PARP-1 in vitro from Fig. 4b. Molecular weight (M.W.) markers are used to show corresponding protein sizes in the top panel (PAR). Arrow indicates the band for ADPRylated STAT1α. The immunoblots are representative of 3 independent experiments. Uncropped immunoblots are provided as a Source Data file.

(c) *(Top)* Schematic diagram showing the protein coverage obtained from mass spectrometry assays of endogenous STAT1α (iBMDMs) and ectopically expressed STAT1α (293T cells). *(Bottom)* Schematic diagram of STAT1α showing its functional domains, including the N-terminal (N), coiled-coil (CC), DNA binding (DBD), Src Homology 2 (SH2), and transactivation (TA) domains.

(d) The sites of ADP-ribosylation on STAT1α on selected peptides determined by mass spectrometry.

(e) Quantification of immunoblots from Fig. 4e for 3 independent experiments (two-way ANOVA followed by Dunnett's multiple comparisons test; * = 0.022 , ** = 0.0006). Error bars represent mean ± SEM.

(f) Immunoblots showing total ADPRylation in iBMDMs ectopically STAT1α wildtype (Wt) or ADPRylation-defective mutants (DBDmut and TAmut). iBMDMs were stimulated with IFNγ as indicated. The immunoblots are representative of 3 independent experiments. Uncropped immunoblots are provided as a Source Data file.

Supplementary Figure 5

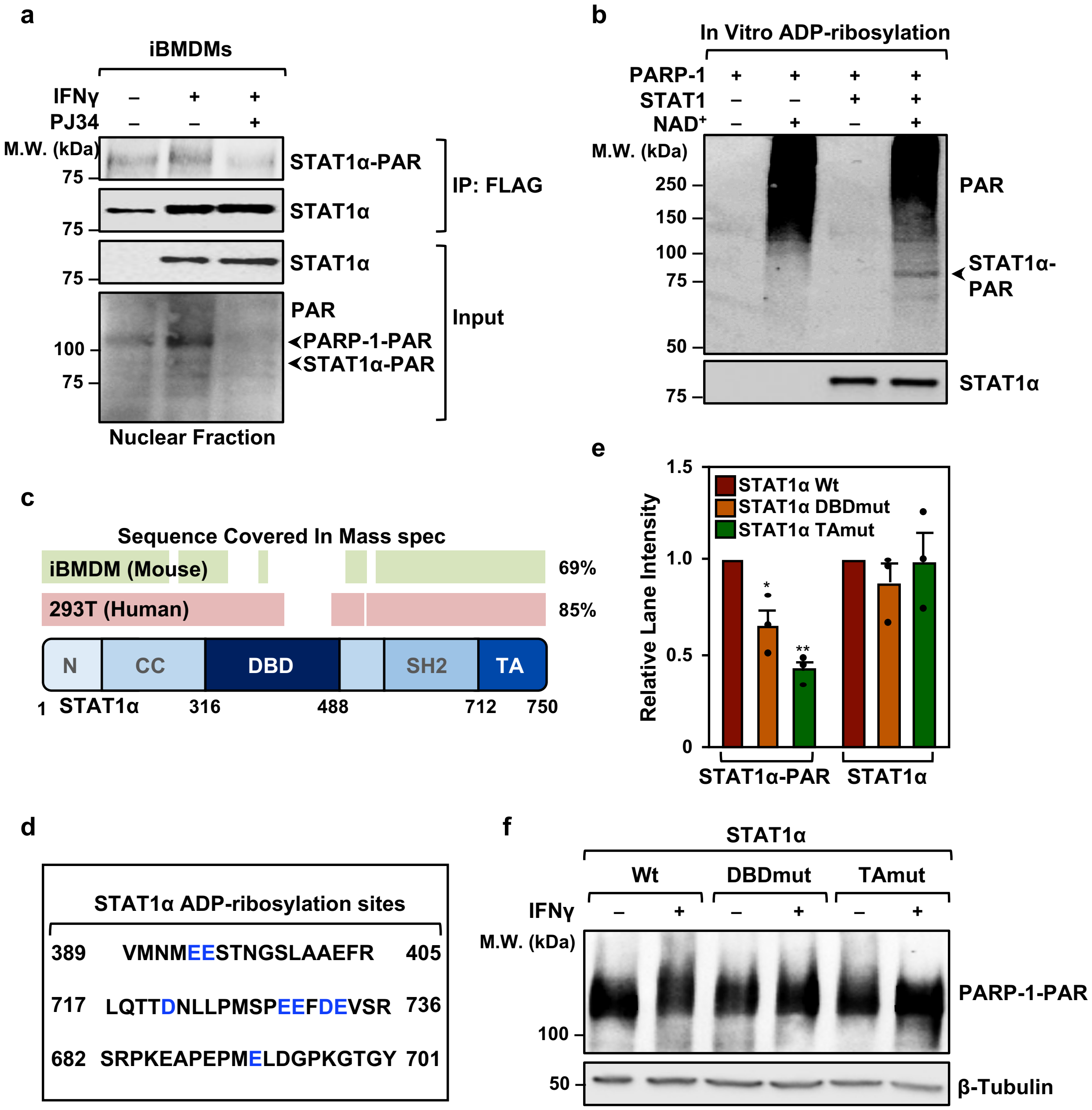

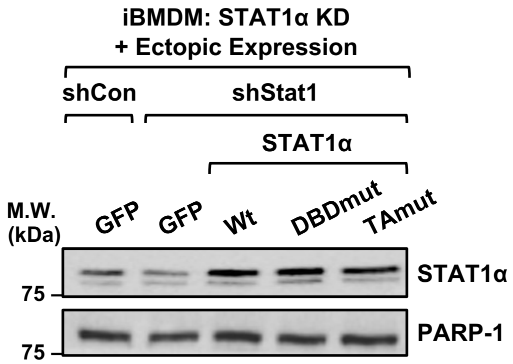

Supplementary Figure 6. Ectopic expression of STAT1α Wt and mutants in iBMDMs. Immunoblots showing inducible ectopic expression of STAT1α wild-type (Wt) and ADP-ribosylation site mutants (DBDmut and TAmut) in iBMDMs subjected to concurrent shRNA-mediated knockdown of endogenous STAT1α (shStat1) or a control knockdown (shCon). The immunoblots are representative of 3 independent experiments. Uncropped immunoblots are provided as a Source Data file.

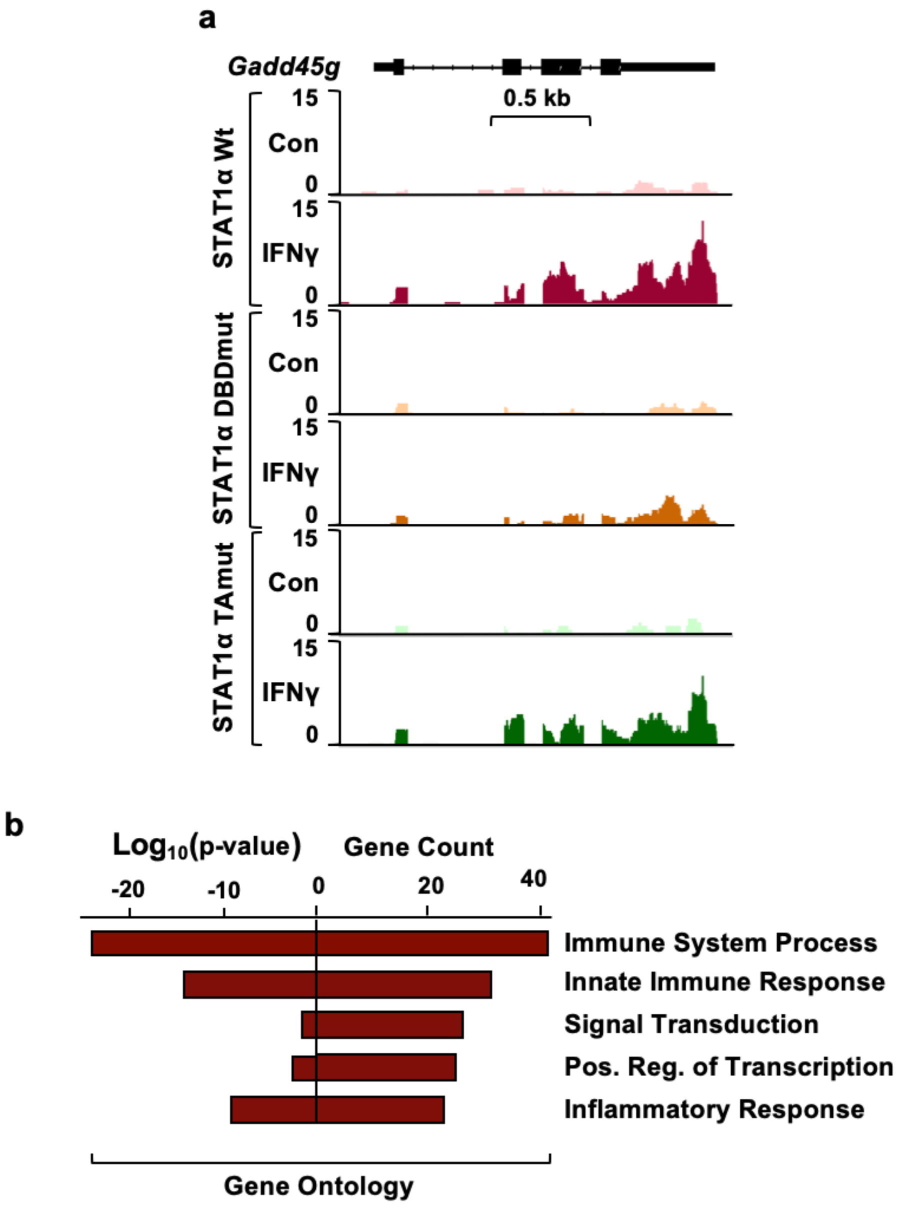

Supplementary Figure 7. IFNγ-dependent transcription in iBMDMs is regulated by ADP-ribosylation of STAT1α.

(a) RNA-seq browser tracks showing IFNγ-induced gene expression at the indicated locus (*Gadd45g*). RNA-seq was performed in iBMDMs ectopically expressing Wt or ADP-ribosylation site mutants (DBDmut and TAmut) with concurrent shRNA-mediated knockdown of endogenous STAT1α).

(b) Gene ontology analysis for IFNγ-stimulated genes that are dependent on STAT1α ADP-ribosylation for proper expression. The gene counts in each term and the $\log_{10}$(p-value) for each term are shown.

[See Supplementary Figure 8 on the next page]

Supplementary Figure 8. Site-specific ADPRylation of STAT1α on its DBD and TA domain us required for pro-inflammatory responses in macrophages.

(a) Schematic diagram showing the role of site-specific ADPRylation of STAT1α in downstream pro-inflammatory responses in macrophages.

(b) Quantification of phagocytosis showing the number of $pHrodo^{+}$ cells relative to the total number of cells per field for 3 independent experiments (unpaired, two-tailed Student's t-test; ** = 0.0019, *** = 0.0007). Error bars represent mean ± SEM.

(c), **(d)** PARP catalytic inhibition results in reduced phagocytotic capacity in macrophages. iBMDMs were treated with PJ34 as indicated. Scale bar: 44μm. (d) Quantification of phagocytosis showing the number of $pHrodo^{+}$ cells relative to the total number of cells per field for 3 independent experiments (unpaired, two-tailed Student's t-test; ** = 0.0049). Error bars represent mean ± SEM.

(e) PJ34 treatment does not significantly alter the relative amount of phagocytosis in iBMDMs expressing Wt or ADP-ribosylation site mutants. The p-values were determined using two-way ANOVA followed by Tukey's multiple comparison tests for 3 independent experiments (n.s., not significant at 0.05). Error bars represent mean ± SEM.

(f) PARP catalytic inhibition results in attenuation of NOS activity in iBMDMs. The cells were treated with IFNγ and veliparib for 24 hours as indicated (n = 3; unpaired, two-tailed Student's t-test; * = 0.0132). Error bars represent mean ± SEM.

(g), (h) ADP-ribosylation of STAT1α does not affect mitochondrial respiration. (g) Mitochondrial respiration profile of iBMDMs expressing Wt or ADP-ribosylation site mutant (DBDmut and TAmut) STAT1α using Seahorse assays. (h) Fold change in the level of basal mitochondrial respiration observed upon IFNγ treatment. In both panels, the assays were performed in three independent biological replicates. The significance determined using unpaired, two-tailed Student's t-test for 3 independent experiments (the results with the ADP-ribosylation site mutants of STAT1α were not significantly different from the wild-type at $p < 0.05$). Error bars represent mean ± SEM.

Supplementary Figure 8

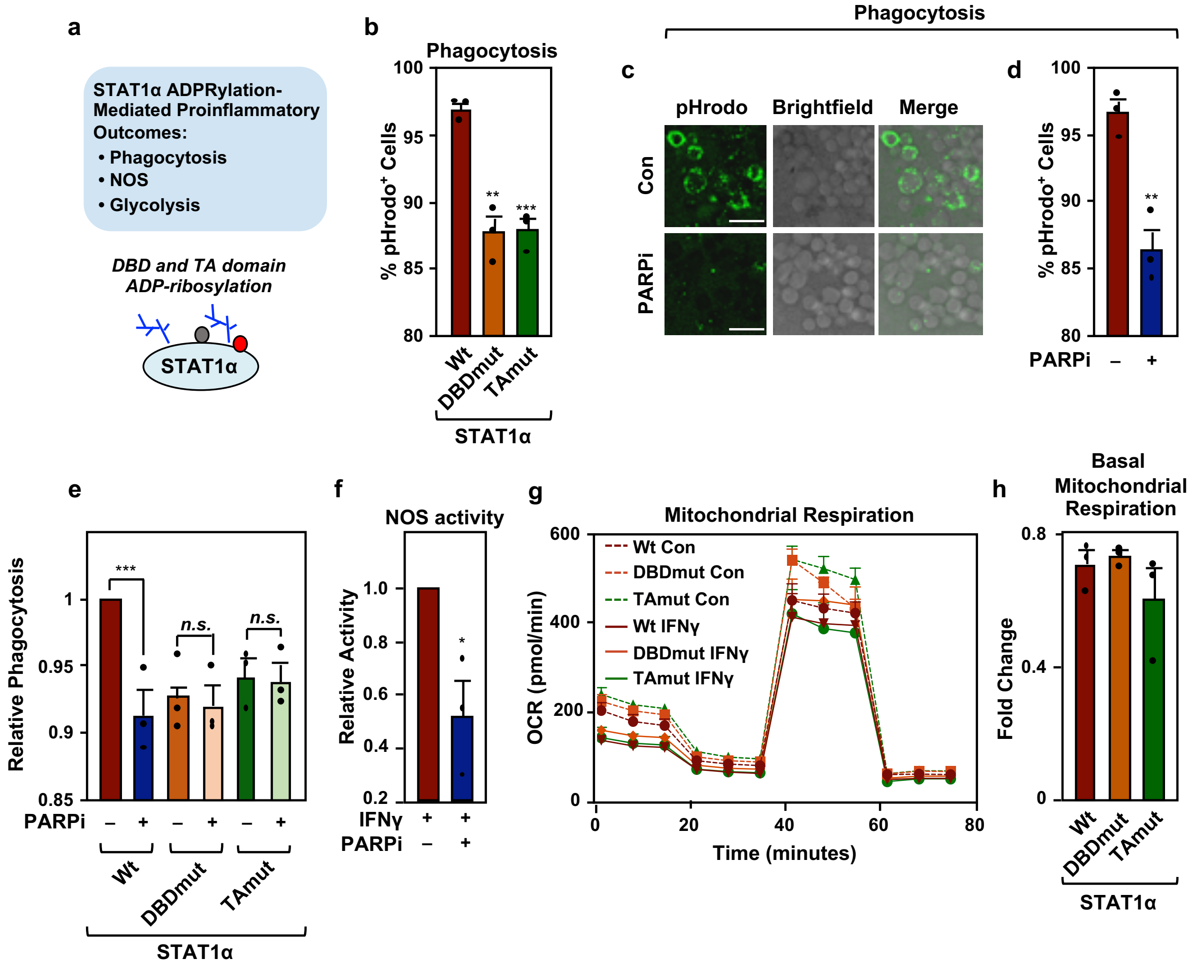

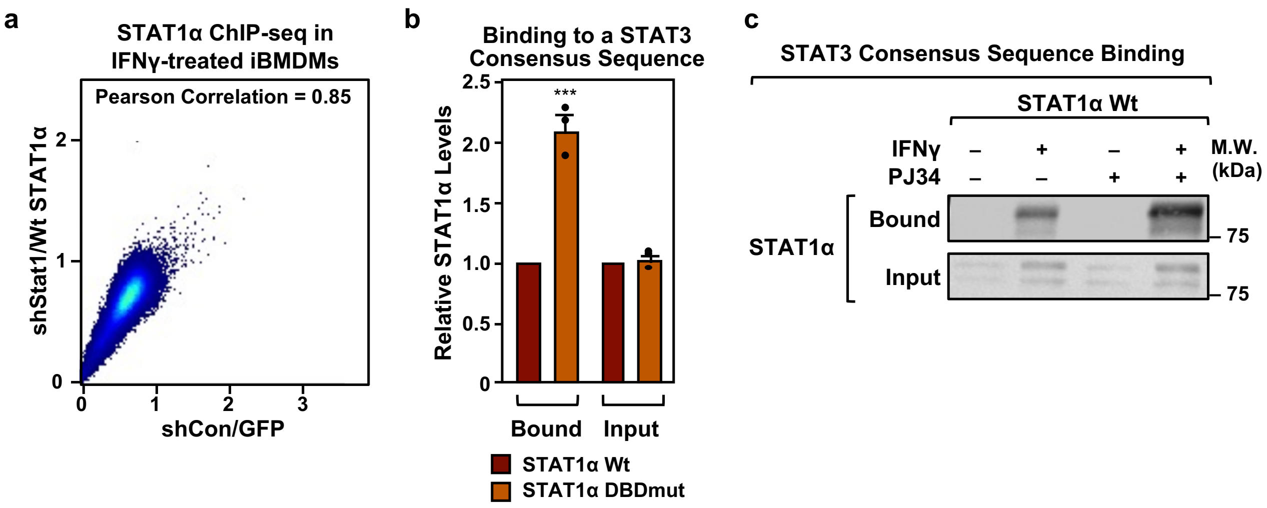

Supplementary Figure 9. Loss of site-specific ADP-ribosylation of the STAT1α DBD results in enhanced binding of STAT1α to DNA.

(a) Ectopically expressed STAT1α localizes to similar genomic loci as endogenous STAT1α. Correlation plots comparing ChIP-seq read accumulations between iBMDMs subjected to non-specific knockdown and ectopic expression of GFP (shCon/GFP), and knockdown of endogenous STAT1α and ectopic expression of Wt STAT1α (shStat1/STAT1α Wt).

(b) STAT1α DBDmut exhibits increased binding to a STAT3 consensus sequence. Quantification of immunoblots from Fig. 5f showing the levels of STAT1α binding under the conditions indicated (i.e., with ectopic expression of Wt or DBDmut STAT1α and concurrent shRNA-mediated knockdown of endogenous STAT1α). The assays were performed in three independent biological replicates. The p-values were determined using unpaired Student's t-test followed by Holm-Sidak's multiple comparison test (*** = 0.000881). Error bars represent mean ± SEM.

(c) Inhibition of PARP-mediated ADPRylation of STAT1α results in increased binding to a STAT3 consensus sequence. iBMDMs were treated with IFNγ ± PJ34 as indicated. The immunoblots are representative of 3 independent experiments. Uncropped immunoblots are provided as a Source Data file.

[See Supplementary Figure 10 on the next page]

Supplementary Figure 10. Loss of site-specific ADP-ribosylation of the STAT1α TA domain can attenuate IFNγ-stimulated phosphorylation at S727.

(a) Quantification of immunoblots from Fig. 6B showing the levels of STAT1α S727p in iBMDMs under the conditions indicated (i.e., with ectopic expression of Wt, DBDmut, or TAmut STAT1α and concurrent shRNA-mediated knockdown of endogenous STAT1α). The assays were performed in three independent biological replicates. The p-values were determined using two-way ANOVA followed by Sidak's multiple comparison test (** = 0.004, n.s., not significant). Error bars represent mean ± SEM.

(b) Immunoblots showing STAT1α S727p in 293T transiently expressing STAT1α Wt and ADP-ribosylation site mutants (DBDmut and TAmut). The immunoblots are representative of 3 independent experiments. Uncropped immunoblots are provided as a Source Data file.

(c) SDS-PAGE with Coomassie Blue staining showing the p300 purified from SF9 insect cells. The band corresponding to purified p300 protein is marked with a red arrow. The image is representative for one round of purification. Uncropped gel images are provided as a Source Data file.

(d) PARP-1 autoactivation attenuates its acetylation by p300. Immunoblots showing the acetylation of PARP-1 from in vitro reactions performed in the presence or absence of NAD^+. The immunoblots are representative of 2 independent experiments. Uncropped immunoblots are provided as a Source Data file.

(e) Phosphorylation of STAT1α on S727 is required for p300 autoacetylation. Immunoblots show autoacetylation of p300 in the presence of STAT1α Wt or S727A mutant as indicated. The immunoblots are representative of 3 independent experiments. Uncropped immunoblots are provided as a Source Data file.

(f) Browser tracks of ChIP-seq data showing STAT1α and H3K27ac enrichment in Wt- or TAmut-expressing iBMDMs.

(g) Heatmaps of ChIP-seq data representing the top 50% of 'maintained' STAT1α peaks in TAmut-expressing iBMDMs compared to Wt STAT1α-expressing iBMDMs.

Supplementary Figure 10.

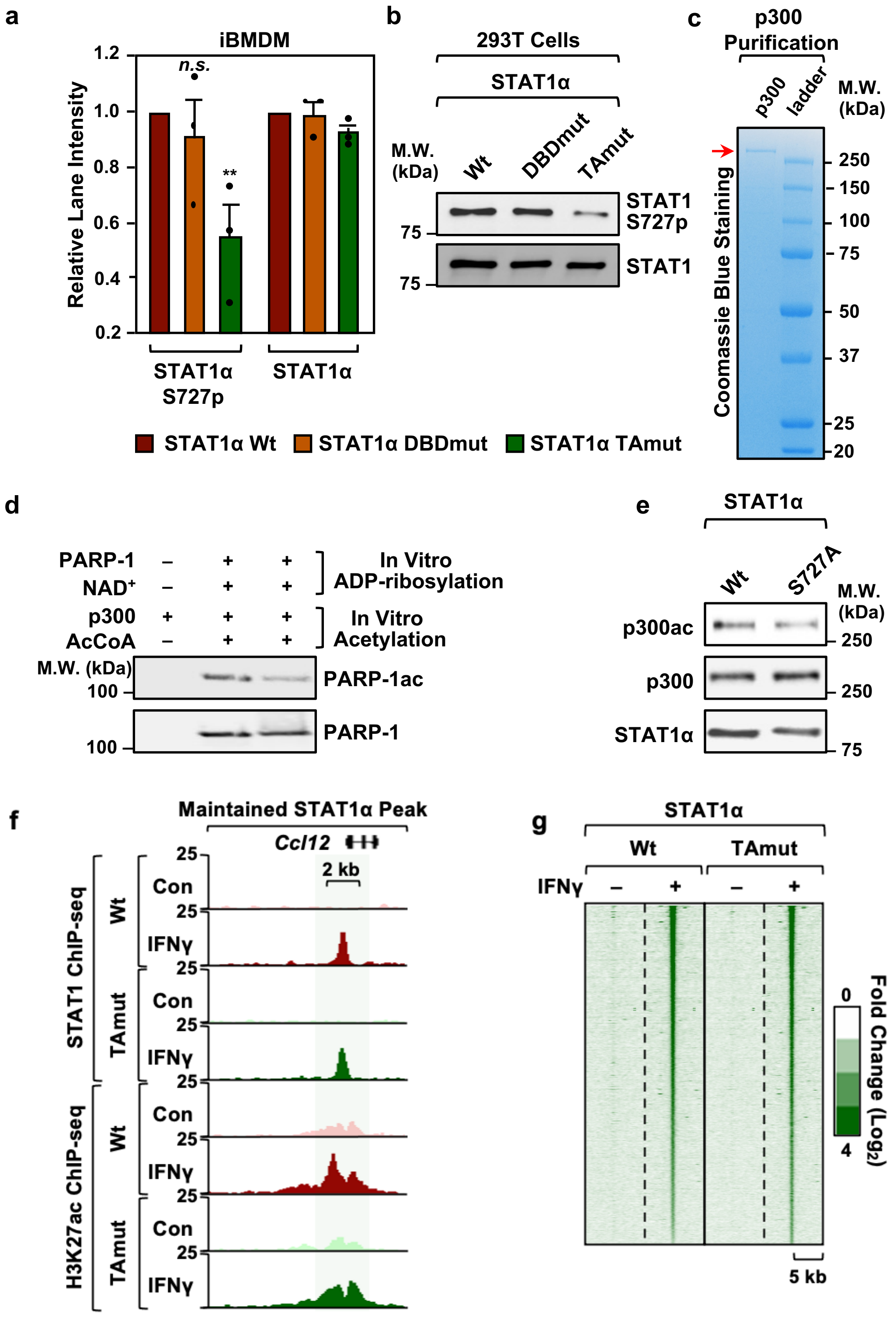

Supplement: Supplementary file 1 — Supplementary Information [file 41467_2021_24225_MOESM1_ESM.pdf]
