# Supplementary material for: Nuclear ADP-ribosylation drives IFNγ-dependent STAT1α enhancer formation in macrophages
Source: Nat Commun. 2021 Jun 24;12:3931. doi: 10.1038/s41467-021-24225-2 (PMC8225886; doi:10.1038/s41467-021-24225-2)
Supplement: Supplementary file 2 — Description of Additional Supplementary Files [file 41467_2021_24225_MOESM2_ESM.pdf]

Description of Additional Supplementary Files

File Name: Supplementary Data 1
Description: Mass spectrometry data for STAT1α ADPRylation site identification.
