# Supplementary material for: Nuclear ADP-ribosylation drives IFNγ-dependent STAT1α enhancer formation in macrophages
Source: Nat Commun. 2021 Jun 24;12:3931. doi: 10.1038/s41467-021-24225-2 (PMC8225886; doi:10.1038/s41467-021-24225-2)
Supplement: Supplementary file 4 — Reporting Summary [file 41467_2021_24225_MOESM4_ESM.pdf]

nature research

Corresponding author(s): W. Lee Kraus

Last updated by author(s): May 18, 2021

Reporting Summary

Nature Research wishes to improve the reproducibility of the work that we publish. This form provides structure for consistency and transparency in reporting. For further information on Nature Research policies, see Authors & Referees and the Editorial Policy Checklist.

Statistics

For all statistical analyses, confirm that the following items are present in the figure legend, table legend, main text, or Methods section.

n/a	Confirmed	
☐	☒	The exact sample size (n) for each experimental group/condition, given as a discrete number and unit of measurement
☐	☒	A statement on whether measurements were taken from distinct samples or whether the same sample was measured repeatedly
☐	☒	The statistical test(s) used AND whether they are one- or two-sided<br>*Only common tests should be described solely by name; describe more complex techniques in the Methods section.*
☒	☐	A description of all covariates tested
☒	☐	A description of any assumptions or corrections, such as tests of normality and adjustment for multiple comparisons
☐	☒	A full description of the statistical parameters including central tendency (e.g. means) or other basic estimates (e.g. regression coefficient) AND variation (e.g. standard deviation) or associated estimates of uncertainty (e.g. confidence intervals)
☐	☒	For null hypothesis testing, the test statistic (e.g. F, t, r) with confidence intervals, effect sizes, degrees of freedom and P value noted<br>*Give P values as exact values whenever suitable.*
☒	☐	For Bayesian analysis, information on the choice of priors and Markov chain Monte Carlo settings
☒	☐	For hierarchical and complex designs, identification of the appropriate level for tests and full reporting of outcomes
☐	☒	Estimates of effect sizes (e.g. Cohen's d, Pearson's r), indicating how they were calculated

Our web collection on statistics for biologists contains articles on many of the points above.

Software and code

Policy information about availability of computer code

Data collection	NextGen sequence data was generated using the Illumina platform with the HiSeq control software (version 2.2.68). Image data was collected using a Zeiss LSM 880 Airyscan confocal microscope with the Zeiss Zen Imaging Software (version 3.3). Mass spec data were collected using an Orbitrap Fusion Lumos mass spectrometer (Thermo Electron) with the Orbitrap Tribrid MS Series Instrument Control Software Version 3.4. Seahorse measurements were taken using a Seahorse XFp Analyzer and analyzed using the Wave Desktop Software (Version 2.6).
Data analysis	Mass spec data were analyzed using the central proteomics facilities pipeline (CPFP), version 2.0.3. Peptide identification was performed using the X!Tandem (2017.02.01) and open MS search algorithm (OMSSA) search engines against the human and mouse protein database from Uniprot.<br>RNA-seq were subjected to QC analyses using the FastQC tool (version 0.11.4). The reads were then mapped to mouse genome (mm10) using the spliced reader aligner TopHat version 2.0.13. Transcriptome assembly was performed using Cufflinks version 2.2.1 with default parameters. Uniquely mappable reads were converted into bigWig files using BEDTools (version 2.17.0) for visualization in the Integrative Genomics Viewer (version 2.9.4). Gene ontology (GO) analyses were done using the DAVID (Database for Annotation, Visualization, and Integrated Discovery)6.8 tool.<br>ChIP-seq reads were were aligned to the mouse reference genome (mm10) using default parameters in Bowtie version 1.0.0. The aligned reads were filtered for quality and uniquely mappable reads using Samtools version 0.1.19 and Picard (ver. 1.127). Library complexity was measured using BEDTools version 2.17.0. Relaxed peaks were called using MACS version 2.1.0. The overlap of peaks between conditions was determined using the mergePeaks function in the HOMER software suite (v. 4.9). The metagene analyses was performed using Deeptools 2.0. The nearest neighbor gene for each identified peak was determined using GREAT version 3.0.0. De novo motif analyses were performed using the command-line version of MEME Version 5.3.3. The predicted motifs from MEME were matched to known motifs using TOMTOM (version 5.3.3).<br>Images were analyzed using ImageJ software (Fiji).<br>Custom scripts and codes are available on request

For manuscripts utilizing custom algorithms or software that are central to the research but not yet described in published literature, software must be made available to editors/reviewers. We strongly encourage code deposition in a community repository (e.g. GitHub). See the Nature Research guidelines for submitting code & software for further information.

Data

Policy information about availability of data

All manuscripts must include a data availability statement. This statement should provide the following information, where applicable:

- Accession codes, unique identifiers, or web links for publicly available datasets
- A list of figures that have associated raw data
- A description of any restrictions on data availability

The RNA-seq and ChIP-seq data that support the findings in this study have been deposited in can be accessed in the NCBI's GEO database with the accession number GSE147960 . The mass spec data sets generated for this study are available as supplemental data provided with this manuscript (Supplemental Table 1). The authors declare that all other data supporting the findings of this study are available within the paper.

Field-specific reporting

Please select the one below that is the best fit for your research. If you are not sure, read the appropriate sections before making your selection.

☒ Life sciences ☐ Behavioural & social sciences ☐ Ecological, evolutionary & environmental sciences

For a reference copy of the document with all sections, see nature.com/documents/nr-reporting-summary-flat.pdf

Life sciences study design

All studies must disclose on these points even when the disclosure is negative.

Sample size	No prior sample-size calculation was performed. Sample sizes for cells lines and other biological materials were chosen based on prior experience of the "n" required to achieve significance given the experiential variation as determined in our previous work (Luo et al., 2017; Kim et al., 2019).
Data exclusions	No exclusions were made.
Replication	All the experiments were performed with a minimum of three independent collections and a significance of p-value <0.05 except RNA-seq and ChIP-seq, which were performed from two independent biological replicates.
Randomization	Plates of cultured cells were randomly distributed into groups used to transfect with constructs or infect with lentivirus for ectopic expression or knockdown. They were then distributed by transgene into the experimental groups. Bone marrow derived macrophages from different mice were pooled together before plating for experimental purposes.
Blinding	No blinded conditions used as blinding was not applicable to the study. We used a number of analysis softwares to calculate the results and included all the independent experimental replicates.

Reporting for specific materials, systems and methods

We require information from authors about some types of materials, experimental systems and methods used in many studies. Here, indicate whether each material, system or method listed is relevant to your study. If you are not sure if a list item applies to your research, read the appropriate section before selecting a response.

Materials & experimental systems

n/a	Involved in the study	
☐	☒	Antibodies
☐	☒	Eukaryotic cell lines
☒	☐	Palaeontology
☐	☒	Animals and other organisms
☒	☐	Human research participants
☒	☐	Clinical data

Methods

n/a	Involved in the study	
☐	☒	ChIP-seq
☒	☐	Flow cytometry
☒	☐	MRI-based neuroimaging

Antibodies

Antibodies used	The antibodies used for immunoblotting included STAT1 rabbit polyclonal antibody (Cell Signaling, 9172L); Phospho-STAT1 (Ser727) rabbit monoclonal antibody (Cell Signaling, 8826S); Phospho-STAT1 (Ser727) rabbit polyclonal antibody (Cell Signaling Technologies, 9177s); Phospho-STAT1 (Tyr701) rabbit polyclonal antibody (Cell Signaling Technologies, 9167); Flag mouse monoclonal antibody (Sigma-Aldrich, F3165); B-tubulin rabbit polyclonal antibody (Abcam, ab6046); p300 mouse monoclonal antibody (Active motif, 61401); Acetyl-CBP (Lys1535)/p300 (Lys1499) rabbit polyclonal antibody (Cell Signaling, 4771); rabbit IgG (ThermoFisher Scientific, 10500C); goat anti-rabbit HRP-conjugated IgG (Pierce, 31460); and goat anti-mouse HRP-conjugated IgG (Pierce, 31430). The custom rabbit polyclonal antiserum against PARP-1 was generated by using an antigen comprising the

	amino-terminal half of PARP-1 (now available from Active Motif; cat. no. 39559). The custom recombinant antibody-like anti-ADP-ribose binding reagents were generated and purified in-house (now available from EMD Millipore; cat. no. MABE1031, MABE1016).<br>STAT1 (Santa Cruz Biotech, sc-592) and Histone H3 (acetyl K27) (Abcam, ab4729) rabbit polyclonal antibodies were used for chromatin immunoprecipitation assays.<br>STAT1 rabbit polyclonal antibody (Cell Signaling, 9172L); Phospho-STAT1 (Ser727) rabbit polyclonal antibody (Cell Signaling Technologies, 9177s); Alexa Fluor 594 donkey anti-rabbit IgG (ThermoFisher, A-21207) were used for autofluorescence.
Validation	The custom recombinant antibody-like anti-ADP-ribose binding reagents were validated as described in Gibson et al., 2017.<br><br>The validation of the commercially available antibodies was performed by the companies as stated.<br>STAT1 rabbit polyclonal antibody (Cell Signaling, 9172L) https://www.cellsignal.com/products/primary-antibodies/stat1-antibody/9172<br>Phospho-STAT1 (Ser727) rabbit monoclonal antibody (Cell Signaling, 8826S) https://www.cellsignal.com/products/primary-antibodies/phospho-stat1-ser727-d3b7-rabbit-mab/8826<br>Phospho-STAT1 (Ser727) rabbit polyclonal antibody (Cell Signaling Technologies, 9177s) https://www.cellsignal.com/products/primary-antibodies/phospho-stat1-ser727-antibody/9177<br>Phospho-STAT1 (Tyr701) rabbit polyclonal antibody (Cell Signaling Technologies, 9167) https://www.cellsignal.com/products/primary-antibodies/phospho-stat1-tyr701-58d6-rabbit-mab/9167<br>Flag mouse monoclonal antibody (Sigma-Aldrich, F3165) https://www.sigmaaldrich.com/catalog/product/sigma/f3165?lang=en&region=US<br>B-tubulin rabbit polyclonal antibody (Abcam, ab6046) https://www.abcam.com/beta-tubulin-antibody-loading-control-ab6046.html<br>p300 mouse monoclonal antibody (Active motif, 61401) https://www.activemotif.com/catalog/details/61401<br>Acetyl-CBP (Lys1535)/p300 (Lys1499) rabbit polyclonal antibody (Cell Signaling, 4771) https://www.cellsignal.com/products/primary-antibodies/acetyl-cbp-lys1535-p300-lys1499-antibody/4771<br>rabbit IgG (ThermoFisher Scientific, 10500C) https://www.thermofisher.com/antibody/product/Rabbit-IgG-Isotype-Control/10500C<br>goat anti-rabbit HRP-conjugated IgG (Pierce, 31460) https://www.thermofisher.com/antibody/product/Goat-anti-Rabbit-IgG-H-L-Secondary-Antibody-Polyclonal/31460<br>goat anti-mouse HRP-conjugated IgG (Pierce, 31430) https://www.thermofisher.com/antibody/product/Goat-anti-Mouse-IgG-H-L-Secondary-Antibody-Polyclonal/31430<br>PARP-1 (Active Motif; cat. no. 39559) https://www.activemotif.com/catalog/details/39559/parp-1-n-terminal-antibody-pab<br>STAT1 (Santa Cruz Biotech, sc-592) https://www.scbt.com/p/stat1-p84-p91-antibody-m-22<br>Histone H3 (acetyl K27) (Abcam, ab4729) https://www.abcam.com/histone-h3-acetyl-k27-antibody-chip-grade-ab4729.html<br>Alexa Fluor 594 donkey anti-rabbit IgG (ThermoFisher, A-21207) https://www.thermofisher.com/antibody/product/Donkey-anti-Rabbit-IgG-H-L-Highly-Cross-Adsorbed-Secondary-Antibody-Polyclonal/A-21207

Eukaryotic cell lines

Policy information about cell lines

Cell line source(s)	THP1, HEK293T, MCF-7, SF9 and L929 cells were obtained from American Type Culture Collection. The mouse immortalized bone marrow-derived macrophages (iBMDM) were a gift from Dr. Inez Rogatsky (Hospital for Special Surgery, New York,NY)
Authentication	SF9, MCF-7, THP1, HEK293T and L929 cells were not validated beyond that provided by ATCC. iBMDM cells were not validated.
Mycoplasma contamination	Cell lines were regularly (every 3-4 months) tested for mycoplasma contamination using a commercial kit and the absence of mycoplasma contamination was verified before using the cells.
Commonly misidentified lines<br>(See ICLAC register)	None.

Animals and other organisms

Policy information about studies involving animals; ARRIVE guidelines recommended for reporting animal research

Laboratory animals	8-12 week old C57BL/6 male mice were obtained from the Mouse Breeding Core at UT Southwestern. 8-12 week old Parp1 null (Parp1-/-) male mice on a C57BL/6 background were used. These mice were generated described previously (de Murcia et al., 1997). All mice were maintained on a standard rodent chow diet with 12-hour light/12-hour dark cycle in a temperature-controlled environment (room temperature, 22°C; thermoneutrality, 30°C).
Wild animals	None.
Field-collected samples	None.
Ethics oversight	All animal use was performed with oversight from UT Southwestern's Institutional Animal Care and Use Committee.

Note that full information on the approval of the study protocol must also be provided in the manuscript.

ChIP-seq

Data deposition

☒ Confirm that both raw and final processed data have been deposited in a public database such as GEO.

☒ Confirm that you have deposited or provided access to graph files (e.g. BED files) for the called peaks.

Data access links<br>*May remain private before publication.*	Geo accession numbers for ChIp-seq data: GSE147951, GSE147952, GSE147953, GSE147954
Files in database submission	Control_rep1_Input<br>Control_rep2_Input<br>IFNγ_1h_rep1_Input<br>IFNγ_1h_rep2_Input<br>PJ34_rep1_Input<br>PJ34_rep2_Input<br>IFNγ_1h+PJ34_rep1_Input<br>IFNγ_1h+PJ34_rep2_Input<br>Control_rep1_STAT1_ChIP<br>Control_rep2_STAT1_ChIP<br>IFNγ_1h_rep1_STAT1_ChIP<br>IFNγ_1h_rep2_STAT1_ChIP<br>PJ34_rep1_STAT1_ChIP<br>PJ34_rep2_STAT1_ChIP<br>IFNγ_1h+PJ34_rep1_STAT1_ChIP<br>IFNγ_1h+PJ34_rep2_STAT1_ChIP<br><br>Control_rep1_Input<br>Control_rep2_Input<br>IFNγ_1h_rep1_Input<br>IFNγ_1h_rep2_Input<br>PJ34_rep1_Input<br>PJ34_rep2_Input<br>IFNγ_1h+PJ34_rep1_Input<br>IFNγ_1h+PJ34_rep2_Input<br>Control_rep1_H3K27ac_ChIP<br>Control_rep2_H3K27ac_ChIP<br>IFNγ_1h_rep1_H3K27ac_ChIP<br>IFNγ_1h_rep2_H3K27ac_ChIP<br>PJ34_rep1_H3K27ac_ChIP<br>PJ34_rep2_H3K27ac_ChIP<br>IFNγ_1h+PJ34_rep1_H3K27ac_ChIP<br>IFNγ_1h+PJ34_rep2_H3K27ac_ChIP<br><br>sh.ns_GFP_Control_rep1_Input<br>sh.ns_GFP_Control_rep2_Input<br>sh.ns_GFP_IFNγ_1h_rep1_Input<br>sh.ns_GFP_IFNγ_1h_rep2_Input<br>sh.Stat1_Stat1_Wt_Control_rep1_Input<br>sh.Stat1_Stat1_Wt_Control_rep2_Input<br>sh.Stat1_Stat1_Wt_IFNγ_1h_rep1_Input<br>sh.Stat1_Stat1_Wt_IFNγ_1h_rep2_Input<br>sh.Stat1_Stat1_DBDmut_Control_rep1_Input<br>sh.Stat1_Stat1_DBDmut_Control_rep2_Input<br>sh.Stat1_Stat1_DBDmut_IFNγ_1h_rep1_Input<br>sh.Stat1_Stat1_DBDmut_IFNγ_1h_rep2_Input<br>sh.Stat1_Stat1_TAmut_Control_rep1_Input<br>sh.Stat1_Stat1_TAmut_Control_rep2_Input<br>sh.Stat1_Stat1_TAmut_IFNγ_1h_rep1_Input<br>sh.Stat1_Stat1_TAmut_IFNγ_1h_rep2_Input<br>sh.ns_GFP_Control_rep1_STAT1<br>sh.ns_GFP_Control_rep2_STAT1<br>sh.ns_GFP_IFNγ_1h_rep1_STAT1<br>sh.ns_GFP_IFNγ_1h_rep2_STAT1<br>sh.Stat1_Stat1_Wt_Control_rep1_STAT1<br>sh.Stat1_Stat1_Wt_Control_rep2_STAT1<br>sh.Stat1_Stat1_Wt_IFNγ_1h_rep1_STAT1<br>sh.Stat1_Stat1_Wt_IFNγ_1h_rep2_STAT1<br>sh.Stat1_Stat1_DBDmut_Control_rep1_STAT1<br>sh.Stat1_Stat1_DBDmut_Control_rep2_STAT1<br>sh.Stat1_Stat1_DBDmut_IFNγ_1h_rep1_STAT1<br>sh.Stat1_Stat1_DBDmut_IFNγ_1h_rep2_STAT1

sh.Stat1_Stat1_TAmut_Control_rep1_STAT1
sh.Stat1_Stat1_TAmut_Control_rep2_STAT1
sh.Stat1_Stat1_TAmut_IFNγ_1h_rep1_STAT1
sh.Stat1_Stat1_TAmut_IFNγ_1h_rep2_STAT1

sh.Stat1_Stat1_Wt_Control_rep1_Input
sh.Stat1_Stat1_Wt_Control_rep2_Input
sh.Stat1_Stat1_Wt_IFNγ_1h_rep1_Input
sh.Stat1_Stat1_Wt_IFNγ_1h_rep2_Input
sh.Stat1_Stat1_DBDmut_Control_rep1_Input
sh.Stat1_Stat1_DBDmut_Control_rep2_Input
sh.Stat1_Stat1_DBDmut_IFNγ_1h_rep1_Input
sh.Stat1_Stat1_DBDmut_IFNγ_1h_rep2_Input
sh.Stat1_Stat1_TAmut_Control_rep1_Input
sh.Stat1_Stat1_TAmut_Control_rep2_Input
sh.Stat1_Stat1_TAmut_IFNγ_1h_rep1_Input
sh.Stat1_Stat1_TAmut_IFNγ_1h_rep2_Input
sh.Stat1_Stat1_Wt_Control_rep1_H3K27ac
sh.Stat1_Stat1_Wt_Control_rep2_H3K27ac
sh.Stat1_Stat1_Wt_IFNγ_1h_rep1_H3K27ac
sh.Stat1_Stat1_Wt_IFNγ_1h_rep2_H3K27ac
sh.Stat1_Stat1_DBDmut_Control_rep1_H3K27ac
sh.Stat1_Stat1_DBDmut_Control_rep2_H3K27ac
sh.Stat1_Stat1_DBDmut_IFNγ_1h_rep1_H3K27ac
sh.Stat1_Stat1_DBDmut_IFNγ_1h_rep2_H3K27ac
sh.Stat1_Stat1_TAmut_Control_rep1_H3K27ac
sh.Stat1_Stat1_TAmut_Control_rep2_H3K27ac
sh.Stat1_Stat1_TAmut_IFNγ_1h_rep1_H3K27ac
sh.Stat1_Stat1_TAmut_IFNγ_1h_rep2_H3K27ac

Genome browser session
(e.g. UCSC)

No longer applicable

Methodology

Replicates

ChIP-seq libraries were generated from two biological replicates for each condition.

Sequencing depth

Condition |Total reads| unique reads| length (bp) |paired/single end:

Control_rep1_Input 7473806 7196330 75 PE
Control_rep2_Input 15363241 14635056 75 PE
IFNγ_1h_rep1_Input 12939355 12541767 75 PE
IFNγ_1h_rep2_Input 12732628 12237326 75 PE
PJ34_rep1_Input 23623046 21302490 75 PE
PJ34_rep2_Input 18603147 17065665 75 PE
IFNγ_1h+PJ34_rep1_Input 16821718 16287794 75 PE
IFNγ_1h+PJ34_rep2_Input 13425845 12742631 75 PE
Control_rep1_STAT1_ChIP 14499606 13487541 75 PE
Control_rep2_STAT1_ChIP 6322107 5531106 75 PE
IFNγ_1h_rep1_STAT1_ChIP 11950884 11442616 75 PE
IFNγ_1h_rep2_STAT1_ChIP 12756982 12233249 75 PE
PJ34_rep1_STAT1_ChIP 20657508 14250050 75 PE
PJ34_rep2_STAT1_ChIP 19134201 16049076 75 PE
IFNγ_1h+PJ34_rep1_STAT1_ChIP 11715349 11224045 75 PE
IFNγ_1h+PJ34_rep2_STAT1_ChIP 15975238 15247868 75 PE
75 PE
Control_rep1_Input 8790607 8357074 75 PE
Control_rep2_Input 14615061 13820742 75 PE
IFNγ_1h_rep1_Input 12047163 11374572 75 PE
IFNγ_1h_rep2_Input 8480640 8087821 75 PE
PJ34_rep1_Input 11889280 11361269 75 PE
PJ34_rep2_Input 14473611 13741035 75 PE
IFNγ_1h+PJ34_rep1_Input 10237913 9793321 75 PE
IFNγ_1h+PJ34_rep2_Input 4747878 4562536 75 PE
Control_rep1_H3K27ac_ChIP 16262547 15264186 75 PE
Control_rep2_H3K27ac_ChIP 15051947 14155020 75 PE
IFNγ_1h_rep1_H3K27ac_ChIP 16677610 15563120 75 PE
IFNγ_1h_rep2_H3K27ac_ChIP 13116239 12396854 75 PE
PJ34_rep1_H3K27ac_ChIP 21089660 19745391 75 PE
PJ34_rep2_H3K27ac_ChIP 16128136 15108901 75 PE
IFNγ_1h+PJ34_rep1_H3K27ac_ChIP 13717730 12918641 75 PE
IFNγ_1h+PJ34_rep2_H3K27ac_ChIP 15596876 14631984 75 PE
75 PE
sh.ns_GFP_Control_rep1_Input 20392579 18809520 75 PE
sh.ns_GFP_Control_rep2_Input 12042164 11686252 75 PE

sh.ns_GFP_IFNγ_1h_rep1_Input 18781072 16853653 75 PE
sh.ns_GFP_IFNγ_1h_rep2_Input 13998477 13611997 75 PE
sh.Stat1_Stat1_Wt_Control_rep1_Input 19352060 17380992 75 PE
sh.Stat1_Stat1_Wt_Control_rep2_Input 19037026 18458759 75 PE
sh.Stat1_Stat1_Wt_IFNγ_1h_rep1_Input 18488501 16975150 75 PE
sh.Stat1_Stat1_Wt_IFNγ_1h_rep2_Input 14615527 14177930 75 PE
sh.Stat1_Stat1_DBDmut_Control_rep1_Input 18376598 17058809 75 PE
sh.Stat1_Stat1_DBDmut_Control_rep2_Input 20393105 19744562 75 PE
sh.Stat1_Stat1_DBDmut_IFNγ_1h_rep1_Input 16898137 15071952 75 PE
sh.Stat1_Stat1_DBDmut_IFNγ_1h_rep2_Input 18397122 17843831 75 PE
sh.Stat1_Stat1_TAmut_Control_rep1_Input 14754077 13925732 75 PE
sh.Stat1_Stat1_TAmut_Control_rep2_Input 22716663 22023443 75 PE
sh.Stat1_Stat1_TAmut_IFNγ_1h_rep1_Input 24399593 22051811 75 PE
sh.Stat1_Stat1_TAmut_IFNγ_1h_rep2_Input 29390730 28392631 75 PE
sh.ns_GFP_Control_rep1_STAT1 17020399 14880201 75 PE
sh.ns_GFP_Control_rep2_STAT1 12854125 11741561 75 PE
sh.ns_GFP_IFNγ_1h_rep1_STAT1 19352060 17380992 75 PE
sh.ns_GFP_IFNγ_1h_rep2_STAT1 12681108 12011611 75 PE
sh.Stat1_Stat1_Wt_Control_rep1_STAT1 15847767 12698721 75 PE
sh.Stat1_Stat1_Wt_Control_rep2_STAT1 11646348 10776974 75 PE
sh.Stat1_Stat1_Wt_IFNγ_1h_rep1_STAT1 12160426 10631328 75 PE
sh.Stat1_Stat1_Wt_IFNγ_1h_rep2_STAT1 11402809 10304081 75 PE
sh.Stat1_Stat1_DBDmut_Control_rep1_STAT1 14605077 12614002 75 PE
sh.Stat1_Stat1_DBDmut_Control_rep2_STAT1 12158804 11229365 75 PE
sh.Stat1_Stat1_DBDmut_IFNγ_1h_rep1_STAT1 14643480 12336265 75 PE
sh.Stat1_Stat1_DBDmut_IFNγ_1h_rep2_STAT1 11456708 10411122 75 PE
sh.Stat1_Stat1_TAmut_Control_rep1_STAT1 15034838 13568719 75 PE
sh.Stat1_Stat1_TAmut_Control_rep2_STAT1 12210885 11428240 75 PE
sh.Stat1_Stat1_TAmut_IFNγ_1h_rep1_STAT1 14941574 11669742 75 PE
sh.Stat1_Stat1_TAmut_IFNγ_1h_rep2_STAT1 10423063 9702972 75 PE
75 PE
sh.Stat1_Stat1_Wt_Control_rep1_Input 13864683 12771641 75 PE
sh.Stat1_Stat1_Wt_Control_rep2_Input 13700097 13029221 75 PE
sh.Stat1_Stat1_Wt_IFNγ_1h_rep1_Input 10992679 10576580 75 PE
sh.Stat1_Stat1_Wt_IFNγ_1h_rep2_Input 17752155 16384289 75 PE
sh.Stat1_Stat1_DBDmut_Control_rep1_Input 14368160 13714155 75 PE
sh.Stat1_Stat1_DBDmut_Control_rep2_Input 15756532 14715081 75 PE
sh.Stat1_Stat1_DBDmut_IFNγ_1h_rep1_Input 14922059 13993192 75 PE
sh.Stat1_Stat1_DBDmut_IFNγ_1h_rep2_Input 19036669 17579032 75 PE
sh.Stat1_Stat1_TAmut_Control_rep1_Input 14286870 13596825 75 PE
sh.Stat1_Stat1_TAmut_Control_rep2_Input 16098215 15334189 75 PE
sh.Stat1_Stat1_TAmut_IFNγ_1h_rep1_Input 15122543 14194664 75 PE
sh.Stat1_Stat1_TAmut_IFNγ_1h_rep2_Input 18633363 17622820 75 PE
sh.Stat1_Stat1_Wt_Control_rep1_H3K27ac 14584444 13847301 75 PE
sh.Stat1_Stat1_Wt_Control_rep2_H3K27ac 16821253 15659043 75 PE
sh.Stat1_Stat1_Wt_IFNγ_1h_rep1_H3K27ac 13545938 12481151 75 PE
sh.Stat1_Stat1_Wt_IFNγ_1h_rep2_H3K27ac 18319819 17478540 75 PE
sh.Stat1_Stat1_DBDmut_Control_rep1_H3K27ac 15591152 14935073 75 PE
sh.Stat1_Stat1_DBDmut_Control_rep2_H3K27ac 14502753 13027375 75 PE
sh.Stat1_Stat1_DBDmut_IFNγ_1h_rep1_H3K27ac 16444478 15715539 75 PE
sh.Stat1_Stat1_DBDmut_IFNγ_1h_rep2_H3K27ac 18141450 17036370 75 PE
sh.Stat1_Stat1_TAmut_Control_rep1_H3K27ac 11394119 10760383 75 PE
sh.Stat1_Stat1_TAmut_Control_rep2_H3K27ac 23700047 22454261 75 PE
sh.Stat1_Stat1_TAmut_IFNγ_1h_rep1_H3K27ac 16959686 16200033 75 PE
sh.Stat1_Stat1_TAmut_IFNγ_1h_rep2_H3K27ac 21144145 20006856 75 PE

Antibodies

STAT1 (Santa Cruz Biotech, sc-592) and Histone H3 (acetyl K27) (Abcam, ab4729) rabbit polyclonal antibodies were used for ChIP-seq.

Peak calling parameters

Relaxed peaks were called using MACS (ver. 2.1.0) and a default p-value = 1 x 10^-2 for each replicate and the input condition as a control. Final peaks were called based on overlap of the two replicates.

Data quality

Library complexity was measured using BEDTools (v 2.17.0) and all libraries were ensured to have met the minimum ENCODE data quality standards.

Software

ChIP-seq reads were were aligned to the mouse reference genome (mm10) using default parameters in Bowtie version 1.0.0. The aligned reads were filtered for quality and uniquely mappable reads using Samtools version 0.1.19 and Picard. Library complexity was measured using BEDTools version 2.17.0. Relaxed peaks were called using MACS version 2.1.0.
