# Supplementary material for: Nuclear ADP-ribosylation drives IFNγ-dependent STAT1α enhancer formation in macrophages
Source: Nat Commun. 2021 Jun 24;12:3931. doi: 10.1038/s41467-021-24225-2 (PMC8225886; doi:10.1038/s41467-021-24225-2)
Supplement: Supplementary file 5 — Source Data [file 41467_2021_24225_MOESM5_ESM.zip › Source Data.pptx]

## Slide 1
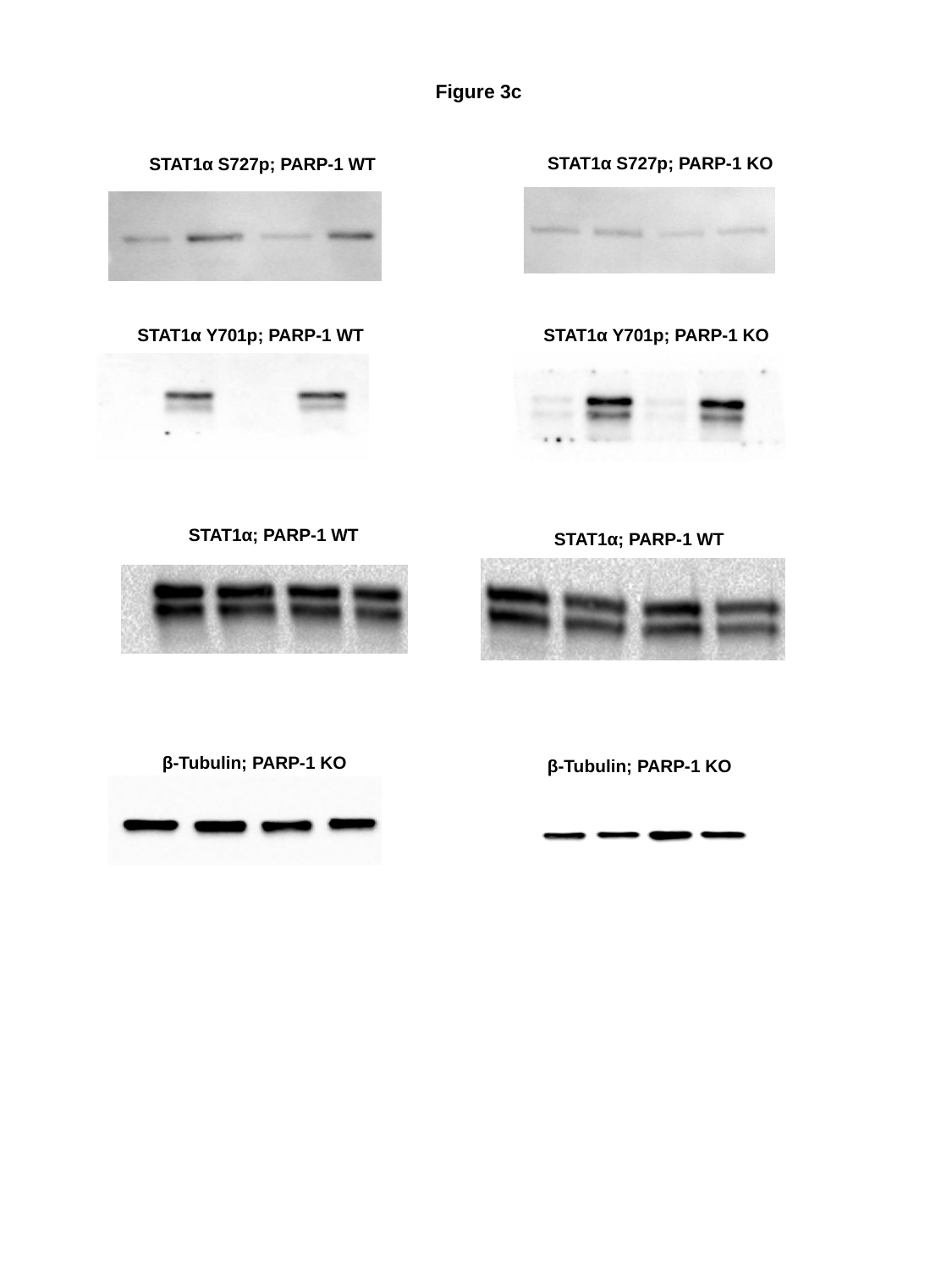

Figure 3c
STAT1α S727p; PARP-1 KO
STAT1α S727p; PARP-1 WT
STAT1α Y701p; PARP-1 WT
STAT1α Y701p; PARP-1 KO
STAT1α; PARP-1 WT
STAT1α; PARP-1 WT
β-Tubulin; PARP-1 KO
β-Tubulin; PARP-1 KO

## Slide 2
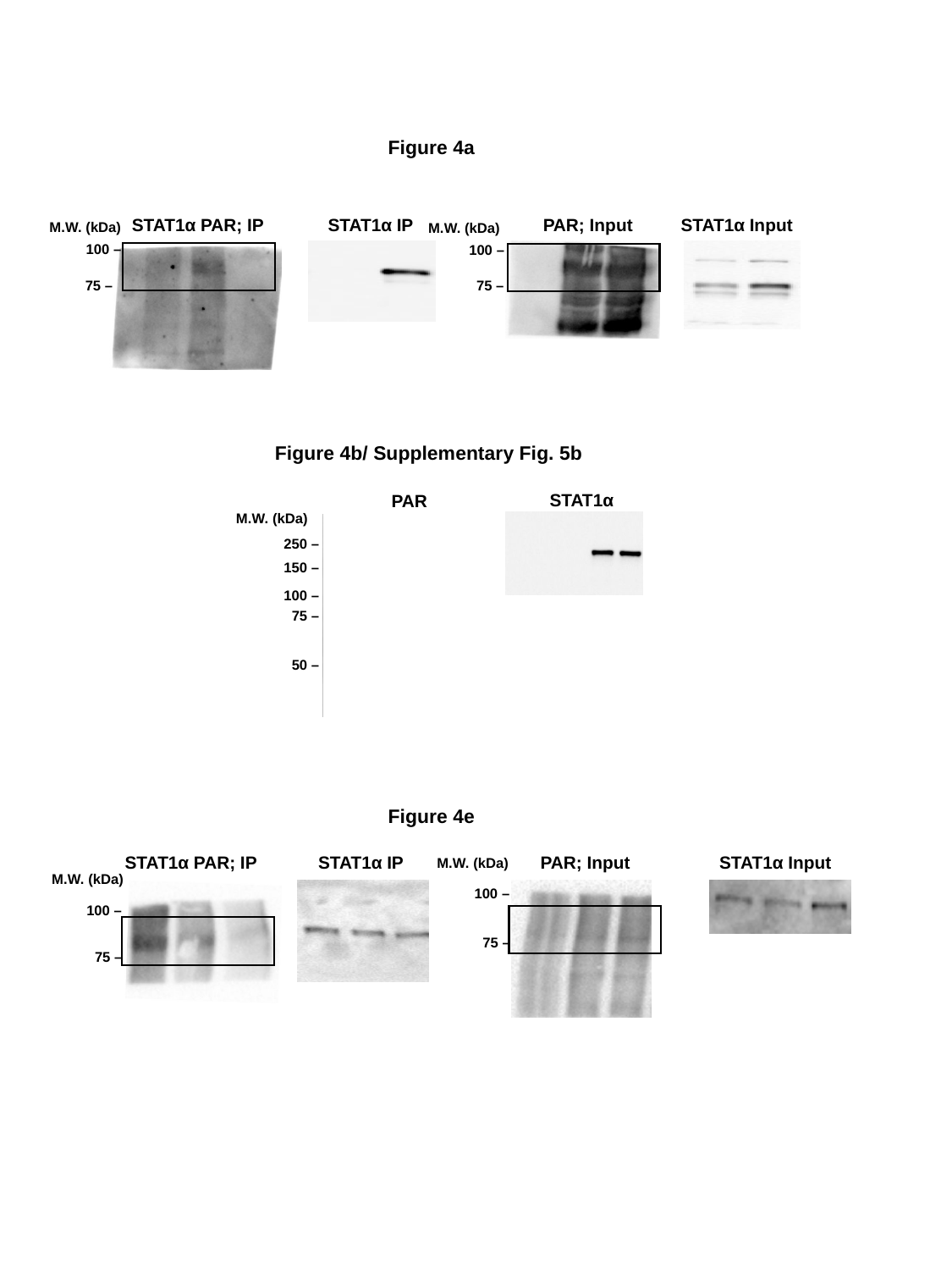

Figure 4a
STAT1α PAR; IP
STAT1α IP
PAR; Input
STAT1α Input
M.W. (kDa)
M.W. (kDa)
100 –
100 –
75 –
75 –
Figure 4b/ Supplementary Fig. 5b
STAT1α
PAR
M.W. (kDa)
250 –
150 –
100 –
75 –
50 –
Figure 4e
STAT1α PAR; IP
STAT1α IP
PAR; Input
STAT1α Input
M.W. (kDa)
M.W. (kDa)
100 –
100 –
75 –
75 –

## Slide 3
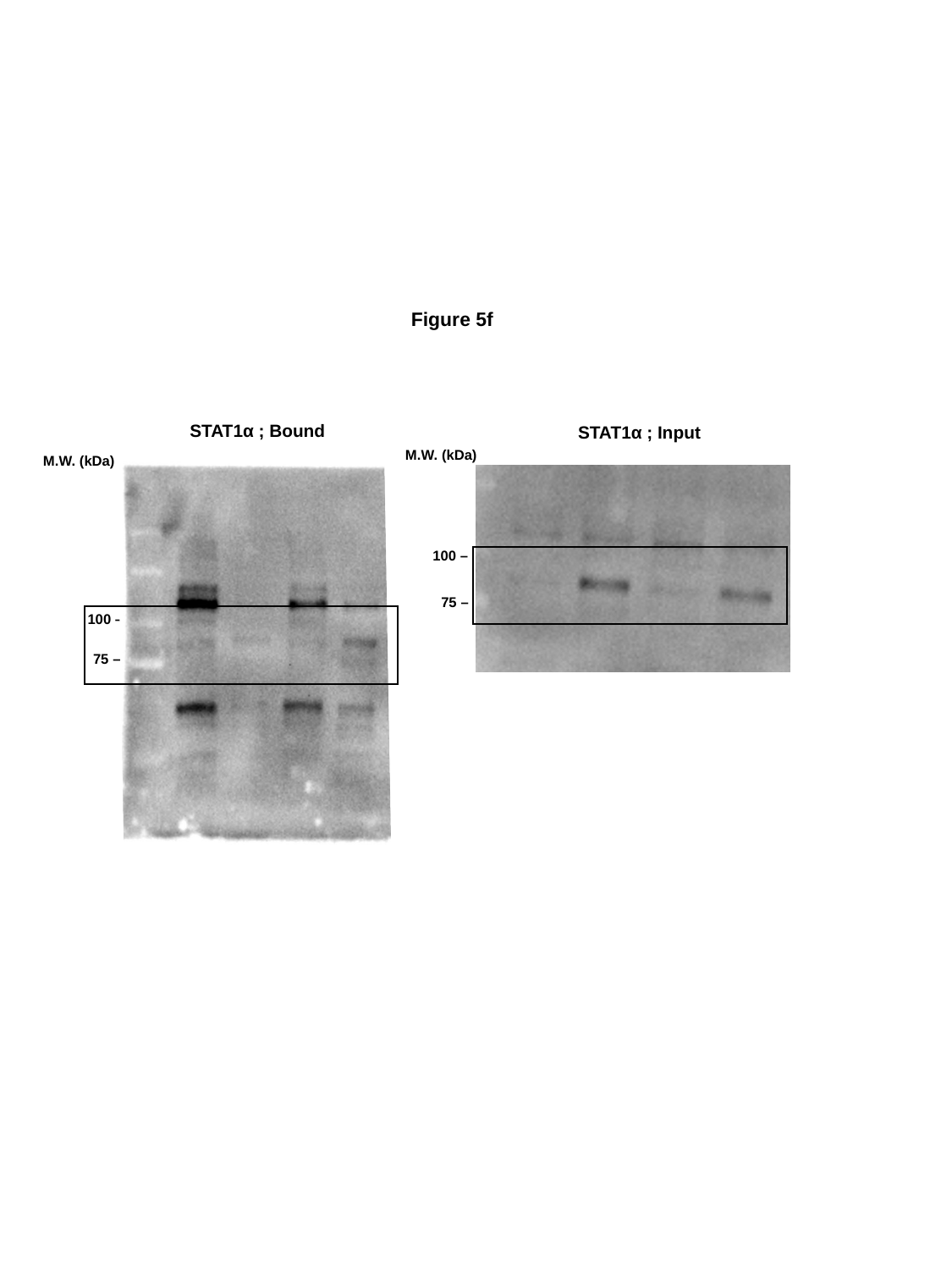

Figure 5f
STAT1α ; Bound
STAT1α ; Input
M.W. (kDa)
M.W. (kDa)
100 –
75 –
100 –
75 –

## Slide 4
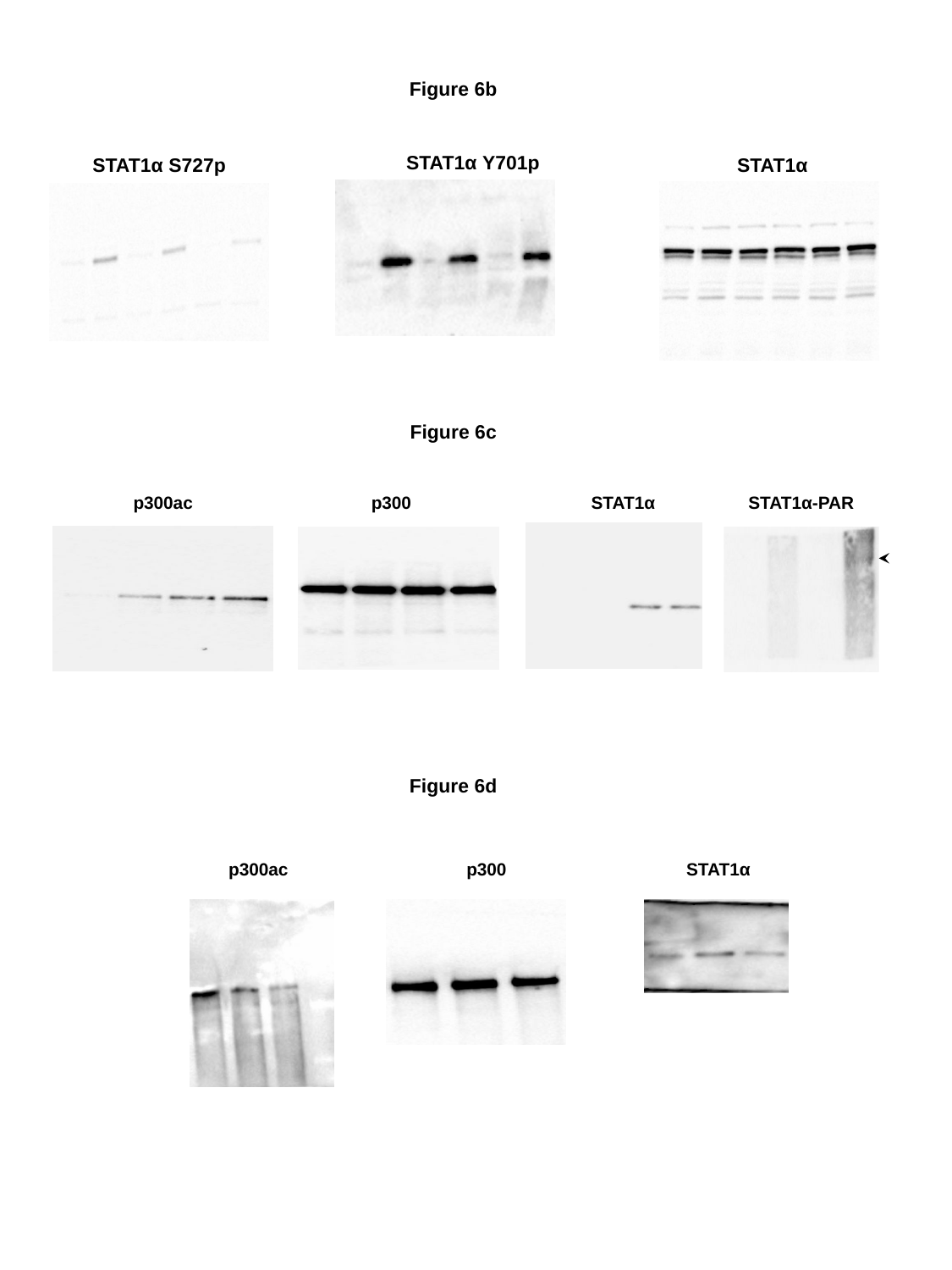

Figure 6b
STAT1α Y701p
STAT1α S727p
STAT1α
Figure 6c
p300ac
p300
STAT1α
STAT1α-PAR
Figure 6d
p300ac
p300
STAT1α

## Slide 5
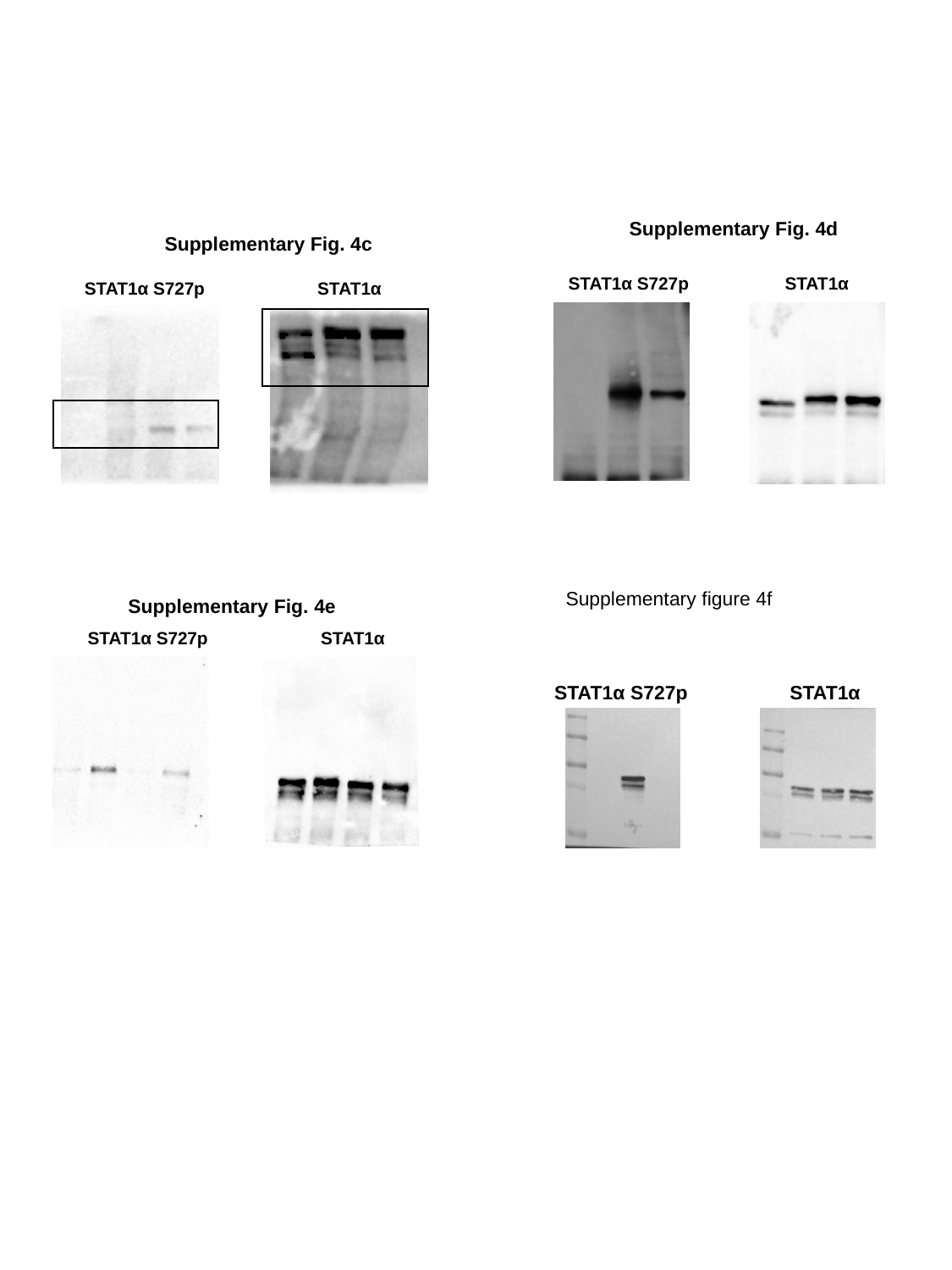

Supplementary Fig. 4d
Supplementary Fig. 4c
STAT1α S727p
STAT1α
STAT1α S727p
STAT1α
Supplementary figure 4f
Supplementary Fig. 4e
STAT1α S727p
STAT1α
STAT1α S727p
STAT1α

## Slide 6
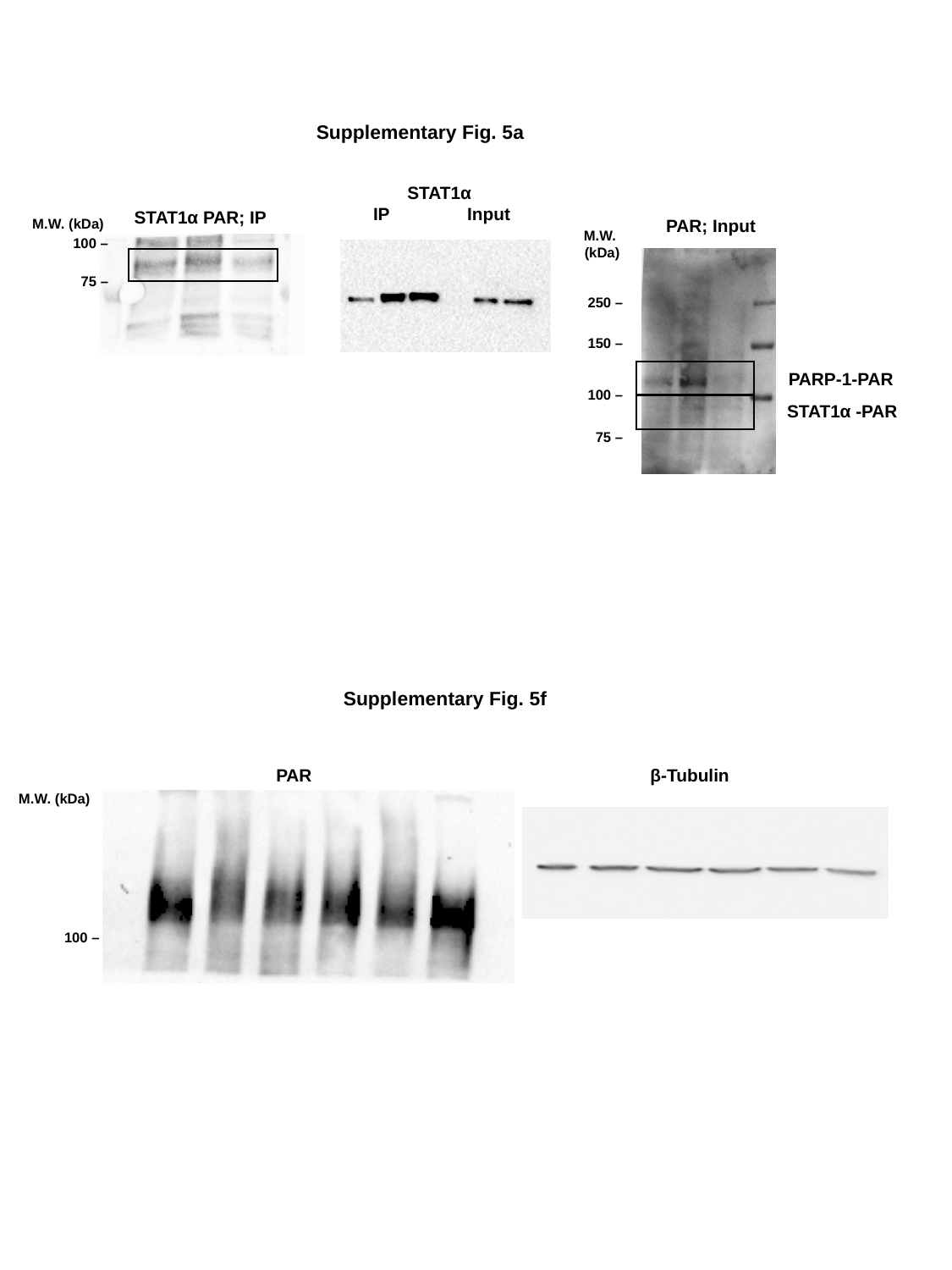

Supplementary Fig. 5a
STAT1α
IP Input
STAT1α PAR; IP
PAR; Input
M.W. (kDa)
M.W.
 (kDa)
100 –
75 –
250 –
150 –
PARP-1-PAR
100 –
STAT1α -PAR
75 –
Supplementary Fig. 5f
PAR
β-Tubulin
M.W. (kDa)
100 –

## Slide 7
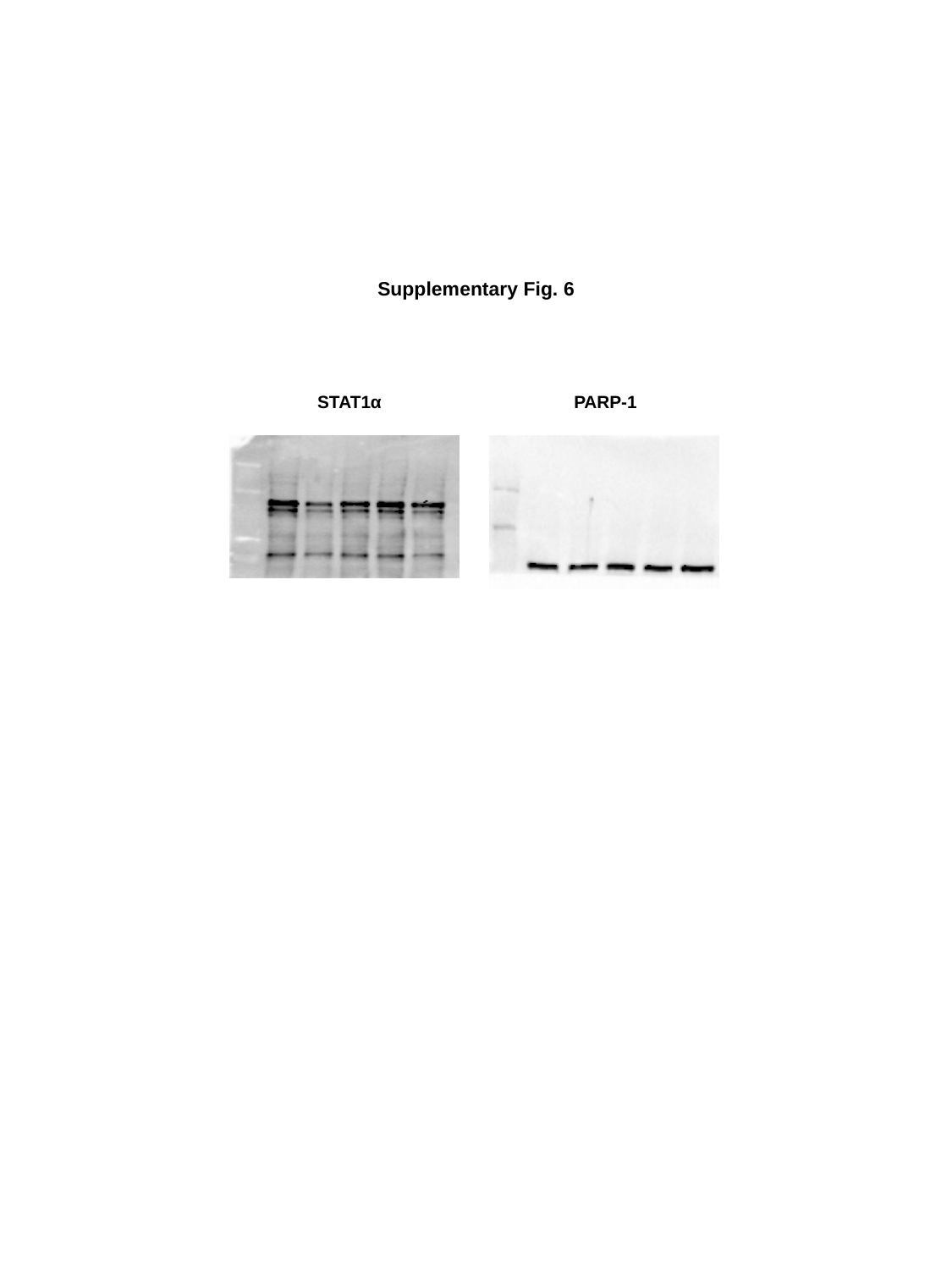

Supplementary Fig. 6
STAT1α
PARP-1

## Slide 8
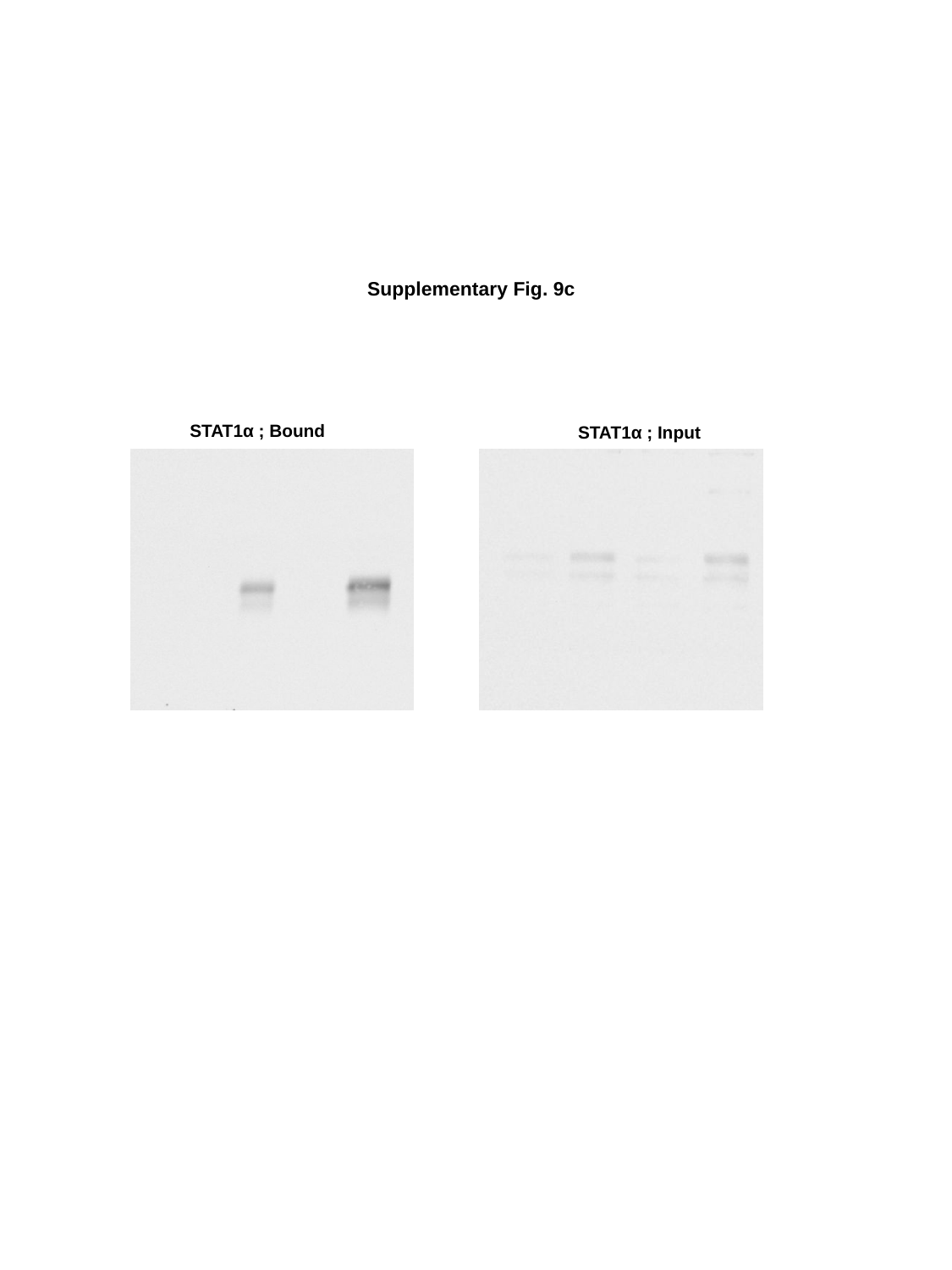

Supplementary Fig. 9c
STAT1α ; Bound
STAT1α ; Input

## Slide 9
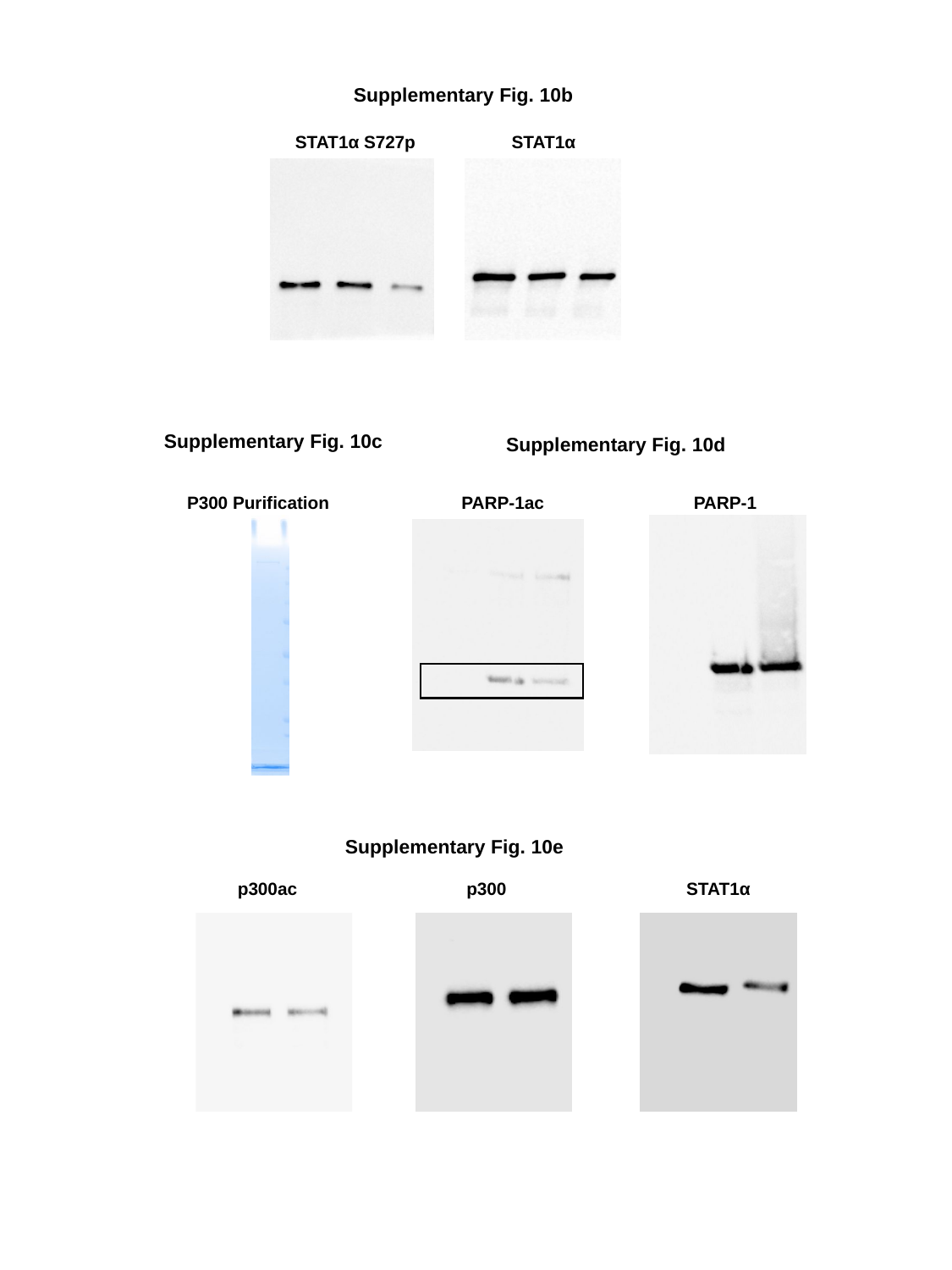

Supplementary Fig. 10b
STAT1α S727p
STAT1α
Supplementary Fig. 10c
Supplementary Fig. 10d
P300 Purification
PARP-1ac
PARP-1
Supplementary Fig. 10e
p300ac
p300
STAT1α
